# SORBS2 regulates diastolic function through cytoskeletal networks and calcium handling

**DOI:** 10.1038/s42003-026-10503-6

**Published:** 2026-06-17

**Authors:** Zexuan Wu, Camille Blandin, Jiehui Chen, Jiajin Li, Li He, Qing Ma, Michael M. Peters, Anna Ponek, Dogacan Yucel, Kevin Kit Parker, Fujian Lu, William T. Pu

**Affiliations:** 1 Department of Cardiology, Boston Children’s Hospital, Boston, MA USA; 2 Disease Biophysics Group, John A. Paulson School of Engineering and Applied Sciences, Harvard University, Boston, MA USA; 3 Harvard Stem Cell Institute, Cambridge, MA USA; 4 Present Address: Institutes of Biomedical Sciences, Department of Cardiology, Zhongshan Hospital, Shanghai Institute of Cardiovascular Diseases, Fudan University, Shanghai, China

**Keywords:** Heart failure, Cardiomyopathies

## Abstract

Diastolic dysfunction, defined by impaired relaxation and increased ventricular stiffness, is central to heart failure with preserved ejection fraction, yet cardiomyocyte-intrinsic mechanisms remain incompletely understood. Here, we show that SORBS2 is a vital component of murine cardiomyocyte adhesion complexes. Its genetic ablation in mice causes progressive diastolic dysfunction with preserved systolic function, accompanied by atrial enlargement and reduced survival. Postnatal cardiomyocyte-directed re-expression of SORBS2 restores diastolic indices and significantly improves longevity. Mechanistically, SORBS2 functions as an integrative scaffold linking adhesome integrity, cytoskeletal remodeling, and calcium homeostasis. SORBS2 deficiency increases microtubule detyrosination, reduces SERCA2 abundance, disrupts dyad-associated organization, which collectively impair active cardiomyocyte relaxation. Concurrently, this deficiency promotes extracellular matrix remodeling and myocardial fibrosis, driving passive ventricular stiffness. Pharmacological inhibition of microtubule detyrosination partially rescues relaxation defects. These findings establish SORBS2 as a key regulator of diastolic function and define a structural axis governing myocardial mechanics, offering potential therapeutic targets.

**Figure:**
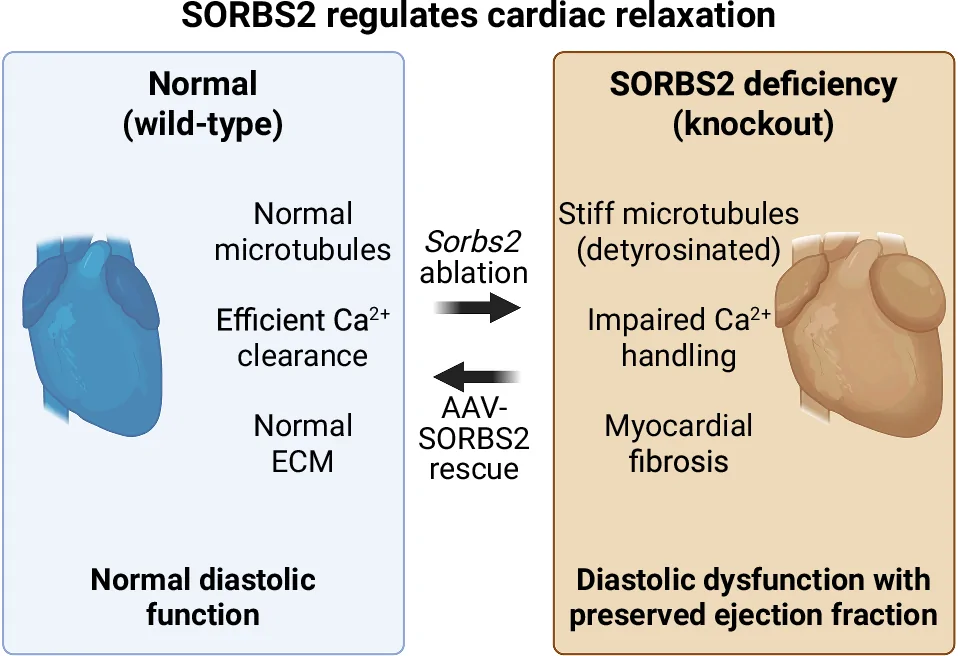

## Abstract (web summary)

Functional characterization of SORBS2, a component of the adhesome complex, sheds light on the regulation of cytoskeletal networks and calcium handling in the context of cardiac diastolic function.

## Introduction

Diastolic dysfunction, characterized by impaired left ventricular relaxation and increased myocardial stiffness, is a fundamental physiological impairment that underlies diverse forms of heart failure, including heart failure with preserved ejection fraction (HFpEF)^1,2^. Although HFpEF constitutes nearly half of all heart failure cases^3^, the cardiomyocyte-intrinsic mechanisms by which adhesion-associated cytoskeletal architecture governs diastolic function remain poorly defined.

The cytoskeletal adaptor protein SORBS2 (Sorbin and SH3 domain-containing protein 2, also known as Arg-binding protein 2 (ArgBP2)) plays a crucial role in cell adhesion, migration, and signaling as a component of the focal adhesion complex^4–6^. Within the heart, SORBS2 is predominantly expressed in cardiomyocytes^7^. By light microscopy, SORBS2 protein localization overlaps with the sarcomere Z-discs and the intercalated discs, and protein-protein interaction studies indicate that SORBS2 interacts with key components of focal adhesion complexes, including α-actinin, vinculin, and focal adhesion kinase^4,7^.

Previous studies of human heart tissue and experimental models have shown that SORBS2 is upregulated under pathological conditions such as dilated cardiomyopathy, hypertrophic cardiomyopathy, pressure overload, and myocardial infarction^7,8^. At baseline, *Sorbs2* knockout caused increased fibrosis, cardiomyocyte hypertrophy, and increased susceptibility to systolic dysfunction in the context of pressure overload^7,8^. Progressive systolic dysfunction was noted after 30 weeks of age, leading to these mice being described as having dilated cardiomyopathy^8^. Another group reported that SORBS2 localized to cardiomyocyte intercalated discs, and that *Sorbs2* knockout disrupted intercalated disc structure and manifested features of arrhythmogenic cardiomyopathy^9^.

In this study, we investigated the role of SORBS2 in cardiac homeostasis using a constitutive murine knockout model. We found that over the first 6 months of life, *Sorbs2* inactivation caused premature death and severe diastolic dysfunction with preserved ejection function. Our mechanistic investigations of *Sorbs2* suggest that its ablation causes dysregulation of cytoskeletal networks and cardiomyocyte Ca^2+^ handling, linking SORBS2 and the adhesome complex to maintenance of cardiac diastolic function.

## Results

### SORBS2 deficiency causes severe diastolic dysfunction with preserved ejection fraction

**Fig. 1:**
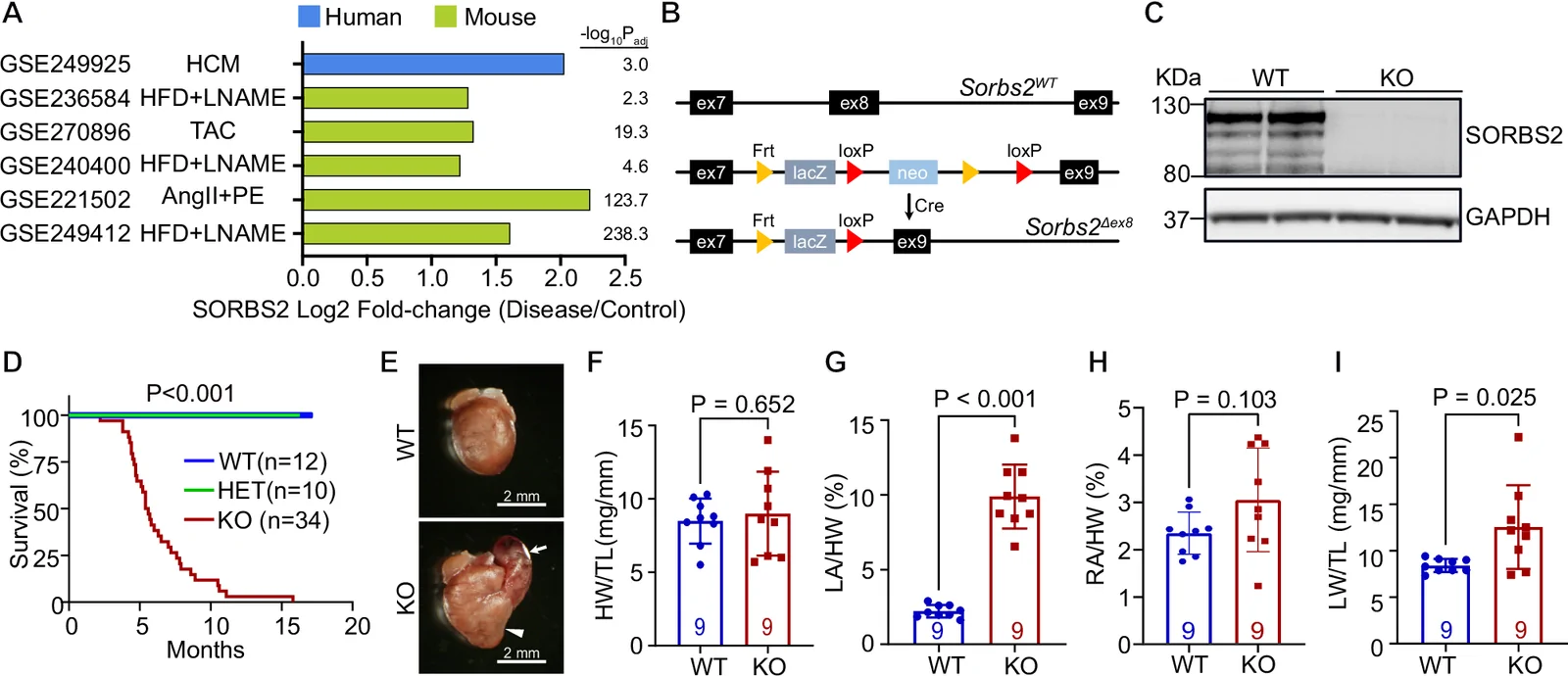
Characterization of *Sorbs2* null mice. **A** *Sorbs2* transcript levels in human HCM and multiple murine models of cardiac stress and remodeling. **B** Murine *Sorbs2* exons 7-9, showing structure of the *Sorbs2*^*Δex8*^ null allele used in this study. **C** Immunoblot showing SORBS2 expression in KO and WT hearts. **D** Survival curve of KO, HET, and WT mice. Mantel-Cox. **E** Gross morphology of KO and WT hearts. Arrow, massively enlarged left atrium of KO heart. Arrowhead, concave curvature of left ventricular free wall of KO heart. **F–I** KO vs WT heart weight to tibia length (HW/TL), left atrium to heart weight (LA/HW), right atrium to heart weight (RA/HW), and lung weight to tibia length (LW/TL) *t-*test. Plots show mean ± SD.

We interrogated public single-nucleus sequencing data of the human heart^10^ to determine the cell types that express SORBS2. SORBS2 was robustly and selectively expressed in ventricular and atrial cardiomyocytes (Supplementary Fig. 1A). SORBS2, RYR2, and JPH2 immunostaining revealed SORBS2 localized at intercalated discs, in a striated pattern consistent with cardiac dyads, which are immediately adjacent to sarcomere Z-discs, and at the cell periphery (Supplementary Fig. 1B, C and Supplementary Data 1). Re-analysis of published transcriptomic datasets showed that SORBS2 is consistently upregulated across multiple human and murine cardiac stress and remodeling models (Fig. 1A).

To investigate the functional role of SORBS2 in cardiac homeostasis, we studied mice globally lacking *Sorbs2* exon 8 (Fig. 1B). These *Sorbs2*^*Δex8/Δex8*^ (KO) mice did not detectably express SORBS2 protein (Fig. 1C). KO mice survived normally to weaning but displayed poorer long-term survival compared with wild type (WT) and *Sorbs2*^*Δex8/+*^ (Het) mice (Fig. 1D). Premature death started at 3 months of age, and 50% died by 5.4 months. Male and female survival were comparably decreased in KO mice (Supplementary Fig. 1D).

At 6 months of age, KO mice had lower body weight compared to WT (Supplementary Fig. 1E), while their tibia lengths were comparable (Supplementary Fig. 1F). KO hearts had dramatically abnormal morphology, with an enormous left atrium (arrow, Fig. 1E) and a concave left ventricular silhouette (arrowhead, Fig. 1E). There was no increase in heart weight normalized to tibia length (Fig. 1F). The KO right atrium was not significantly enlarged, but the KO left atrium was 4.6-fold larger than WT as a percentage of heart weight (Fig. 1G, H). Thrombi were frequently observed in the distended left atria. The lung weight of KO mice was also significantly elevated (Fig. 1I), suggestive of pulmonary edema and decompensated left ventricular heart failure.

**Fig. 2:**
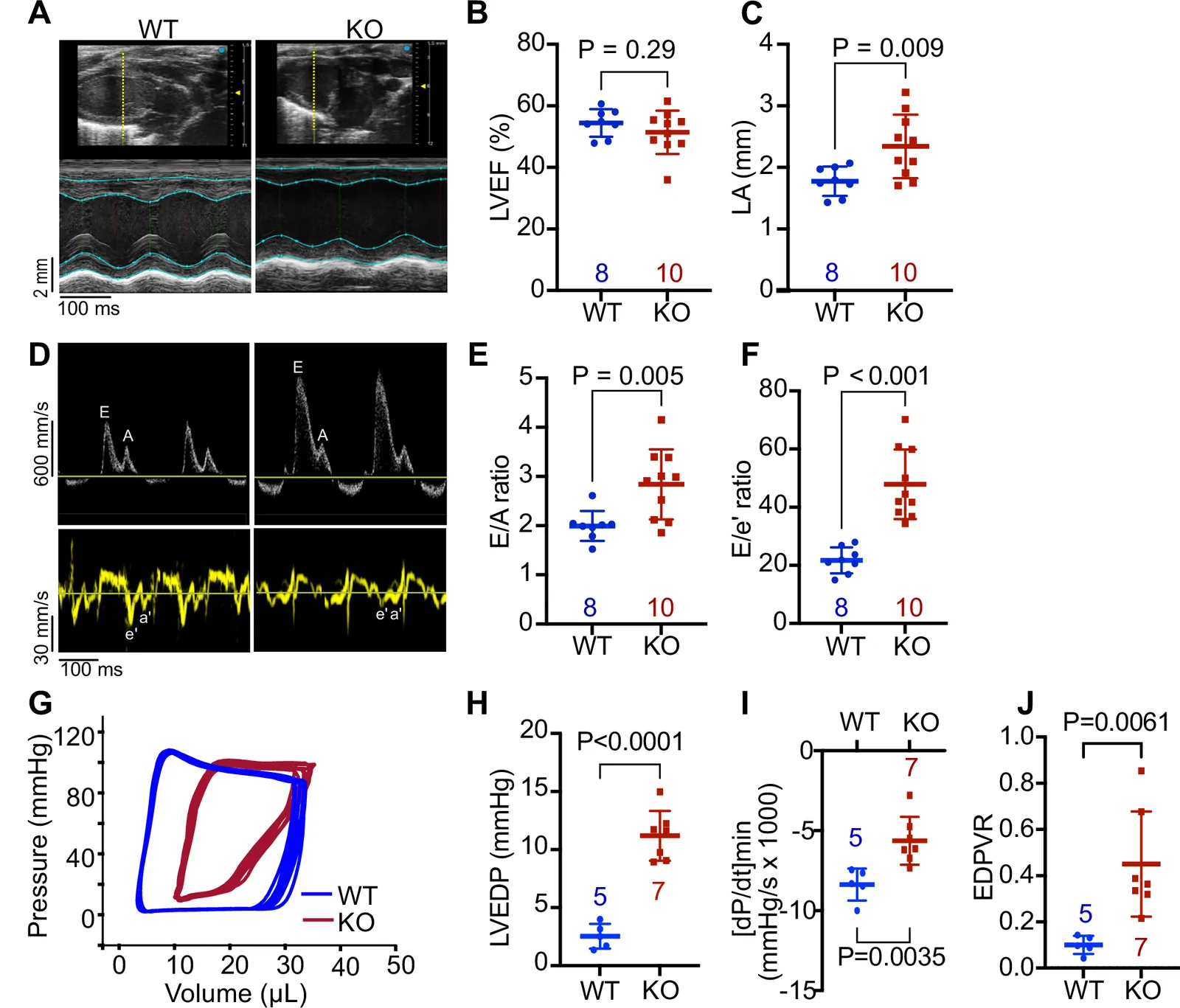
Functional analysis of *Sorbs2* KO and WT hearts at 6 months of age. **A** Representative echocardiographic B-mode image and accompanying M-mode trace at the yellow dashed line. Blue lines highlight the myocardial boundaries. **B**, **C** Left ventricular ejection fraction (LVEF, **B**) and left atrial diameter (LA) *t-*test. **D** Representative Doppler echocardiographic images showing transmitral flow (white) and diastolic mitral annular measures of diastolic function: early to late mitral inflow velocity (E/A) ratio, and early mitral inflow to early mitral annular velocity (E/e’) ratio. Quantification of the E/A ratio (**E**) and E/e′ ratio (**F**), *t-*test. **G** Intracardiac hemodynamic measurements on WT and KO mice. Representative pressure-volume curves are shown. **H–J** Hemodynamic assessment of diastolic function. **H** Left ventricular end-diastolic pressure (LVEDP). **I** Minimum rate of change of pressure over time ([*dP/dt*]_*min*_). **J** End-diastolic pressure volume relationship (EDPVR) *t*-test. Plots show mean ± SD.

We assessed cardiac size and function by echocardiography in 6-month-old mice (Fig. 2A–F and Supplementary Table 1). KO mice had preserved systolic function, as assessed by the left ventricular ejection fraction (Fig. 2A, B). Their left atria were dilated, consistent with the post-mortem observations (Fig. 2C). The ratio of early to late diastolic mitral inflow velocity (E/A ratio) was significantly elevated in KO mice (Fig. 2D, E), consistent with diastolic dysfunction. Another measure of diastolic dysfunction, the ratio of the early mitral inflow velocity to the early diastolic mitral annular velocity (E/e’), was likewise significantly elevated (Fig. 2F).

While echocardiography allows non-invasive assessment of diastolic function, cardiac catheterization is the gold standard method. We obtained intracardiac pressure and volume recordings on 6-month-old KO and WT mice (Fig. 2G). Systolic function parameters (the maximum rate of change of pressure over time ([*dP/dt*]_max_), and the end-systolic pressure-volume relationship) did not differ between groups (Supplementary Fig. 2A, B). In contrast, the left ventricular end diastolic pressure, [*dP/dt*]_min_, and the left ventricular end diastolic pressure volume relationship, measures of diastolic function, all indicated impaired diastolic function in KO mice (Fig. 2H–J).

To assess the temporal development of these abnormalities, a cohort of mice was analyzed at 3 months of age. At this age, body weight, heart weight, and tibia length were comparable between genotypes (Supplementary Fig. 2C–E). The right atrium was enlarged (Supplementary Fig. 2F), and the left atrium tended to be enlarged, but this difference did not reach statistical significance (*P* = 0.06; Supplementary Fig. 2G). Lung weight was comparable between groups (Supplementary Fig. 2H). By echocardiography, KO mice had normal systolic function (Supplementary Fig. 2I; Supplementary Table 2) and the left atrium was not yet enlarged (Supplementary Fig. 2J). The E/A ratio was not significantly different, but E/e’ was significantly elevated in KO (Supplementary Fig. 2K, L). These data suggest that at 3 months of age, KO mice have early diastolic dysfunction indicated by elevated E/e’, preserved E/A and LA size, and normal lung weight. Consistent with this sequence, gross morphology remained largely unremarkable at 6 weeks and 3 months, but atrial remodeling became prominent with age—manifesting as marked LA dilation by 6 months and progressing to biatrial enlargement with RV dilation and frequent LA thrombi by 9 months (Supplementary Fig. 3).

Together, these findings demonstrate that through 6 months of age, *Sorbs2* inactivation causes diastolic dysfunction with preserved ejection fraction.

### Cardiomyocyte SORBS2 is required to maintain diastolic function

**Fig. 3:**
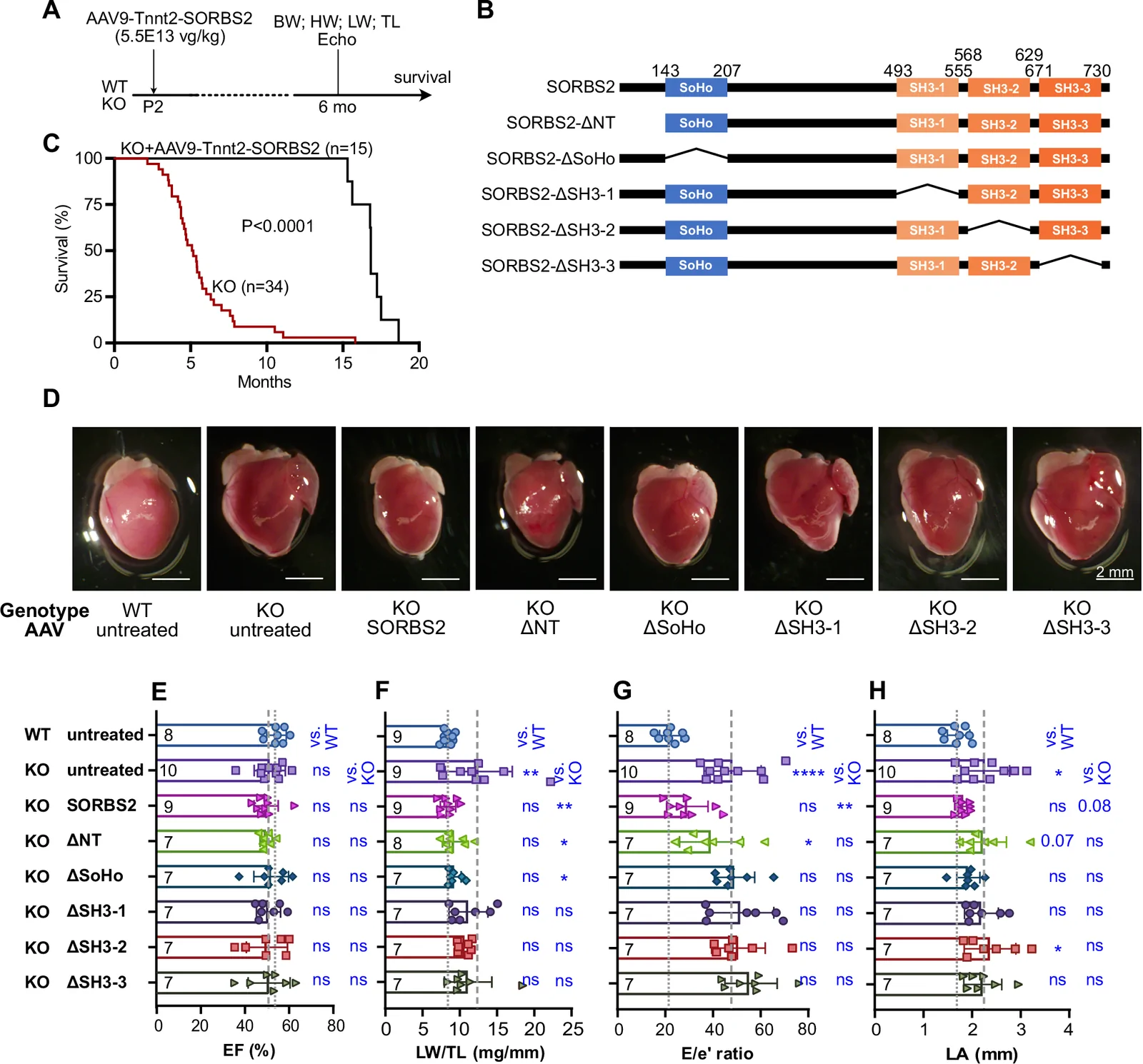
SORBS2 expression in cardiomyocytes improves survival and cardiac function. **A** Schematic outline of rescue experiments. AAV9 expressing *Sorbs2* from the cardiomyocyte-selective Tnnt2 promoter was given at postnatal day 2. We analyzed survival in one cohort. At 6 mo, echo and organ weights were measured in a second cohort. **B** Schematic of the *Sorbs2* cDNA and deletion mutants. **C** Survival of KO mice with and without treatment with AAV-Tnnt2-Sorbs2. The untreated KO mice are the same as from Fig. 1D. Mantel-Cox. **D** Representative gross heart morphology. Scale bar = 2 mm. **E–H** Effect of neonatal treatment with AAV9 expressing indicated *Sorbs2* cDNA on ejection fraction (EF%), lung weight to tibia length (LW/TL), ratio of early mitral inflow to mitral annular velocity (E/e’), and left atrial diameter (LA). Dashed and dotted gray lines indicate mean for untreated KO and WT, respectively. Plots show mean ± SD. One-way ANOVA with Dunnett’s post hoc test compared to untreated WT (vs. WT) or KO (vs. KO). ns, not significant. *, *P* < 0.05. **, *P* < 0.01. ****,* P* < 0.0001.

Since *Sorbs2*^*Δex8*^ is a constitutive null allele, KO mice globally lack SORBS2, and SORBS2 deficiency extends from development into adulthood. To assess whether postnatal expression of SORBS2 in cardiomyocytes is sufficient to rescue cardiac function and survival, we treated neonatal KO mice with 5.5 × 10^10^ vg/g AAV9-Tnnt2-SORBS2, in which the cardiomyocyte-selective Tnnt2 promoter drives expression of *Sorbs2* (Fig. 3A). We used a *Sorbs2* isoform that includes the sorbin homology (SoHo) domain, a protein-protein interaction domain that binds to the lipid raft protein flotillin, and three SH3 protein-protein interaction domains, but lacks a neuron-specific exon (Fig. 3B and Supplementary Table 3). AAV-mediated postnatal expression of *Sorbs2* increased the survival of KO mice by over 10 months (Fig. 3C), indicating that the early death of *Sorbs2* KO mice at 5-6 months was largely due to the absence of *Sorbs2* in postnatal cardiomyocytes rather than a developmental disorder^11^ or the lack of *Sorbs2* in other cell types.

Based on the dramatically enhanced survival of KO mice treated with AAV-Tnnt2-SORBS2, we developed a complementation assay to investigate the regions of SORBS2 that are required for its cardiac activity. We constructed AAV vectors that expressed full-length mouse *Sorbs2* cDNA, or mutant *Sorbs2* cDNA encoding SORBS2 proteins with deletions of the N-terminal region (ΔΝΤ) or the SoHo, SH3-1, SH3-2, or SH3-3 domains (Fig. 3B and Supplementary Table 3). KO mice were treated with AAV at postnatal day 2 (P2). Immunofluorescence analysis revealed that full-length SORBS2 and all truncation mutants exhibited a preserved striated distribution aligned with ACTN2-positive Z-discs (Supplementary Fig. 4A). These findings indicate that the differential rescue capacity of SORBS2 mutants is unlikely to result from its gross mislocalization. Western blotting (Supplementary Fig. 4B) showed that the AAV-expressed SORBS2 protein was more highly expressed than endogenous SORBS2. SORBS2-ΔNT, SORBS2-ΔSoHo, SORBS2-ΔSH3-2, and SORBS2-ΔSH3-3 were also expressed at greater levels than endogenous, whereas SORBS2-ΔSH3-1 was expressed at a lower level.

To assess the ability of SORBS2 or its deletion mutants to rescue KO heart function, we performed echocardiography and assessed gross morphology and tissue weights at 6 months of age (Fig. 3D–H, Supplementary Fig. 4C, and Supplementary Tables 4-5). Expression of SORBS2 or SORBS2 deletion mutants did not affect ventricular systolic function (Fig. 3E). KO mice treated with AAV-Tnnt2-SORBS2 had normal gross heart morphology (Fig. 3D) and normal lung weight to tibial length ratio (Fig. 3F), E/e’ ratio (Fig. 3G), and left atrial size (Fig. 3H, Supplementary Fig. 4C, and Supplementary Table 4-5). These results, combined with AAV-Tnnt2-SORBS2 enhancement of KO survival, indicate that postnatal cardiomyocyte expression of SORBS2 is sufficient to rescue the diastolic dysfunction of KO mice. Thus, diastolic dysfunction in KO mice is due to a lack of SORBS2 in postnatal cardiomyocytes.

In KO mice treated with AAV-SORBS2-ΔNT and AAV-SORBS2-ΔSoHo, lung weight normalized to tibia length (LW/TL) was comparable to WT (Fig. 3F), as with AAV-SORBS2. However, E/e’ and LA size were incompletely normalized, suggesting that these mutants have partial activity in maintaining heart diastolic function. Deletion of each SH3 domain did not alter these parameters compared to untreated KO mice (Fig. 3F–H and Supplementary Fig. 4C), suggesting that each SH3 domain is essential for SORBS2 function in cardiac relaxation.

Together, our data demonstrate that SORBS2 is required postnatally in cardiomyocytes to maintain heart diastolic function. The NT and SoHo regions may be partially dispensable for this function, whereas the SH3-1, SH3-2, and SH3-3 domains are essential.

### SORBS2 interacts with focal adhesion complex proteins

**Fig. 4:**
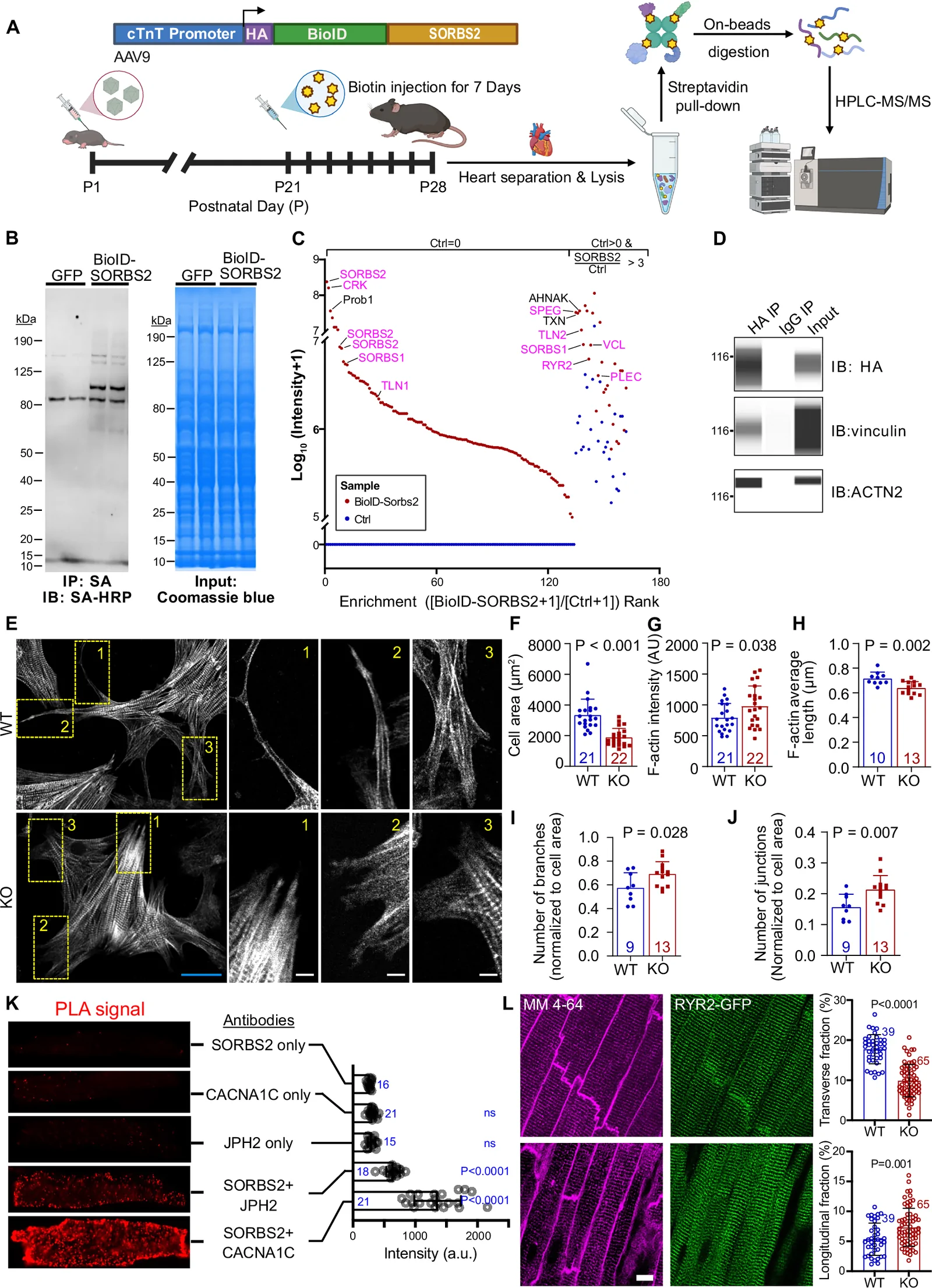
Characterization of SORBS2 neighborhoods through proximity proteomics. **A** Experimental design. AAV9 was used to deliver *Sorbs2* labeled with HA and BioID to neonatal pups. Mice were treated with biotin by daily intraperitoneal injection for 7 days and then the heart was collected at P28. Biotinylated proteins were purified using streptavidin beads, fragmented into peptides, and analyzed by mass spectrometry. Created using Biorender.com. **B** Immunoblot of heart lysate after purification on streptavidin and probed with streptavidin linked to horseradish peroxidase (HRP). Input protein gel was stained with Coomassie blue. **C** Mass spectrometry results. Peptide counts were plotted versus enrichment rank, stratified by whether or not peptide counts were detected in the control sample. Magenta indicates proteins mentioned in the text. **D** SORBS2 co-immunoprecipitated vinculin and α-actinin. Capillary western blot images are shown. **E–J** *Sorbs2* knockout altered the cytoskeletal organization of neonatal cardiomyocytes. WT and KO neonatal cardiomyocytes were stained with phalloidin. Each point represents one cell from 2 independent experiments with 3 separate wells per replicate. **E** Representative image with boxed areas enlarged to the right. Blue bar = 30 µm. White bar = 5 µm. **F** Quantification of cell area. Mann–Whitney test. **G–J** Quantification of properties of actin filaments *t-*test. **K** Proximity ligation assay (PLA) using indicated antibody combinations. Left, representative images. Right, Quantification. Each point represents one cell. One-way ANOVA with Dunnett’s post hoc test. **L** Heart in situ confocal imaging. T-tubules and junctional SR were visualized with MM 4-64 and RYR2-GFP, respectively. Images were analyzed to quantify transverse and longitudinal T-tubule components. Each point represents one image *t-*test. Graphs show mean ± SD.

SORBS2 contains multiple protein-protein interaction domains. To further delineate the SORBS2 interactome, we performed proximity proteomics by using AAV to express SORBS2 fused to BirA*, an engineered biotin ligase that releases biotin free radicals, resulting in biotinylation of proteins within ~10 nm^12^ (Fig. 4A and Supplementary Fig. 5A). AAV expressing GFP was used as a negative control. Biotinylated proteins, purified by binding to immobilized streptavidin, were identified by mass spectrometry (Fig. 4B, C, Supplementary Table 6 and Supplementary Data 2). Peptides derived from SORBS2 were among the most enriched. Consistent with prior studies of the integrin adhesome^4–6^ and of proteins that co-immunoprecipitate with SORBS2^7^, other highly enriched peptides were derived from components of focal adhesion complexes, which link the cardiomyocyte cytoskeleton to the extracellular matrix. Among the proteins enriched by BirA*-SORBS2 were talin (TLN1 and TLN2), vinculin (VCL), SORBS1, Plectin (PLEC), and CRK. TLN1 and TLN2 are concentrated at focal adhesions and link integrins to the actin cytoskeleton directly or indirectly by interacting with vinculin and α-actinin^13^. Vinculin is a focal adhesion protein that anchors the cytoskeleton to the plasma membrane by binding both talin and actin^14^. Like SORBS2, SORBS1 is an adaptor protein localized at focal adhesions that is involved in cell adhesion and cytoskeletal regulation^15^. Plectin, essential in cardiomyocytes for sarcomere and intercalated disc assembly, links the actin cytoskeleton to microtubules and intermediate filaments^16^. CRK is an oncogene that bridges tyrosine kinase signaling to downstream pathways to regulate cell adhesion and cytoskeletal organization^17^. We performed co-immunoprecipitation assays to confirm the interaction of SORBS2 with VCL and ACTN2. In cardiomyocytes transduced with AAV9-Tnnt2-HA*-*SORBS2, we detected VCL and ACTN2 in the HA-SORBS2 immunoprecipitate (Fig. 4D). Confocal imaging showed that VCL at focal adhesions at the cell periphery co-localized with SORBS2, while these proteins did not co-localize within the cell interior (Supplementary Fig. 5B).

We next investigated the effect of SORBS2 deficiency on the function of focal adhesions and cytoskeletal organization. We plated *Sorbs2* KO and WT neonatal cardiomyocytes on laminin-coated glass coverslips and stained for filamentous actin (F-actin; Fig. 4E). KO neonatal cardiomyocytes were smaller than WT controls, consistent with reduced cell spreading and weakened cell adhesion (Fig. 4F). F-actin staining in KO cardiomyocytes was more intense than WT (Fig. 4G), suggesting actin aggregation at the cell periphery at sites of detachment. Additionally, F-actin filaments became shorter (Fig. 4H), and their organization was disrupted, as evidenced by an increased number of filament branches and junctions (Fig. 4I, J). These data indicate that SORBS2 is a component of cardiomyocyte focal adhesion complexes and is essential for their normal function to regulate cytoskeletal organization.

BirA*-SORBS2 biotinylated proteins also included dyadic proteins SPEG and RYR2. Proximity proteomics of the RYR2 complex^18^ and Cav1.2^19^ reciprocally identified SORBS2 (and SPEG), suggesting that a subset of SORBS2 localizes to cardiac dyads and may influence Ca^2+^ handling. We used the proximity ligation assay (PLA), which yields signal when two antibodies bind to targets that are within ~50 nm, to validate that SORBS2 was located within the vicinity of dyadic proteins junctophilin (JPH2) and Ca_v_1.2 (CACNA1C). We detected PLA signals with SORBS2 + JPH2 and SORBS2 + CACNA1C antibody pairs, but not SORBS2, JPH2, or CACNA1C antibody alone (Fig. 4K). Immunoblotting revealed a significant reduction in JPH2 protein abundance in *Sorbs2* KO hearts (Supplementary Fig. 5C), accompanied by disrupted JPH2 spatial organization with loss of the normal striated dyadic pattern in heart sections and isolated cardiomyocytes (Supplementary Fig. 5D, E). Consistent with dyadic destabilization, in situ confocal imaging of T-tubules and RYR2-GFP^20^ demonstrated reduced regularity of both transverse and longitudinal T-tubules in KO hearts (Fig. 4L), indicating that SORBS2 is required to maintain dyad architecture. Together, these data indicate that cardiomyocyte SORBS2 is enriched at focal adhesions and within cardiac dyads, where it plays an essential role in organizing dyads.

### Mechanisms of diastolic dysfunction in Sorbs2 KO hearts

**Fig. 5:**
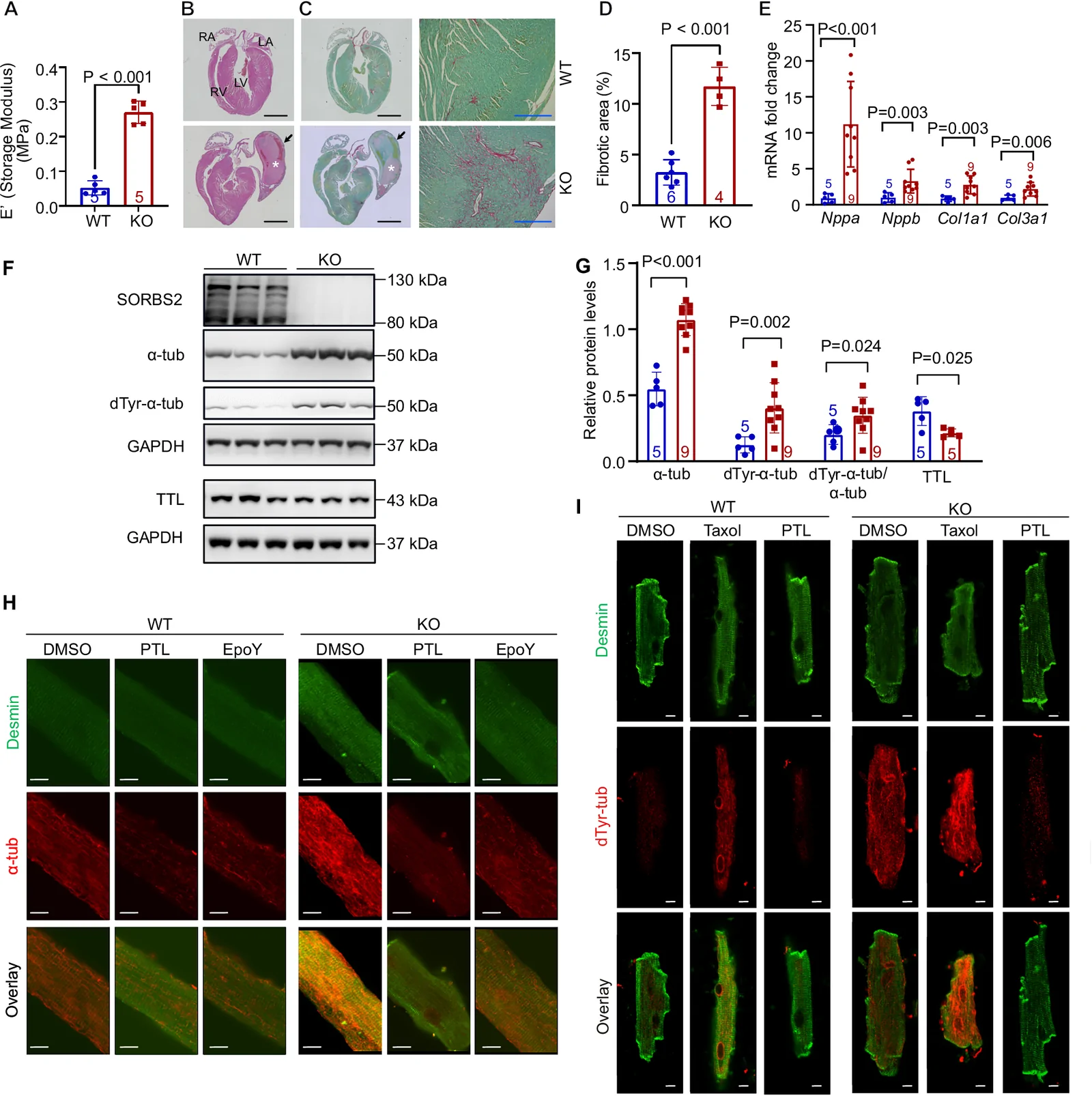
Mechanisms of diastolic dysfunction in *Sorbs2* KO. **A** Storage modulus of KO and WT ventricular myocardium, measured by dynamic mechanical analysis *t-*test. H&E (**B**) and Picrosirius red/fast green (**C**) stained sections of KO and WT hearts. The magnified image is shown to the right. Note massively dilated KO left atrium (arrow) containing organized thrombus (asterisk), and myocardial fibrosis (C, red staining). Black bar = 2 mm. Blue bar = 400 µm. **D** Quantification of myocardial fibrosis based on Picrosirius red/fast green stain. Each point represents a separate heart *t-*test. **E** RT-qPCR measurement of altered gene expression in KO vs WT *t-*test. **F** Immunoblots of KO and WT heart lysates. **G** Quantification of relative protein expression *t-*test. **H–I** Representative confocal images of immunostained, dissociated cardiomyocytes. Cardiomyocytes were treated for two hours with α-tubulin detyrosination inhibitors parthenolide (PTL) or EpoY, or microtubule-stabilizing agent taxol. Scale bar = 10 µm. Graphs show mean ± SD.

Active and passive factors can impair myocardial relaxation. We assessed passive factors by measuring the storage modulus of KO and WT hearts using dynamic mechanical analysis. This assay revealed that KO hearts had a 5.5-fold higher storage modulus (*P* < 0.001; Fig. 5A), indicating greater total passive myocardial stiffness.

Extracellular and cellular sources contribute to myocardial stiffness. We assessed the KO myocardium for fibrotic changes in the extracellular matrix by performing picrosirius red-fast green staining of 6-month-old myocardial sections (Fig. 5B, C). KO myocardium exhibited greater fibrosis (red staining, Fig. 5C). Quantification of fibrotic area demonstrated that it was significantly higher in KO than WT (Fig. 5D). Cardiac fibrotic markers *Col1a1* and *Col3a1* were upregulated in KO compared to WT, as were cardiac stress markers *Nppa* and *Nppb* (Fig. 5E).

The splicing of the sarcomeric protein titin is an important determinant of the passive stiffness of sarcomeres^21^. RT-PCR showed that the ratio of compliant (N2BA) to stiff (N2B) titin isoforms did not significantly differ between KO and WT (Supplementary Fig. 6), suggesting that altered titin splicing is not a major contributor to diastolic dysfunction in *Sorbs2-*deficient hearts.

We also assessed cellular changes that could contribute to myocardial stiffness. In addition to the aforementioned abnormalities in focal adhesion function (Fig. 4E-J), we found that loss of SORBS2 altered the cardiomyocyte microtubule network, an important determinant of cardiomyocyte stiffness^16^. Immunoblotting demonstrated greater α-tubulin and detyrosinated α-tubulin in KO myocardium, with an elevated ratio of detyrosinated to total tubulin (Fig. 5F, G). Consistent with greater tubulin detyrosination, tubulin tyrosine ligase (TTL), which catalyzes the post-translational addition of tyrosine to the C-terminal end of α-tubulin, was downregulated in KO myocardium (Fig. 5F, G).

These changes in α-tubulin level and tyrosination would stabilize microtubules and increase myocardial stiffness. To further test this hypothesis, we isolated cardiomyocytes from 6-month-old WT and KO mice and stained for α-tubulin (Fig. 5H) or detyrosinated α-tubulin (Fig. 5I). In keeping with the immunoblot data, vehicle-treated KO cardiomyocytes had stronger α-tubulin and detyrosinated α-tubulin staining than WT (Fig. 5H, I). As a positive control for detyrosinated α-tubulin staining, we treated cells with taxol, which stabilizes microtubules and increases detyrosinated α-tubulin. Cardiomyocyte treatment for 2 h with parthenolide (PTL) or EpoY, inhibitors of the carboxypeptidase that detyrosinates α-tubulin, reduced KO α-tubulin staining to levels comparable to WT (Fig. 5H). PTL similarly reduced detyrosinated α-tubulin signal in KO cardiomyocytes to WT levels (Fig. 5I). Together, these data indicate that *Sorbs2* ablation increases microtubules and microtubule detyrosination.

**Fig. 6:**
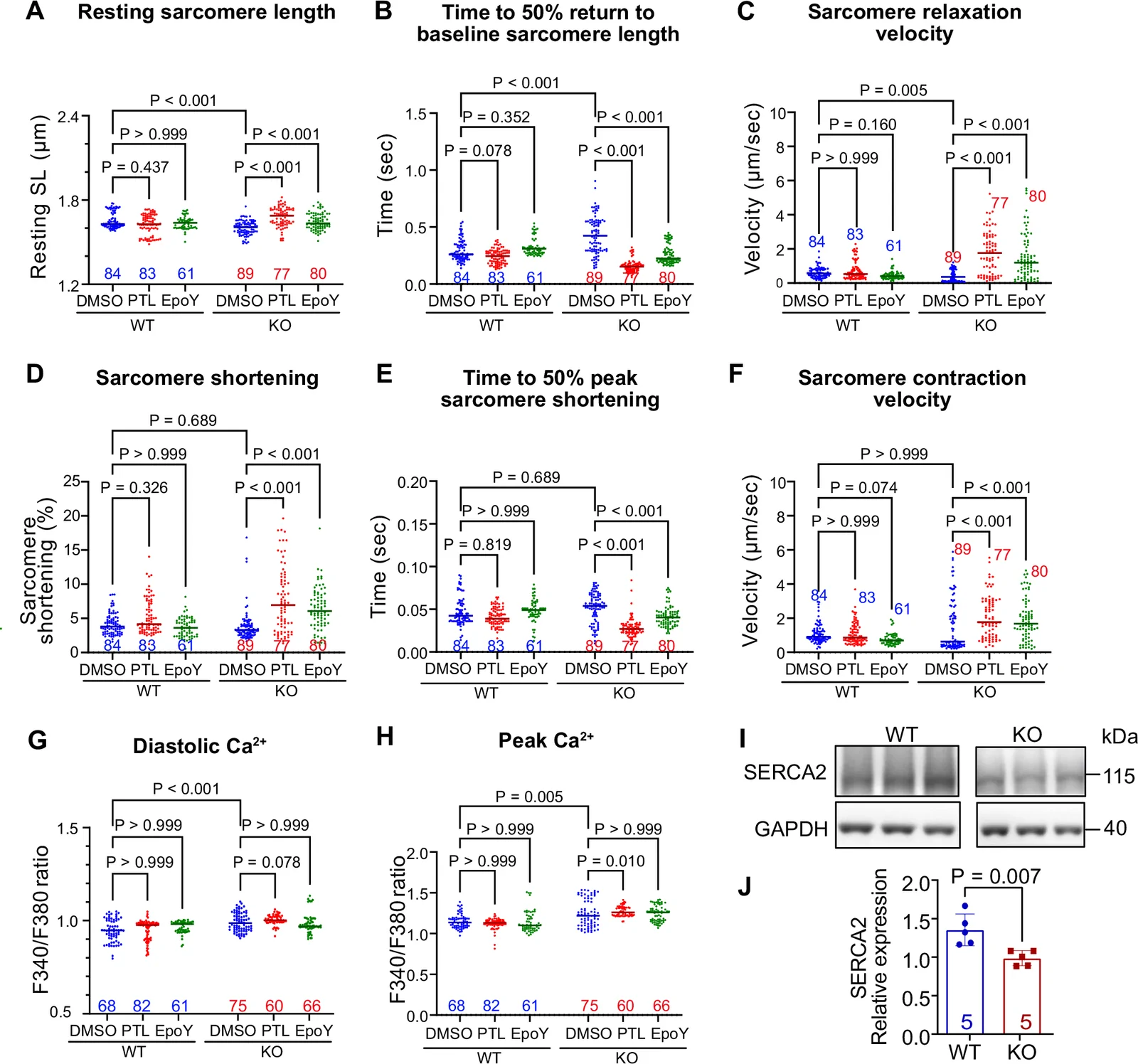
Impaired relaxation and Ca^2+^ handling of isolated *Sorbs2* KO cardiomyocytes. Sarcomere relaxation (**A**–**C**) and contraction (**D**–**F**) parameters for dissociated KO and WT cardiomyocytes, treated with vehicle (DMSO), PTL, or EpoY for 2 h prior to analysis using an IonOptix system. Each point represents one cell. Kruskal-Wallis with Dunn’s multiple comparison test. Cardiomyocytes were loaded with Fura-2 to ratiometrically record diastolic (**G**) and peak Ca^2+^ (**H**) levels. Each point represents one cell. Kruskal-Wallis with Dunn’s multiple comparison test. **I–J** Western blots of KO and WT heart lysates for SERCA2. Protein levels were quantified relative to GAPDH *t-*test. **A–H** Line indicates the median value. **J** Graph shows mean ± SD.

We investigated whether microtubule dysregulation contributes to diastolic dysfunction in KO cardiomyocytes by measuring sarcomere and cytosolic Ca^2+^ dynamics in dissociated cardiomyocytes treated with PTL, EpoY, or vehicle (DMSO). Vehicle-treated KO sarcomeres displayed impaired relaxation, reflected in reduced resting length (Fig. 6A), longer relaxation time, and slower relaxation velocity (Fig. 6B, C). In contrast, systolic parameters were largely similar between KO and WT, with sarcomere shortening, shortening time, and shortening velocity being comparable between genotypes (Fig. 6D–F). PTL and EpoY, inhibitors of α-tubulin detyrosination, did not significantly affect these parameters in WT cardiomyocytes (Fig. 6A–F). In contrast, PTL or EpoY enhanced the diastolic function of KO cardiomyocytes, as reflected by increased sarcomere length, sarcomere relaxation time, and sarcomere relaxation velocity (Fig. 6A–C). PTL and EpoY also increased systolic parameters of KO cardiomyocytes, enhancing sarcomere shortening, sarcomere shortening time, sarcomere contraction velocity, and Ca^2+^ release velocity (Fig. 6D–F). Together, these data support a mechanistic role for microtubule detyrosination in impaired cardiomyocyte relaxation.

Cardiomyocyte Ca^2+^ handling affects active cellular contributions to myocardial stiffness. To measure the effect of *Sorbs2* ablation on cardiomyocyte Ca^2+^ handling, we dissociated cardiomyocytes from 6-month-old WT and KO mice, loaded them with Fura-2, a ratiometric Ca^2+^-sensitive dye, and recorded Ca^2+^ transients under field pacing at 0.5 Hz. Compared to WT, KO cardiomyocytes had impaired clearance of cytoplasmic Ca^2+^ during diastole, as shown by elevated diastolic Ca^2+^ concentration (Fig. 6G), increased Ca^2+^ transient time to 50% baseline, and decreased Ca^2+^ decay velocity (Supplementary Fig. 7A, B). PTL and EpoY improved diastolic Ca^2+^ clearance, reducing the Ca^2+^ transient time to 50% baseline and enhancing Ca^2+^ decay velocity (Supplementary Fig. 7A, B). However, these agents did not normalize diastolic Ca^2+^ concentration (Fig. 6G). In systole, KO cardiomyocytes had elevated peak Ca^2+^ (Fig. 6H) without significantly altered Ca^2+^ release kinetics. PTL and EpoY did not significantly normalize peak Ca^2+^ (Fig. 6H) or the time to 50% Ca^2+^ release (Supplementary Fig. 7C), but they significantly increased Ca^2+^ release velocity (Supplementary Fig. 7D).

Ca^2+^ is cleared from the cytosol into the sarcoplasmic reticulum by SERCA2, which is inhibited by phospholamban (PLN). PLN phosphorylation alleviates this SERCA2 inhibition. SERCA2 levels were significantly lower in KO (Fig. 6I–J), while total PLN and its phosphorylation at Ser16 were comparable between KO and WT (Supplementary Fig. 7E, F). The change in SERCA2 would reduce the cytosolic Ca^2+^ clearance rate, consistent with the Ca^2+^ transient data. Elevated cardiomyocyte diastolic Ca^2+^ and lower rate of Ca^2+^ clearance would increase sarcomere cross-bridge activity, thereby impairing cardiomyocyte relaxation.

*Sorbs2* knockout has been reported to disrupt intercalated disk organization^9^, although this finding was not reproduced by McLendon et al.^8^. We examined intercalated disks in KO hearts and did not observe aberrant N-cadherin or Cx43 localization (Supplementary Fig. 7G, H), suggesting that altered organization of intercalated disks does not substantially contribute to the diastolic dysfunction of *Sorbs2* KO mice.

Together, our data indicate that loss of SORBS2 causes diastolic dysfunction through a combination of increased fibrosis and passive myocardial stiffness, increased microtubule stability and detyrosination, and impaired diastolic Ca^2+^ clearance.

### Altered gene expression downstream of SORBS2 deficiency

**Fig. 7:**
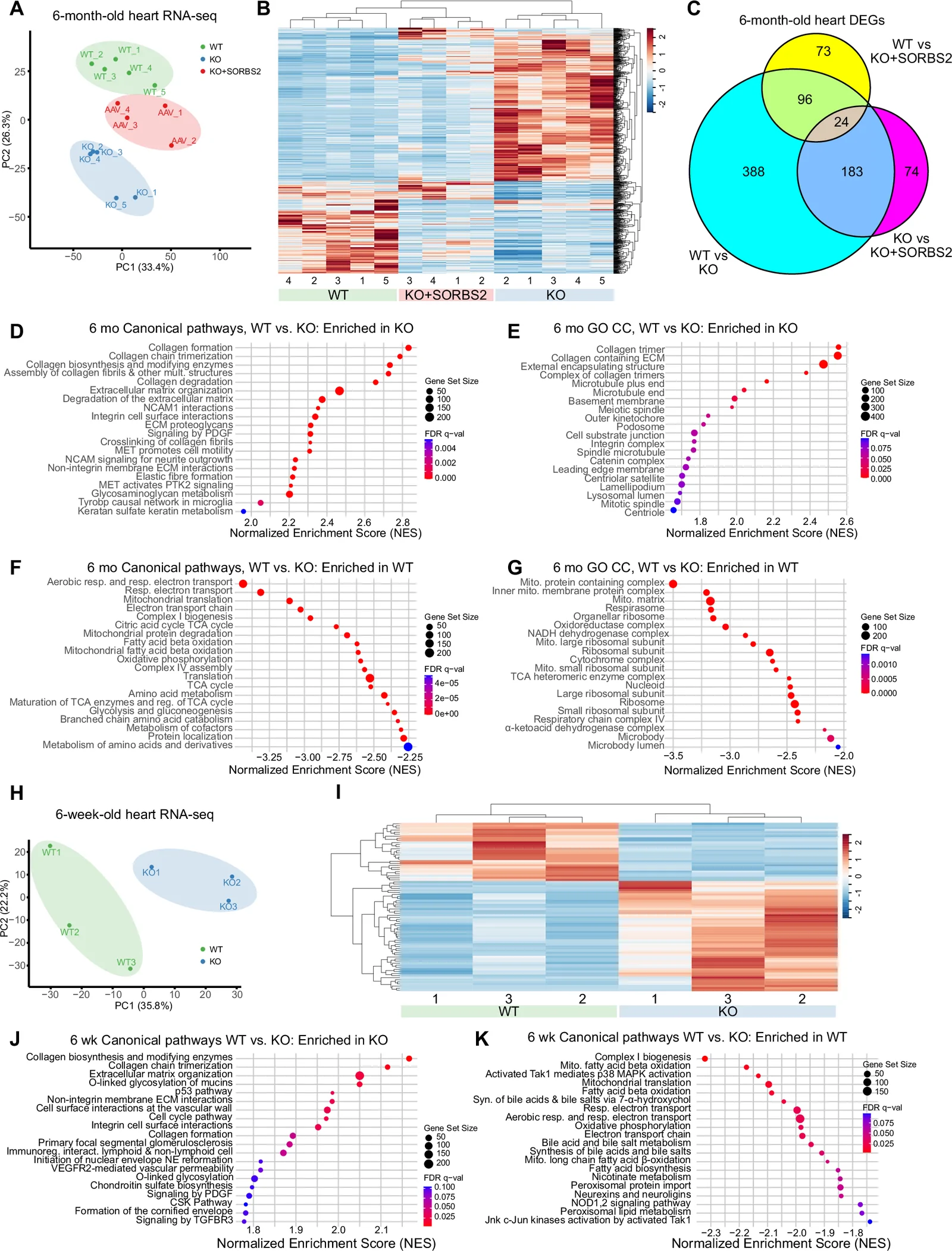
Changes in gene expression in *Sorbs2* KO hearts. **A–G** Bulk RNA-seq was performed on 6-month-old ventricular myocardium from WT, KO, and KO treated neonatally with AAV-Tnnt2-SORBS2 (KO + SORBS2). **A** Principal component analysis (PCA). **B** Hierarchical clustering of differentially expressed genes (DEGs; defined as genes with |log_2_FC | ≥ 1 and FDR ≤ 0.05, as determined by DESeq2). **C** Comparison of DEGs between KO and WT, KO and KO + SORBS2, and WT and KO + SORBS2. Gene ontology (GO) canonical pathways (**D**) and cellular components (CC; **E**) upregulated in KO (compared to WT). GO canonical pathways (**F**) and cellular components (CC; **G**) upregulated in WT (compared to KO). **H–K** Bulk RNA-seq was performed on 6-week-old WT and KO ventricular myocardium. **H** PCA plot. **I** Hierarchical clustering of DEGs (defined as genes with |log_2_FC | ≥ 1 and FDR ≤ 0.05, as determined by DESeq2). GO canonical pathways enriched for genes upregulated in KO (**J**) or WT (**K**).

To gain additional insights into the mechanisms underlying altered cardiac function in *Sorbs2* deficiency, we performed bulk RNA sequencing on 6-month-old ventricular myocardium from WT, KO, and KO mice treated with AAV9-Tnnt2-SORBS2 (KO + SORBS2). PCA analysis showed that independent biological replicates grouped together and were well-separated by between-group differences (Fig. 7A; Supplementary Data 3). A majority of differentially expressed genes (DEGs; FDR ≤ 0.05 and |fold-change | ≥ 2) between WT and KO were rescued by AAV-SORBS2 treatment (Fig. 7B, C).

We performed gene set enrichment analysis to identify pathways or cell components that were significantly impacted by SORBS2 deficiency. Pathways enriched in KO compared to WT included those related to collagen formation, extracellular matrix organization, cell motility, and integrin cell surface interactions, consistent with SORBS2’s function in the integrin-focal adhesion complex and our observation of increased fibrosis in KO hearts (Fig. 7D). Many of these differentially expressed genes were normalized by AAV-SORBS2 treatment (Fig. 7B and Supplementary Fig. 8A). Cell components enriched in KO included collagen fibrils, extracellular matrix, and microtubules (Fig. 7E and Supplementary Fig. 8B). Transcript levels of *Tuba1a* (encoding α-tubulin), *Ttl*, and *Vash1* (encoding the primary tubulin detyrosinase) were not significantly altered in *Sorbs2* KO, suggesting that the protein changes that we detected occurred post-transcriptionally.

Pathways enriched in WT (i.e., downregulated in KO) were predominantly related to the TCA cycle, oxidative phosphorylation, and mitochondria (Fig. 7F), and many of these DEGs were corrected by AAV-Tnnt2-SORBS2 (Supplementary Fig. 8C). Similarly, cell components significantly enriched in WT and restored by AAV-Tnnt2-SORBS2 were related to mitochondria and the respirasome (Fig. 7G and Supplementary Fig. 8D).

To identify gene expression changes that occur early in the disease process, we performed bulk RNA sequencing on 6-week-old ventricular WT and KO myocardium. PCA analysis confirmed high intergroup and low intragroup variation (Fig. 7H). We identified 28 and 53 genes more highly expressed in WT or KO, respectively (P_adj_< 0.05 and | Log_2_FC | ≥ 1; Fig. 7I; Supplementary Data 3). Canonical pathways enriched in KO continued to include collagen biosynthesis, extracellular matrix organization, and integrin cell surface interactions, and canonical pathways enriched in WT included mitochondrial fatty acid β-oxidation, respiratory electron transport, and oxidative phosphorylation.

These data identify integrin signaling, collagen formation, and extracellular matrix organization as processes enriched in KO, and mitochondria and respiratory electron transport as processes enriched in WT. Downregulation of gene programs related to mitochondria and oxidative phosphorylation by SORBS2 inactivation is consistent with prior studies linking focal adhesion kinase signaling to mitochondrial function^22,23^.

## Discussion

Our study identifies SORBS2 as an essential regulator of diastolic function. Using a global *Sorbs2* knockout mouse model, we demonstrate that, in the first 6 months of life, loss of SORBS2 leads to progressive diastolic dysfunction with preserved systolic performance, accompanied by atrial remodeling and reduced survival. Mechanistically, SORBS2 deficiency disrupts the integrin-adhesome complex, drives ECM remodeling, alters microtubule dynamics, and impairs calcium handling. Importantly, cardiomyocyte-directed re-expression of SORBS2 was sufficient to substantially rescue these phenotypes, establishing SORBS2 as a cardiomyocyte-intrinsic regulator of diastolic homeostasis.

In cardiomyocytes, SORBS2 is enriched at intercalated discs, cardiac dyads, and focal adhesions, functioning as a cytoskeletal adaptor that links mechanical cues to signaling pathways^24^. Furthermore, our study highlights the function of SORBS2 and the adhesome in regulating cardiac relaxation. Although several groups have studied *Sorbs2* global or cardiac-specific knockout mice, and one previously noted diastolic dysfunction^8^, the finding that impaired relaxation with preserved systolic function is the predominant cardiac phenotype of these mice through 6 months of age was not previously emphasized. Rather, prior studies focused on impaired systolic function, which becomes severe by 1 year of age^8^. Although one group reported intercalated disc disorganization and severe downregulation of Cx43^9^, this was not reproduced by another group^8^ or in this study.

ECM remodeling is a hallmark of diastolic dysfunction and contributes to increased myocardial stiffness and impaired relaxation^25^. In *Sorbs2*-deficient hearts, we observed substantial interstitial fibrosis, upregulation of ECM-associated genes, and activation of integrin signaling pathways, consistent with prior studies of SORBS2 deficient hearts^7,9^. Proximity proteomics, co-immunoprecipitation, and immunostaining further confirmed SORBS2’s enrichment at focal adhesion complexes, supporting its role in maintaining adhesome integrity.

Transcriptomic profiling of *Sorbs2*-ablated hearts revealed concerted upregulation of integrin and ECM-related transcripts, potentially to compensate for impaired signaling caused by loss of SORBS2. These data support a model in which cardiomyocyte-intrinsic loss of SORBS2 destabilizes focal adhesion signaling, leading to increased cytoskeletal stiffness and impaired mechanotransduction. These stress responses are well-established triggers of pro-fibrotic paracrine signaling from cardiomyocytes^26^, thereby activating cardiac fibroblasts and driving maladaptive ECM deposition. Thus, *Sorbs2* ablation disrupts ECM–integrin signaling through focal adhesions and promotes secondary matrix remodeling, consistent with recent work identifying SORBS2 as a GATA4-regulated, stress-inducible adaptor that integrates integrin-mediated mechanotransduction with cytoskeletal and ECM homeostasis^7^.

Diastolic relaxation requires rapid cytosolic Ca^2+^ clearance, primarily via sarcoplasmic reticulum (SR) reuptake by SERCA2. In *Sorbs2* KO cardiomyocytes, we observed elevated diastolic Ca^2+^, reduced transient amplitude, and delayed decay kinetics—hallmarks of impaired SR calcium cycling. These changes were accompanied by reduced SERCA2 expression in the absence of alterations in total or Ser16-phosphorylated PLN, suggesting a PLN-independent mechanism. SERCA2 downregulation in *Sorbs2* KO hearts is consistent with prior reports in a *Sorbs2* cardiac-specific knockout model^8^, although our study reveals earlier onset and greater functional impairment.

Notably, SERCA2 mRNA levels were preserved, suggesting post-transcriptional mechanisms underlying reduced SERCA2 protein abundance. We observed increased detyrosinated α-tubulin, a modification that stiffens microtubules^27,28^ and may impairs SR vesicle trafficking^29^, suggesting a potential mechanism underlying reduced protein level. Increased detyrosinated α-tubulin and destabilization of dyadic architecture, including reduced JPH2 abundance and impaired T-tubule regularity, provide a mechanistic link between cytoskeletal remodeling and disrupted calcium microdomain organization. These findings support an indirect role for SORBS2 in diastolic calcium homeostasis via maintenance of cytoskeletal and dyadic integrity.

Cytoskeletal remodeling, particularly microtubule dynamics, has emerged as a key contributor to myocardial stiffness and impaired diastolic relaxation^16,27,30^. In *Sorbs2* KO hearts, we observed upregulation of both α-tubulin and detyrosinated α-tubulin, coupled with downregulation of TTL, consistent with excessive microtubule stabilization.

Functionally, detyrosinated microtubules are more resistant to depolymerization and promote mechanical coupling between sarcomeres and the sarcolemma, increasing passive stiffness and delaying relaxation^27,30^. Pharmacological inhibition of tubulin detyrosination using PTL or EpoY restored sarcomere relaxation dynamics in *Sorbs2* KO cardiomyocytes, directly linking microtubule dysregulation to diastolic dysfunction. Together, our results suggest that SORBS2 antagonizes microtubule detyrosination, likely indirectly through impaired mechanotransduction signaling at focal adhesions^31,32^.

ECM remodeling and destabilization of the adhesome have been implicated in mitochondrial dysfunction and diastolic impairment. Focal adhesion kinase (FAK), a core adhesome component, mediates integrin-dependent mitochondrial biogenesis and bioenergetics in cardiomyocytes^22^. Activation of integrin–FAK signaling enhances mitochondrial respiration via STAT3, increasing ATP production and reducing reactive oxygen species^33^. Conversely, ECM stiffening or altered integrin engagement disrupts microtubule-dependent mitochondrial positioning and dynamics, impairing ATP delivery and calcium responsiveness^34^. Furthermore, focal adhesions and associated protein complexes stabilize microtubules^31,32^, and stable, long-lived microtubules preferentially accumulate tubulin detyrosination^28^, thereby increasing cardiomyocyte stiffness^27,30^.

In *Sorbs2* KO hearts, transcriptomic downregulation of oxidative phosphorylation and tricarboxylic acid (TCA) cycle genes was accompanied by excessive detyrosinated microtubules. This combination likely disrupts mitochondrial trafficking and calcium integration at dyadic–mitochondrial junctions, compromising energy supply and promoting diastolic dysfunction. Restoration of SORBS2 expression normalized both mitochondrial gene expression and diastolic performance, supporting a model wherein adhesome–cytoskeleton–mitochondria interplay is essential for efficient myocardial relaxation.

Collectively, our findings define a previously unrecognized role for SORBS2 as a cardiomyocyte-intrinsic determinant of myocardial diastolic function and reveal the adhesome as a critical regulator of cardiomyocyte relaxation. While prior studies have implicated SORBS2 in diverse aspects of cardiomyocyte structure and stress responses, our work provides integrative evidence that SORBS2 governs myocardial relaxation by coupling focal adhesion integrity to cytoskeletal dynamics and calcium microdomain organization through an adhesome–cytoskeleton–calcium signaling axis.

From a translational perspective, although SORBS2 itself is unlikely to represent a direct therapeutic target due to its foundational structural role, its loss exposes downstream, more selectively targetable mechanisms—particularly microtubule post-translational modifications and cytoskeletal–calcium coupling—that directly regulate myocardial stiffness and relaxation. Disease-associated upregulation of SORBS2 in human cardiomyopathy likely reflects a compensatory response to cytoskeletal and microdomain stress; defining when and why this adaptive response fails may reveal new strategies to preserve diastolic homeostasis across diverse cardiac diseases.

These findings should be interpreted in the context of several experimental considerations. This study employed a global *Sorbs2* knockout model. Cardiomyocyte-directed AAV re-expression substantially rescued diastolic dysfunction and remodeling, supporting its requirement in postnatal cardiomyocytes to maintain cardiomyocyte relaxation, but more refined conclusions about SORBS2’s cell-autonomous function will benefit from conditional gene inactivation strategies. The late death of AAV-Tnnt2-SORBS2-treated KO mice might be due to insufficient AAV-directed SORBS2 expression, or to SORBS2 requirement in development or in other cell types. While ventricular diastolic dysfunction precedes atrial enlargement in the *Sorbs2* KO model, suggesting that atrial remodeling is secondary to diastolic dysfunction, a direct atrial cardiomyocyte role of SORBS2 cannot be fully excluded. While our study clearly demonstrates the essential role of SORBS2 in maintaining cardiomyocyte diastolic function in mice and SORBS2 dysregulation in human heart disease, further studies are required to elucidate SORBS2’s contribution to the pathophysiology of human heart disease, such as HFpEF.

## Methods

### Animals

All animal experiments were performed under protocols approved by the Boston Children’s Hospital Institutional Animal Care and Use Committee. We have complied with all relevant ethical regulations for animal use. Mice with a constitutive *Sorbs2* knockout allele *(Sorbs2e8*; also known as C57BL/6N-A^*tm1Brd*^ *Sorbs2*^*tm1.1(KOMP)Mbp*^/JMmucd) were obtained from the Mutant Mouse Resource & Research Center: UC DAVIS (stock number: 049805-UCD). Mice were maintained in a specific pathogen-free facility under a 12 h/12 h light/dark cycle at 22 ± 2 °C and 45–65% humidity. Genotyping was performed using PCR primers listed in Supplementary Table 7. Mice were euthanized in an uncharged chamber by gradual-fill CO_2_ asphyxiation as specified by the American Veterinary Medical Association’s Guidelines for the Euthanasia of Animals.

### Echocardiography

Mice were anesthetized with 3% isoflurane and lack of hind paw withdrawal response was confirmed. Transthoracic echocardiography was performed using a VisualSonics Vevo 3100 system with MX550D transducer (Visual Sonics Inc) under 1.0–1.5% isoflurane anesthesia, titrated to maintain heart rate at 400–450 bpm. Body temperature was controlled throughout.

Left ventricular (LV) systolic function was assessed by M-mode in the mid-ventricular short axis view at the level of the papillary muscles. Measurements included LV end-diastolic diameter (LVID, d), LV end-systolic diameter (LVID, s), LV fractional shortening (LVFS), LV ejection fraction (LVEF), end-diastolic interventricular septal wall thickness (IVS, d), and LV posterior wall thickness (LVPW, d). Diastolic function was evaluated in the apical 4-chamber view using pulsed-wave and tissue Doppler at the mitral valve. Parameters included peak early (E) and late (A) diastolic mitral inflow velocities, and early diastolic mitral annular velocity (E’). All measurements represent the average of ≥3 cardiac cycles. Mice recovered uneventfully from anesthesia.

### Pressure-volume analysis

Pressure-volume loop analysis was performed as previously described^35^. Mice were anesthetized with 3% isoflurane, then maintained at 1.0–1.5% isoflurane. A 1.4-F catheter with pressure and conductance sensors (Transonic ADV500 Scisense Pressure-Volume Measurement System) was advanced into the LV via an apical approach. To obtain load-independent indices, the inferior vena cava was transiently occluded during a pause in mechanical ventilation. Data were analyzed using PV Lab Chart software (version 8).

### AAV production and injection

The full-length *Sorbs2* cDNA was previously described^36^. We excluded the neuron-specific exon (NSE), which is predominantly expressed in the nervous system. Truncated versions of the *Sorbs2* cDNA were cloned into a plasmid with an N-terminal 3× haemagglutinin (HA) epitope tag. The complete sequence is provided in Supplementary Table 3.

For in vivo overexpression experiments, genes of interest were subcloned into an AAV9-cardiac troponin T promoter vector (Addgene, 69915)^37^. AAV was produced by co-transfecting HEK293 T cells with the AAV plasmids and helper plasmids AAV9-Rep/Cap (Addgene, 112865) and pAd-ΔF6 (Addgene, 112867) using polyethylenimine (Polysciences, 23966-2). Viruses were purified by iodixanol density gradient ultracentrifugation as described previously^37^. AAV was titered by qPCR and injected subcutaneously into postnatal day 2 (P2) pups at a dose of 5.5 × 10^10^ vg/g in a total volume of less than 30 μl.

### RNA extraction and quantitation

Mouse tissues were homogenized by adding 5 mm Stainless Steel Beads (Qiagen, 69989) to tissues and processing in a TissueLyser (Qiagen) according to the manufacturer’s recommendations, using 25 Hz for 2 min for complete tissue rupture. Total RNA was isolated using Direct-zol RNA Miniprep Plus kits (Zymo Research, R2071). DNase I-pretreated RNA (1 µg) was reverse transcribed using SuperScript III First-strand Synthesis SuperMix (Thermo Fisher, 18080400). Gene expression was analyzed by qPCR using Power SYBR Green PCR Master Mix (Applied Biosystems, 4367659) and a Bio-Rad CFX96 or CFX384 touch thermocycler. qPCR primers are listed in Supplementary Table 7.

### Titin isoform analysis

Titin isoforms were analyzed using a semi-quantitative PCR approach as previously described^38^. Briefly, PCR was performed using Taqman polymerase (Takara) with primer sequences provided in Supplementary Table 7. The resulting PCR products were separated and quantified using a Bioanalyzer 2100 Expert instrument (Agilent) with the DNA1000 kit.

### RNA sequencing

For RNAseq, total RNA was isolated using Direct-zol RNA Miniprep Plus kits (Zymo Research, R2071). RNA integrity and concentration were assessed using an Agilent TapeStation 4200 (Agilent Technologies, Palo Alto, CA, USA), with all samples having RIN values > 9. Poly(A) RNA purification, stranded RNA-seq library preparation (Illumina Stranded mRNA kit), and 150 nt paired-end sequencing were performed by MedGenome on an Illumina NovaSeq 6000 S4 sequencer. Raw reads were trimmed using Trim Galore (https://github.com/FelixKrueger/TrimGalore) and aligned to the mouse reference genome GRCm38/mm10 using STAR (version 2.7.10)^39^. A minimum of 40 million paired-end reads were obtained per sample, with at least 40 million mapped paired-end reads per sample. Gene expression values were calculated as fragments per kilobase million (FPKM) using Cufflinks (version 2.1.1)^40^. Principal component analysis was performed using the “prcomp” function in R. Differential expression analysis was performed using the nf-core/differential abundance pipeline (v1.5.0), which employs DESeq2 (Wald test, parametric fit)^41^. Normalization was carried out using the ratio-based size factor estimation, and log fold changes were shrinkage-adjusted. Multiple testing correction was performed using the Benjamini–Hochberg method. Genes were considered significantly differentially expressed if they exhibited an absolute log_2_ fold change ≥1 (fold change ≥2) and an adjusted *p* value (FDR) ≤ 0.05. The RNA-seq results are summarized in Supplementary Data 3.

### BioID and mass spectrometry

BioID proximity proteomics was performed as previously reported^18^. AAV-BirA*-*Sorbs2* was constructed by fusing BirA* to the 5’ end of the *Sorbs2* open reading frame. In total, 0.5 mg of protein extract was immunoprecipitated using streptavidin Dynabeads (Invitrogen, M280). Beads were washed five times with RIPA lysis buffer (Santa Cruz, #sc-24948) and stored in PBS for on-bead digestion. Streptavidin pull-downs were performed using hearts from four control mice injected with AAV-GFP and six mice injected with AAV-BirA*-SORBS2. Beads were subsequently washed at least five times with 100 µL of 50 mM ammonium bicarbonate. Digestion was initiated by adding 5 µL of modified sequencing-grade trypsin (200 ng/µL; Promega, Madison, WI), followed by overnight incubation at 37 °C. Beads were then removed magnetically, and the supernatant was dried using a speed-vac. Samples were resuspended in 50 µL of HPLC solvent A (2.5% acetonitrile, 0.1% formic acid) and desalted using STAGE tips. On the day of analysis, samples were reconstituted in 10 µL of solvent A.

Nano-scale reverse-phase HPLC columns were prepared by packing 2.6 µm C18 silica beads into a fused silica capillary (100 µm inner diameter × ~30 cm length) with a flame-drawn tip. After equilibration, samples were loaded using a Famos autosampler (LC Packings, San Francisco, CA), and peptides were eluted via a gradient of increasing concentrations of solvent B (97.5% acetonitrile, 0.1% formic acid). Eluted peptides underwent electrospray ionization and were analyzed using an LTQ Orbitrap Velos Elite ion-trap mass spectrometer (Thermo Fisher Scientific, Waltham, MA). Peptides were detected, isolated, and fragmented to generate tandem mass spectra, which were matched to protein databases using Sequest (Thermo Fisher Scientific, Waltham, MA). Databases included reversed sequences for false discovery rate (FDR) estimation, and results were filtered to achieve a peptide FDR of 1–2%.

### Western blotting

Protein samples (50 µg) were separated by 4–12% SDS–PAGE (Invitrogen, NW04120BOX) and transferred to Immobilon-P PVDF membranes (Merck Millipore, IPVH00010). Membranes were blocked with 5% non-fat dry milk, incubated with primary antibodies (Supplementary Table 8) overnight at 4 °C, and then with secondary antibodies for 1 h at room temperature. Signals were detected using a Fujifilm LAS-3000 imager and quantified with Fiji software. Uncropped gel images are shown in Supplementary Data 4.

Capillary-based immunoassay was performed using the Wes-Simple Western method with the anti-rabbit detection module (ProteinSimple). Protein expression was measured by chemiluminescence and quantified as area under the curve using the Compass for Simple Western program (ProteinSimple).

### Co-immunoprecipitation assays

For each co-immunoprecipitation, 50 µL protein G beads (Invitrogen, 10003D) and 5 µL primary antibody (1 mg/mL) or IgG control were incubated with 500 µg tissue lysate overnight at 4 °C. Beads were washed ten times with immunoprecipitation buffer (Thermo Fisher, 87787) containing protease inhibitor (Thermo Fisher, A32965). Bound proteins were eluted by boiling beads at 100 °C for 10 min.

### Histology

To measure fibrosis, mouse hearts were excised and fixed in 4% PFA overnight, dehydrated through an ethanol gradient, embedded in paraffin, and sectioned at 7 µm. Sections were dewaxed, rehydrated, and stained with hematoxylin and eosin, or Fast green/ Picrosirius red. Sections were imaged by a widefield microscope (Keyence) at ×10 and ×20 magnification.

### Immunofluorescence assays

Mouse hearts were fixed overnight in 4% paraformaldehyde (PFA), cryoprotected in 30% sucrose, and embedded in tissue freezing medium. Cryosections (10 µm) were permeabilized with 0.5% Triton X-100 for 20 min and blocked with 10% normal goat serum for 1 h. Isolated cells were fixed in 4% PFA for 15 min, washed with PBS, permeabilized with 0.1% Triton X-100 for 10 min, and blocked with 1% BSA for 1 h.

Sections or cells were incubated with primary antibodies (Supplementary Table 8) overnight at 4°C, followed by Alexa Fluor-conjugated secondary antibodies (Invitrogen, 1:200) for 1 h at room temperature. Nuclei were stained with DAPI (1 µg/mL) for 10 min. Images were acquired using a confocal microscope with the following excitation/emission settings: DAPI (405/420-470 nm), Alexa Fluor 488 (488/490-520 nm), Alexa Fluor 555 (543/560-620 nm), and Alexa Fluor 647 (647/ > 650 nm).

### Adult cardiomyocyte isolation and treatment

Adult mouse ventricular myocytes (AMVMs) were isolated enzymatically from mouse hearts^37^. Briefly, hearts were excised from isoflurane-anesthetized mice, rinsed in cold perfusion buffer (in mM: 137 NaCl, 15 glucose, 20 HEPES, 4.9 KCl, 1.2 MgCl_2_, 1.2 NaH_2_PO_4_, 5 taurine, and 10 BDM; pH 7.4), and mounted on a Langendorff perfusion system. Hearts were perfused at 37 °C with oxygenated Ca^2+^-free perfusion buffer until blood was cleared, followed by digestion with collagenase type II (0.7 mg/mL; Worthington, LS004176), protease type XIV (0.1 mg/mL; Sigma-Aldrich, P5147), BSA (1 mg/mL; Sigma-Aldrich, A3912), and 50 µM Ca^2+^. Softened ventricles were minced, agitated, and filtered through a 100 µm cell strainer. Cells were centrifuged at 20 g for 1 min and subjected to a Ca^2+^ gradient recovery (0.25, 0.35, 0.52, and 1 mM). Viable myocytes were enriched by gravity sedimentation for 5–7 min, and the supernatant containing dead cells was aspirated. Cells were resuspended in normal Tyrode’s (NT) solution containing 1 mM Ca²⁺ and maintained at 37 °C in NT solution for up to 6 h.

For pharmacological manipulation, AMVMs were incubated in NT solution with 10 μM Taxol (Cytoskeleton, TXD01), 10 μM parthenolide (Sigma-Aldrich, P0667), or 20 μM epothilone-Y (EpoY; Sigma-Aldrich, SML2301), each dissolved in dimethyl sulfoxide (DMSO), at 37 °C for 2 h^27,42^. DMSO alone served as the vehicle control. WT and KO myocytes from each condition were subsequently used for functional assays.

Sarcomere shortening and relaxation were measured in freshly isolated AMVMs using the IonOptix Calcium and Contractility System. Briefly, AMVMs were placed in a C-Stim CMC microscope chamber. The cells were electrically stimulated at 0.5 Hz using a field stimulator, and changes in sarcomeric length were recorded. Basal and peak sarcomere length, maximum departure/return velocities, time to peak 50%, and time to baseline 50% were measured. All measurements were performed at room temperature. All parameters were quantified using IonWizard software.

For calcium transient analysis, AMVMs were incubated with 1 µM Fura-2 AM (ThermoFisher, F14185) at 37 °C in 5% CO₂ for 20 min, followed by washout and stimulation at 0.5 Hz. Intracellular calcium dynamics were recorded using ratiometric photometry (340/380 nm excitation) via a photomultiplier tube, and parameters including diastolic Ca²⁺, peak Ca^2+^, transient amplitude, and decay kinetics were analyzed.

A subset of AMVMs was fixed for immunofluorescence analysis. AMVMs were plated on laminin-coated 96-well plates (5 µg/ml; Sigma-Aldrich, L2020) for 30 min and cultured in MEM (Sigma-Aldrich, M2279) supplemented with 10 mM BDM. Cells were then fixed in pre-chilled 100% methanol at –20 °C for 8 min. Following fixation, wells were rinsed with PBS and blocked with 5% BSA in PBS for 1 h at room temperature. Cells were incubated at 4 °C for 48 h with primary antibodies: anti-α-tubulin (1:100), anti-detyrosinated α-tubulin (1:100) and anti-desmin (1:200). After PBS washes, cells were incubated with Alexa Fluor–conjugated secondary antibodies (1:500) for 1 h at room temperature, counterstained with DAPI. Imaging was performed using Olympus confocal microscope under identical acquisition settings for all groups.

### Neonatal cardiomyocytes isolation and treatment

Neonatal mouse ventricular myocytes (NMVMs) were isolated from 1-to 2-day-old *Sorbs2* WT or KO pups using Neonatal Cardiomyocyte Isolation Kit (Miltenyi Biotec, 130-100-825) according to the manufacturer’s instructions. Cell preparations typically contained >95% cardiomyocytes. F-actin was stained using phalloidin staining. Images were acquired using the 60×objective on the Olympus microscope. All cells were measured as a unitary unit. Cell area and F-actin intensity were measured using ImageJ software. F-actin average length (in µm), the branches number and junctions were quantified using ImageJ Skeleton plugin.

### Myocardial mechanical analysis

To evaluate the comparative stiffness of WT and KO hearts, the tissues underwent dynamic mechanical analysis (DMA). The testing setup measures the tissues’ viscoelastic response in a dynamic environment, allowing for the differentiation between the elastic behavior (storage modulus) and the viscous behavior (loss modulus). When stressed, soft biological tissues both store elastic energy and dissipate energy^43^. This creates a time dependent stress response as a function of testing frequency. Because of this, dynamic testing can elucidate more consistent assessment of mechanical properties than traditional static tests^44^. Typical heart rates of mice are on the scale of ~600 beats/min (10 Hz)^45^, making the frequency dependent responses an important aspect when evaluating relative cardiac tissue stiffnesses under relevant mechanical stimuli.

The testing setup was designed and executed in accordance with ASTM D5024^46^. Samples were biopsied (4 mm diameter) from the left ventricular wall. Tissue thickness was measured with digital calipers (Mitutoyo, Kawasaki, Japan) prior to testing. Each tissue specimen was rehydrated with 37 °C phosphate buffered saline (PBS) prior to loading into the DMA (Mettler Toledo DMA 1, Columbus, OH, USA). The samples were compressed between two flat parallel plates (5-10 µm displacement) across a logarithmic frequency sweep covering 1–300 Hz. Storage moduli (E’) was recorded for each specimen across the full sweep. In all specimens, E’ plateaued in frequency domains below ~50 Hz. For each sample, an E’ value was established as the average across ~1–10 Hz in order to encapsulate elastic performance across low to physiological tissue properties. The plots provided indicate the average values with the error bars representing standard error of the mean.

### Statistics and reproducibility

Statistical analysis was performed using Prism 9 (GraphPad, San Diego, CA, USA). Normality was assessed using the Shapiro-Wilk test. For normally distributed data, group comparisons were conducted using an unpaired Student’s *t* test for two groups or one-way analysis of variance (ANOVA) followed by Dunnett’s or Sidak’s post hoc tests for multiple groups. Non-normally distributed data were analyzed with the Mann–Whitney U test for two-group comparisons or the Kruskal-Wallis test followed by Dunn’s multiple comparisons test for multiple groups. Statistical significance was set at *p* < 0.05. All experiments were performed with at least three biological replicates. Experimental animals were randomly assigned to each experimental/control group. Investigators were blinded to the genotypes of the individual animals during the experiments and outcome assessments.

### Reporting summary

Further information on research design is available in the Nature Portfolio Reporting Summary linked to this article.

## Supplementary information

Supplementary Information

Description of Additional Supplementary Materials

Supplementary Data 1

Supplementary Data 2

Supplementary Data 3

Supplementary Data 4

Supplementary Data 5

Reporting Summary

Transparent Peer Review file

## Data Availability

The sequencing data used in this study are available in the GEO database under accession code GSE306787. The mass spectrometry proteomics data have been deposited to the ProteomeXchange Consortium via the PRIDE partner repository with the dataset identifier PXD078837. Uncropped western blots are provided in Supplementary Data 4. Numerical source data underlying tables and figures is provided in Supplementary Data 5. All data and reagents used in this study can be obtained from the corresponding authors upon reasonable request.
