# Supplementary material for: SORBS2 regulates diastolic function through cytoskeletal networks and calcium handling
Source: Commun Biol. 2026 Jun 17;9:1291. doi: 10.1038/s42003-026-10503-6 (PMC13639047; doi:10.1038/s42003-026-10503-6)
Supplement: Supplementary file 1 [file 42003_2026_10503_MOESM1_ESM.pdf]

Supplementary Materials

Wu et al., SORBS2 regulates diastolic function through cytoskeletal networks and calcium handling.

Supplementary Tables:

Supplementary Table 1. Echocardiographic parameters at 6 months in WT and KO groups without AAV

Supplementary Table 2. Echocardiographic parameters at 3 to 4 months in WT and KO groups without AAV

Supplementary Table 3. Sequences of full-length and truncated *Sorbs2* cDNA constructs used in this study

Supplementary Table 4. Body weight, heart weight and lung weight at 6 months in WT and KO groups with AAV

Supplementary Table 5. Echocardiographic parameters at 6 months in WT and KO groups with different truncated *Sorbs2* constructs

Supplementary Table 6. Top proteins identified through proximity proteomics

Supplementary Table 7. Oligonucleotides used in this study

Supplementary Table 8. Antibodies used in this study

Supplementary Figures:

Supplementary Figure 1. SORBS2 expression and functional requirement for postnatal survival

Supplementary Figure 2. Functional analysis of *Sorbs2* KO and WT hearts

Supplementary Figure 3. Progressive cardiac remodeling in *Sorbs2* KO mice

Supplementary Figure 4. Effect of AAV9-SORBS2 on *Sorbs2* KO heart function

Supplementary Figure 5. Localization of BirA*-SORBS2, SORBS2, and JPH2

Supplementary Figure 6. RT-PCR analysis of titin isoform expression in *Sorbs2* KO and WT

Supplementary Figure 7. Characterization of *Sorbs2* KO cardiomyocytes

Supplementary Figure 8. Comparisons of gene expression between KO and KO+SORBS2 hearts

Supplementary data

Supplementary Data 1. Zip file of a 16-bit TIF z-stack of an isolated adult cardiomyocyte stained for SORBS2, JPH2, and nuclei. Open in ImageJ.

Supplementary Data 2. SORBS2 BioID proximity proteomics

Supplementary Data 3. Union of DEGs between Sorbs2 KO and control in 6w and 6m hearts.

Supplementary Data 4. Uncropped gels.

Supplementary Data 5. Numerical source data.

Supplementary Table 1. Echocardiographic parameters for 6-month-old WT and KO mice.

6 months	WT (n=8)	KO (n=10)	P value
HR (bpm)	430.7 (395.1, 462.1)	398.2 (370.6, 439.3)	0.203
LVEF (%)	54.5 (49.8, 57.9)	52.6 (47.7, 55.9)	0.408
LVFS (%)	27.8 (24.7, 30.1)	26.5 (23.6, 28.6)	0.360
LVID, s (mm)	2.8 (2.7, 3.1)	2.7 (2.6, 3.3)	0.633
LVID, d (mm)	3.9 (3.8, 4.3)	3.8 (3.6, 4.3)	0.633
IVS, d (mm)	0.8 (0.7, 0.9)	0.8 (0.7, 0.8)	0.633
LVPW, d (mm)	0.7 (0.7, 0.9)	0.9 (0.5, 1.1)	0.515
LV mass (mg)	88.8 (74.2, 103.1)	88.9 (59.2, 115.1)	0.829
LA (mm)	1.8 (1.5, 2.0)	2.3 (1.9, 2.8)	**0.016**
Peak mitral E velocity (mm/sec)	530.1 (492.6, 605.8)	910.4 (779.7, 1094.1)	**<0.001**
Peak mitral A velocity (mm/sec)	286.0 (252.3, 325.2)	330.2 (292.8, 420.1)	0.051
Mitral E/A	2.0 (1.8, 2.0)	3.0 (2.1, 3.4)	**0.004**
Peak mitral E' velocity (mm/sec)	-28.2 (-30.4, -21.1)	-20.4 (-22.5, -17.6)	**0.027**
Mitral E/E'	21.4 (17.8, 26.1)	43.2 (38.0, 60.2)	**<0.001**

Statistical comparisons between groups were performed using the Mann-Whitney *U* test. Data are presented as the median (interquartile range).

Supplementary Table 2. Echocardiographic parameters for 3-month-old WT and KO mice.

3 months	WT (n=10)	KO (n=9)	P value
HR (bpm)	447.1 (432.2, 457.3)	416.41 (403.2, 444.7)	0.065
LVEF (%)	52.7 (49.1, 62.4)	48.1 (43.7, 55.9)	0.095
LVFS (%)	26.8 (24.4, 33.1)	24.0 (21.4, 28.6)	0.079
LVID, s (mm)	3.0 (2.5, 3.1)	3.3 (2.6, 3.4)	0.211
LVID, d (mm)	4.0 (3.7, 4.1)	4.1 (3.6, 4.5)	0.604
IVS, d (mm)	0.8 (0.8, 0.9)	0.9 (0.7, 1.0)	0.661
LVPW, d (mm)	0.7 (0.6, 0.8)	0.7 (0.6, 1.0)	0.968
LV mass (mg)	89.3 (73.5, 96.0)	94.8 (79.4, 109.9)	0.497
LA (mm)	1.9 (1.8, 2.2)	2.0 (1.9, 2.4)	0.156
Peak mitral E velocity (mm/sec)	539.4 (470.2, 565.8)	736.4 (554.7, 909.1)	**0.028**
Peak mitral A velocity (mm/sec)	315.0 (185.5, 425.0)	283.6 (214.4, 370.4)	1.000
Mitral E/A	1.6 (1.4, 2.7)	2.4 (1.8, 3.1)	0.243
Peak mitral E' velocity (mm/sec)	-24.0 (-36.6, -18.9)	-22.4 (-25.7, -18.1)	0.447
Mitral E/E'	20.3 (14.5, 27.6)	34.9 (26.3, 41.3)	**0.003**

Statistical comparisons between groups were performed using the Mann-Whitney *U* test. Data are presented as the median (interquartile range).

Supplementary Table 3. Sequences of full-length and truncated *Sorbs2* cDNA constructs used in this study

Isoform	Sequence
**1. Murine *Sorbs2* (2193 bp).** Based on Addgene NCBI GenBank accession number KR610443, with the neuron specific exon deleted.<br><br>NT domain<br>SoHo domain<br>SH3-1, SH3-2, SH3-3	ATGAATACAGATAGCGGTGGGTGTGCTCGCAAACGTGCCGCCATGTCTGTTACGTTAACATCTGTGAAGAGAGTTCAAA<br>GTTCTCCAAACCTATTGGCTGCAGGGCGTGAGTCTCAGTCTCCAGATTCAGCAAAAGGTTTTAGAAGCGTTCGGCCAAA<br>CCTGCAAGACAAAAGATCACCGACCCAGAGCCAGATCACTATCAATGGCAACTCTGGTGGCGCCGTGAGTCCAGTGAGT<br>TACTATCAGAGGCCATTCTCCCCTTCTGCATACTCCCTCCCAGCCTCGCTGAACTCCAGCATTATCATGCAGCACGGCA<br>GGTCTCTTGATTCTGCAGAGACATATTCCCAGCATGCCCAGTCCCTGGATGGCACCATGGGAAGCTCCATCCCGCTCTA<br>TAGATCCTCCGAGGAAGAGAAGAGGGTCACGGTCATCAAAGCCCCGCATTACCCAGGGATCGGCCCTGTGGATGAGTCT<br>GGAATCCCCACAGCCATTAGGACGACTGTCGACAGGCCAAAGGACTGGTACAAGACAATGTTCAAGCAAATCCACATGG<br>TACACAAGCCGGGTCTGTACAACTCGCCCTACAGTGCTCAGTCCCACCCGGCTGCGAAGACTCAGACCTACAGACCTCT<br>TTCGAAAAGCCACTCAGACAATGGCACCGATGCGTTTAAGGAGGTGCCCTCCCCAGTGCCTCCCCCACACGTCCCACCA<br>CGACCAAGAGATCAGTCTTCAACACTAAAGCATGACTGGGACCCTCCAGACAGAAAGGTGGACACCAGGAAATTTCGAT<br>CGGAGCCAAGGAGTATTTTTGAATATGAGCCTGGGAAGTCGTCCATTCTGCAGCACGAAAGACCCCCCCTCTCCCACC<br>AACTCCGACACCTGTTCCCAGGGAACCCAGCCGGAAGCCTCTCTCAGTGTCACCCTCAACAGATGGCCTGCGTAGCCCT<br>TCCCCTCCGCCCAGGAGCTGTGTCCCCGCACCCCGTCCGAGTGCCCCAGATCTCTCTCCCACCCGGACGGGTCGCATAA<br>ATCCAGCTGACATAGATTTAGAAAATGAACCCTGGTATAAATTCTTTTCAGAACTGGAGTTTGGACACCCGCCTCCTAA<br>AAAGGCTCTGGATTATGTTCAAGATCATTCTTCCGGTGTGTCCAACGAGGTCTCCATCTATCAGTCCTCCATAGACAGA<br>AGCCTGGAAAGACCCAGCAGCTCTGCAAGCATGGCCGGTGACTTTAGGAAACGGAGGAAGAGCGAACCTGCAGTGGGCC<br>CACTCCGGGGCTTGGGGGATCAGAGTTCAAGCAGGACCAGCCCTGGCCGGGCGGACCTCCCAGGATCAAGTTCCACCTT<br>TACGAAGTCTTTCATTAGCTCTTCTCCTTCCTCTCCCTCGAGAGCACAAGATCACGAGTCCCCTAGAAGTTACTCATCT<br>ACTCTGACTGACTTGGGCAGAAGTGCATCACGGGAACGAAGGGGAACTCCAGAAAAAGAGAAATTGCCTGCAAAAGCTG<br>TCTATGATTTCAAAGCTCAGACATCTAAGGAGCTGTCATTTAAGAAAGGAGACACTGTCTACATCCTCAGGAAAATTGA<br>CCAAAACTGGTATGAGGGGGAACACCACGGCAGAGTGGGCATTTTCCCAATCTCATACGTAGAGAAACTAACACCCCCG<br>GAAAAAGCGCAGCCCGCAAGACCACCACCCCCAGTCCAGCCCGGAGAGATTGGAGAAGCCATAGCCAAGTACAACTTCA<br>ATGCAGACACAAATGTGGAACTCTCCCTGCGAAAGGGCGACAGGATTATTCTTCTTAAAAGAGTTGACCAAAACTGGTA<br>CGAAGGTAAAATCCCAGGAACCAACAGACAAGGCATCTTCCCCGTGTCCTATGTCGAGGTTGTCAAGAGGAACGCAAAA<br>GGTGCTGAGGACTACCCAGACCCTCCTCTGCCCCACAGCTACTCCAGTGACAGAATTTACACCCTAAGCTCCAATAAGC<br>CACAGCGTCCTGGGTTCTCTCATGAAAACATTCAAGGTGGGGGAGAACCGTTTCAGGCTCTGTATAACTATACTCCTAG<br>GAACGAaGATGAGCTGGAACTCAGAGAAAGTGATGTCGTTGATGTCATGGAAAAGTGTGACGACGGATGGTTTGTGGGA<br>ACTTCAAGAAGAACCAAATTCTTTGGTACTTTTCCCGGAAACTATGTCAAAAGGCTGTGA
***2. Sorbs2-ΔNT* (1770 bp)**	ATGGTCATCAAAGCCCCGCATTACCCAGGGATCGGCCCTGTGGATGAGTCTGGAATCCCCACAGCCATTAGGACGACTG<br>TCGACAGGCCAAAGGACTGGTACAAGACAATGTTCAAGCAAATCCACATGGTACACAAGCCGGGTCTGTACAACTCGCC

	CTACAGTGCTCAGTCCCACCCGGCTGCGAAGACTCAGACCTACAGACCTCTTTCGAAAAGCCACTCAGACAATGGCACC<br>GATGCGTTTAAGGAGGTGCCCTCCCCAGTGCCTCCCCCACACGTCCCACCACGACCAAGAGATCAGTCTTCAACACTAA<br>AGCATGACTGGGACCCTCCAGACAGAAAGGTGGACACCAGGAAATTTCGATCGGAGCCAAGGAGTATTTTTGAATATGA<br>GCCTGGGAAGTCGTCCATTCTGCAGCACGAAAGACCCCCCCCTCTCCCACCAACTCCGACACCTGTTCCCAGGGAACCC<br>AGCCGGAAGCCTCTCTCAGTGTCACCCTCAACAGATGGCCTGCGTAGCCCTTCCCCTCCGCCCAGGAGCTGTGTCCCCG<br>CACCCCGTCCGAGTGCCCCAGATCTCTCTCCCACCCGGACGGGTCGCATAAATCCAGCTGACATAGATTTAGAAAATGA<br>ACCCTGGTATAAATTCTTTTCAGAACTGGAGTTTGGACACCCGCCTCCTAAAAAGGCTCTGGATTATGTTCAAGATCAT<br>TCTTCCGGTGTGTCCAACGAGGTCTCCATCTATCAGTCCTCCATAGACAGAAGCCTGGAAAGACCCAGCAGCTCTGCAA<br>GCATGGCCGGTGACTTTAGGAAACGGAGGAAGAGCGAACCTGCAGTGGGCCCACTCCGGGGCTTGGGGGATCAGAGTTC<br>AAGCAGGACCAGCCCTGGCCGGGCGGACCTCCCAGGATCAAGTTCCACCTTTACGAAGTCTTTCATTAGCTCTTCTCCT<br>TCCTCTCCCTCGAGAGCACAAGATCACGAGTCCCCTAGAAGTTACTCATCTACTCTGACTGACTTGGGCAGAAGTGCAT<br>CACGGGAACGAAGGGGAACTCCAGAAAAAGAGAAATTGCCTGCAAAAGCTGTCTATGATTTCAAAGCTCAGACATCTAA<br>GGAGCTGTCATTTAAGAAAGGAGACACTGTCTACATCCTCAGGAAAATTGACCAAAACTGGTATGAGGGGGAACACCAC<br>GGCAGAGTGGGCATTTTCCCAATCTCATACGTAGAGAAACTAACACCCCCGGAAAAAGCGCAGCCCGCAAGACCACCAC<br>CCCCAGTCCAGCCCGGAGAGATTGGAGAAGCCATAGCCAAGTACAACTTCAATGCAGACACAAATGTGGAACTCTCCCT<br>GCGAAAGGGCGACAGGATTATTCTTCTTAAAAGAGTTGACCAAAACTGGTACGAAGGTAAAATCCCAGGAACCAACAGA<br>CAAGGCATCTTCCCCGTGTCCTATGTCGAGGTTGTCAAGAGGAACGCAAAAGGTGCTGAGGACTACCCAGACCCTCCTC<br>TGCCCCACAGCTACTCCAGTGACAGAATTTACACCCTAAGCTCCAATAAGCCACAGCGTCCTGGGTTCTCTCATGAAAA<br>CATTCAAGGTGGGGGAGAACCGTTTCAGGCTCTGTATAACTATACTCCTAGGAACGAaGATGAGCTGGAACTCAGAGAA<br>AGTGATGTCGTTGATGTCATGGAAAAGTGTGACGACGGATGGTTTGTGGGAACTTCAAGAAGAACCAAATTCTTTGGTA<br>CTTTTCCCGGAAACTATGTCAAAAGGCTGTGA
**3. S*orbs2-*Δ*Soho* (1998 bp)**	ATGAATACAGATAGCGGTGGGTGTGCTCGCAAACGTGCCGCCATGTCTGTTACGTTAACATCTGTGAAGAGAGTTCAAA<br>GTTCTCCAAACCTATTGGCTGCAGGGCGTGAGTCTCAGTCTCCAGATTCAGCAAAAGGTTTTAGAAGCGTTCGGCCAAA<br>CCTGCAAGACAAAAGATCACCGACCCAGAGCCAGATCACTATCAATGGCAACTCTGGTGGCGCCGTGAGTCCAGTGAGT<br>TACTATCAGAGGCCATTCTCCCCTTCTGCATACTCCCTCCCAGCCTCGCTGAACTCCAGCATTATCATGCAGCACGGCA<br>GGTCTCTTGATTCTGCAGAGACATATTCCCAGCATGCCCAGTCCCTGGATGGCACCATGGGAAGCTCCATCCCGCTCTA<br>TAGATCCTCCGAGGAAGAGAAGAGGGTCACGTACAGACCTCTTTCGAAAAGCCACTCAGACAATGGCACCGATGCGTTT<br>AAGGAGGTGCCCTCCCCAGTGCCTCCCCCACACGTCCCACCACGACCAAGAGATCAGTCTTCAACACTAAAGCATGACT<br>GGGACCCTCCAGACAGAAAGGTGGACACCAGGAAATTTCGATCGGAGCCAAGGAGTATTTTTGAATATGAGCCTGGGAA<br>GTCGTCCATTCTGCAGCACGAAAGACCCCCCCCTCTCCCACCAACTCCGACACCTGTTCCCAGGGAACCCAGCCGGAAG<br>CCTCTCTCAGTGTCACCCTCAACAGATGGCCTGCGTAGCCCTTCCCCTCCGCCCAGGAGCTGTGTCCCCGCACCCCGTC<br>CGAGTGCCCCAGATCTCTCTCCCACCCGGACGGGTCGCATAAATCCAGCTGACATAGATTTAGAAAATGAACCCTGGTA<br>TAAATTCTTTTCAGAACTGGAGTTTGGACACCCGCCTCCTAAAAAGGCTCTGGATTATGTTCAAGATCATTCTTCCGGT

	GTGTCCAACGAGGTCTCCATCTATCAGTCCTCCATAGACAGAAGCCTGGAAAGACCCAGCAGCTCTGCAAGCATGGCCG<br>GTGACTTTAGGAAACGGAGGAAGAGCGAACCTGCAGTGGGCCCACTCCGGGGCTTGGGGGATCAGAGTTCAAGCAGGAC<br>CAGCCCTGGCCGGGCGGACCTCCCAGGATCAAGTTCCACCTTTACGAAGTCTTTCATTAGCTCTTCTCCTTCCTCTCCC<br>TCGAGAGCACAAGATCACGAGTCCCCTAGAAGTTACTCATCTACTCTGACTGACTTGGGCAGAAGTGCATCACGGGAAC<br>GAAGGGGAACTCCAGAAAAAGAGAAATTGCCTGCAAAAGCTGTCTATGATTTCAAAGCTCAGACATCTAAGGAGCTGTC<br>ATTTAAGAAAGGAGACACTGTCTACATCCTCAGGAAAATTGACCAAAACTGGTATGAGGGGAACACCACGGCAGAGTG<br>GGCATTTTCCCAATCTCATACGTAGAGAAACTAACACCCCCGGAAAAAGCGCAGCCCGCAAGACCACCACCCCCAGTCC<br>AGCCCGGAGAGATTGGAGAAGCCATAGCCAAGTACAACTTCAATGCAGACACAAATGTGGAACTCTCCCTGCGAAAGGG<br>CGACAGGATTATTCTTCTTAAAAGAGTTGACCAAAACTGGTACGAAGGTAAAATCCCAGGAACCAACAGACAAGGCATC<br>TTCCCCGTGTCCTATGTCGAGGTTGTCAAGAGGAACGCAAAAGGTGCTGAGGACTACCCAGACCCTCCTCTGCCCCACA<br>GCTACTCCAGTGACAGAATTTACACCCTAAGCTCCAATAAGCCACAGCGTCCTGGGTTCTCTCATGAAAACATTCAAGG<br>TGGGGGAGAACCGTTTCAGGCTCTGTATAACTATACTCCTAGGAACGAaGATGAGCTGGAACTCAGAGAAAGTGATGTC<br>GTTGATGTCATGGAAAAGTGTGACGACGGATGGTTTGTGGGAACTTCAAGAAGAACCAAATTCTTTGGTACTTTTCCCG<br>GAAACTATGTCAAAAGGCTGTGA
**4. *Sorbs2-ΔSH3-1* (2013 bp)**	ATGAATACAGATAGCGGTGGGTGTGCTCGCAAACGTGCCGCCATGTCTGTTACGTTAACATCTGTGAAGAGAGTTCAAA<br>GTTCTCCAAACCTATTGGCTGCAGGGCGTGAGTCTCAGTCTCCAGATTCAGCAAAAGGTTTTAGAAGCGTTCGGCCAAA<br>CCTGCAAGACAAAAGATCACCGACCCAGAGCCAGATCACTATCAATGGCAACTCTGGTGGCGCCGTGAGTCCAGTGAGT<br>TACTATCAGAGGCCATTCTCCCCTTCTGCATACTCCCTCCCAGCCTCGCTGAACTCCAGCATTATCATGCAGCACGGCA<br>GGTCTCTTGATTCTGCAGAGACATATTCCCAGCATGCCCAGTCCCTGGATGGCACCATGGGAAGCTCCATCCCGCTCTA<br>TAGATCCTCCGAGGAAGAGAAGAGGGTCACGGTCATCAAAGCCCCGCATTACCCAGGGATCGGCCCTGTGGATGAGTCT<br>GGAATCCCCACAGCCATTAGGACGACTGTCGACAGGCCAAAGGACTGGTACAAGACAATGTTCAAGCAAATCCACATGG<br>TACACAAGCCGGGTCTGTACAACTCGCCCTACAGTGCTCAGTCCCACCCGGCTGCGAAGACTCAGACCTACAGACCTCT<br>TTCGAAAAGCCACTCAGACAATGGCACCGATGCGTTTAAGGAGGTGCCCTCCCCAGTGCCTCCCCCACACGTCCCACCA<br>CGACCAAGAGATCAGTCTTCAACACTAAAGCATGACTGGGACCCTCCAGACAGAAAGGTGGACACCAGGAAATTTCGAT<br>CGGAGCCAAGGAGTATTTTTGAATATGAGCCTGGGAAGTCGTCCATTCTGCAGCACGAAAGACCCCCCCCTCTCCCACC<br>AACTCCGACACCTGTTCCCAGGGAACCCAGCCGGAAGCCTCTCTCAGTGTCACCCTCAACAGATGGCCTGCGTAGCCCT<br>TCCCCTCCGCCCAGGAGCTGTGTCCCCGCACCCCGTCCGAGTGCCCCAGATCTCTCTCCCACCCGGACGGGTCGCATAA<br>ATCCAGCTGACATAGATTTAGAAAATGAACCCTGGTATAAATTCTTTTCAGAACTGGAGTTTGGACACCCGCCTCCTAA<br>AAAGGCTCTGGATTATGTTCAAGATCATTCTTCCGGTGTGTCCAACGAGGTCTCCATCTATCAGTCCTCCATAGACAGA<br>AGCCTGGAAAGACCCAGCAGCTCTGCAAGCATGGCCGGTGACTTTAGGAAACGGAGGAAGAGCGAACCTGCAGTGGGCC<br>CACTCCGGGGCTTGGGGGATCAGAGTTCAAGCAGGACCAGCCCTGGCCGGGCGGACCTCCCAGGATCAAGTTCCACCTT<br>TACGAAGTCTTTCATTAGCTCTTCTCCTTCCTCTCCCTCGAGAGCACAAGATCACGAGTCCCCTAGAAGTTACTCATCT<br>ACTCTGACTGACTTGGGCAGAAGTGCATCACGGGAACGAAGGGGAACTCCAGAACCGGAAAAAGCGCAGCCCGCAAGAC

	CACCACCCCCAGTCCAGCCCGGAGAGATTGGAGAAGCCATAGCCAAGTACAACTTCAATGCAGACACAAATGTGGAACT<br>CTCCCTGCGAAAGGGCGACAGGATTATTCTTCTTAAAAGAGTTGACCAAAACTGGTACGAAGGTAAAATCCCAGGAACC<br>AACAGACAAGGCATCTTCCCCGTGTCCTATGTCGAGGTTGTCAAGAGGAACGCAAAAGGTGCTGAGGACTACCCAGACC<br>CTCCTCTGCCCCACAGCTACTCCAGTGACAGAATTTACACCCTAAGCTCCAATAAGCCACAGCGTCCTGGGTTCTCTCA<br>TGAAAACATTCAAGGTGGGGGAGAACCGTTTCAGGCTCTGTATAACTATACTCCTAGGAACGAaGATGAGCTGGAACTC<br>AGAGAAAGTGATGTCGTTGATGTCATGGAAAAGTGTGACGACGGATGGTTTGTGGGAACTTCAAGAAGAACCAAATTCT<br>TTGGTACTTTTCCCGGAAACTATGTCAAAAGGCTGTGA
**5. *Sorbs2-ΔSH3-2* (2007 bp)**	ATGAATACAGATAGCGGTGGGTGTGCTCGCAAACGTGCCGCCATGTCTGTTACGTTAACATCTGTGAAGAGAGTTCAAA<br>GTTCTCCAAACCTATTGGCTGCAGGGCGTGAGTCTCAGTCTCCAGATTCAGCAAAAGGTTTTAGAAGCGTTCGGCCAAA<br>CCTGCAAGACAAAAGATCACCGACCCAGAGCCAGATCACTATCAATGGCAACTCTGGTGGCGCCGTGAGTCCAGTGAGT<br>TACTATCAGAGGCCATTCTCCCCTTCTGCATACTCCCTCCCAGCCTCGCTGAACTCCAGCATTATCATGCAGCACGGCA<br>GGTCTCTTGATTCTGCAGAGACATATTCCCAGCATGCCCAGTCCCTGGATGGCACCATGGGAAGCTCCATCCCGCTCTA<br>TAGATCCTCCGAGGAAGAGAAGAGGGTCACGGTCATCAAAGCCCCGCATTACCCAGGGATCGGCCCTGTGGATGAGTCT<br>GGAATCCCCACAGCCATTAGGACGACTGTCGACAGGCCAAAGGACTGGTACAAGACAATGTTCAAGCAAATCCACATGG<br>TACACAAGCCGGGTCTGTACAACTCGCCCTACAGTGCTCAGTCCCACCCGGCTGCGAAGACTCAGACCTACAGACCTCT<br>TTCGAAAAGCCACTCAGACAATGGCACCGATGCGTTTAAGGAGGTGCCCTCCCCAGTGCCTCCCCCACACGTCCCACCA<br>CGACCAAGAGATCAGTCTTCAACACTAAAGCATGACTGGGACCCTCCAGACAGAAAGGTGGACACCAGGAAATTTCGAT<br>CGGAGCCAAGGAGTATTTTTGAATATGAGCCTGGGAAGTCGTCCATTCTGCAGCACGAAAGACCCCCCCCTCTCCCACC<br>AACTCCGACACCTGTTCCCAGGGAACCCAGCCGGAAGCCTCTCTCAGTGTCACCCTCAACAGATGGCCTGCGTAGCCCT<br>TCCCCTCCGCCCAGGAGCTGTGTCCCCGCACCCCGTCCGAGTGCCCCAGATCTCTCTCCCACCCGGACGGGTCGCATAA<br>ATCCAGCTGACATAGATTTAGAAAATGAACCCTGGTATAAATTCTTTTCAGAACTGGAGTTTGGACACCCGCCTCCTAA<br>AAAGGCTCTGGATTATGTTCAAGATCATTCTTCCGGTGTGTCCAACGAGGTCTCCATCTATCAGTCCTCCATAGACAGA<br>AGCCTGGAAAGACCCAGCAGCTCTGCAAGCATGGCCGGTGACTTTAGGAAACGGAGGAAGAGCGAACCTGCAGTGGGCC<br>CACTCCGGGGCTTGGGGGATCAGAGTTCAAGCAGGACCAGCCCTGGCCGGGCGGACCTCCCAGGATCAAGTTCCACCTT<br>TACGAAGTCTTTCATTAGCTCTTCTCCTTCCTCTCCCTCGAGAGCACAAGATCACGAGTCCCCTAGAAGTTACTCATCT<br>ACTCTGACTGACTTGGGCAGAAGTGCATCACGGGAACGAAGGGGAACTCCAGAAAAAGAGAAATTGCCTGCAAAAGCTG<br>TCTATGATTTCAAAGCTCAGACATCTAAGGAGCTGTCATTTAAGAAAGGAGACACTGTCTACATCCTCAGGAAAATTGA<br>CCAAAACTGGTATGAGGGGGAACACCACGGCAGAGTGGGCATTTTCCCAATCTCATACGTAGAGAAACTAACACCCCCG<br>GAAAAAGCGCAGCCCGCAAGACCACCACCCCCAGTCCAGCCCAACGCAAAAGGTGCTGAGGACTACCCAGACCCTCCTC<br>TGCCCCACAGCTACTCCAGTGACAGAATTTACACCCTAAGCTCCAATAAGCCACAGCGTCCTGGGTTCTCTCATGAAAA<br>CATTCAAGGTGGGGGAGAACCGTTTCAGGCTCTGTATAACTATACTCCTAGGAACGAaGATGAGCTGGAACTCAGAGAA<br>AGTGATGTCGTTGATGTCATGGAAAAGTGTGACGACGGATGGTTTGTGGGAACTTCAAGAAGAACCAAATTCTTTGGTA<br>CTTTTCCCGGAAACTATGTCAAAAGGCTGTGA

***6. Sorbs2-ΔSH3-3* (2013 bp)**	ATGAATACAGATAGCGGTGGGTGTGCTCGCAAACGTGCCGCCATGTCTGTTACGTTAACATCTGTGAAGAGAGTTCAAA<br>GTTCTCCAAACCTATTGGCTGCAGGGCGTGAGTCTCAGTCTCCAGATTCAGCAAAAGGTTTTAGAAGCGTTCGGCCAAA<br>CCTGCAAGACAAAAGATCACCGACCCAGAGCCAGATCACTATCAATGGCAACTCTGGTGGCGCCGTGAGTCCAGTGAGT<br>TACTATCAGAGGCCATTCTCCCCTTCTGCATACTCCCTCCCAGCCTCGCTGAACTCCAGCATTATCATGCAGCACGGCA<br>GGTCTCTTGATTCTGCAGAGACATATTCCCAGCATGCCCAGTCCCTGGATGGCACCATGGGAAGCTCCATCCCGCTCTA<br>TAGATCCTCCGAGGAAGAGAAGAGGGTCACGGTCATCAAAGCCCCGCATTACCCAGGGATCGGCCCTGTGGATGAGTCT<br>GGAATCCCCACAGCCATTAGGACGACTGTCGACAGGCCAAAGGACTGGTACAAGACAATGTTCAAGCAAATCCACATGG<br>TACACAAGCCGGGTCTGTACAACTCGCCCTACAGTGCTCAGTCCCACCCGGCTGCGAAGACTCAGACCTACAGACCTCT<br>TTCGAAAAGCCACTCAGACAATGGCACCGATGCGTTTAAGGAGGTGCCCTCCCCAGTGCCTCCCCCACACGTCCCACCA<br>CGACCAAGAGATCAGTCTTCAACACTAAAGCATGACTGGGACCCTCCAGACAGAAAGGTGGACACCAGGAAATTTCGAT<br>CGGAGCCAAGGAGTATTTTTGAATATGAGCCTGGGAAGTCGTCCATTCTGCAGCACGAAAGACCCCCCCCTCTCCCACC<br>AACTCCGACACCTGTTCCCAGGGAACCCAGCCGGAAGCCTCTCTCAGTGTCACCCTCAACAGATGGCCTGCGTAGCCCT<br>TCCCCTCCGCCCAGGAGCTGTGTCCCCGCACCCCGTCCGAGTGCCCCAGATCTCTCTCCCACCCGGACGGGTCGCATAA<br>ATCCAGCTGACATAGATTTAGAAAATGAACCCTGGTATAAATTCTTTTCAGAACTGGAGTTTGGACACCCGCCTCCTAA<br>AAAGGCTCTGGATTATGTTCAAGATCATTCTTCCGGTGTGTCCAACGAGGTCTCCATCTATCAGTCCTCCATAGACAGA<br>AGCCTGGAAAGACCCAGCAGCTCTGCAAGCATGGCCGGTGACTTTAGGAAACGGAGGAAGAGCGAACCTGCAGTGGGCC<br>CACTCCGGGGCTTGGGGGATCAGAGTTCAAGCAGGACCAGCCCTGGCCGGGCGGACCTCCCAGGATCAAGTTCCACCTT<br>TACGAAGTCTTTCATTAGCTCTTCTCCTTCCTCTCCCTCGAGAGCACAAGATCACGAGTCCCCTAGAAGTTACTCATCT<br>ACTCTGACTGACTTGGGCAGAAGTGCATCACGGGAACGAAGGGGAACTCCAGAAAAAGAGAAATTGCCTGCAAAAGCTG<br>TCTATGATTTCAAAGCTCAGACATCTAAGGAGCTGTCATTTAAGAAAGGAGACACTGTCTACATCCTCAGGAAAATTGA<br>CCAAAACTGGTATGAGGGGGAACACCACGGCAGAGTGGGCATTTTCCCAATCTCATACGTAGAGAAACTAACACCCCCG<br>GAAAAAGCGCAGCCCGCAAGACCACCACCCCCAGTCCAGCCCGGAGAGATTGGAGAAGCCATAGCCAAGTACAACTTCA<br>ATGCAGACACAAATGTGGAACTCTCCCTGCGAAAGGGCGACAGGATTATTCTTCTTAAAAGAGTTGACCAAAACTGGTA<br>CGAAGGTAAAATCCCAGGAACCAACAGACAAGGCATCTTCCCCGTGTCCTATGTCGAGGTTGTCAAGAGGAACGCAAAA<br>GGTGCTGAGGACTACCCAGACCCTCCTCTGCCCCACAGCTACTCCAGTGACAGAATTTACACCCTAAGCTCCAATAAGC<br>CACAGCGTCCTGGGTTCTCTCATGAAAACATTCAATGA

Supplementary Table 4. Body weight, heart weight, and lung weight of 6-month-old WT and KO mice treated with AAV as indicated

	WT untreated (n=9)	KO Untreated (n=9)	KO+ SORBS2 (n=9)	KO+ △NT (n=8)	KO+ △SoHo (n=7)	KO+ △SH3-1 (n=7)	KO+ △SH3-2 (n=7)	KO+ △SH3-3 (n=7)
BW (g)	29.1±3.5	25.9±2.7*	24.7±3.5	25.4±2.2*	25.8±1.3*	24.4±2.7*	25.8±4.4	27.2±2.1
TL (mm)	17.6±0.5	17.2±0.7	17.2±0.6	17.2±0.3	17.3±0.5	16.7±2.2	18.0±0.9‡	17.0±0.8
HW (mg)	149.6±27.8	154.2±47.8	124.8±20.3	129.4±25.4	126.0±5.0*	152.7±56.8	151.0±51.0	149.7±29.7
RA (mg)	3.5±0.8	4.6±2.4	3.4±0.7	4.7±2.4	3.5±0.5	5.6±1.7*‡	4.6±1.9	4.1±0.8
LA (mg)	3.4±1.1	14.9±4.3*	3.4±0.9†	5.7±4.8	7.8±2.6*‡	21.7±27.7*	18.6±21.8	17.3±18.5
LW (mg)	148.0±13.6	215.1±74.2*	145.0±22.5†	158.8±25.7	160.7±17.8	184.0±40.6*‡	192.0±21.7*‡	187.5±44.6*‡
HW/BW (mg/g)	5.1±0.6	5.9±1.3	5.0±0.3	5.1±1.0	4.9±0.3	6.3±2.7	5.8±1.7	5.6±1.6
LW/BW (mg/g)	5.1±0.6	8.3±2.4*	5.9±0.5†	6.3±0.8*	6.3±0.8*	7.6±2.1*	7.6±1.1*‡	7.0±2.3*
HW/TL (mg/mm)	8.5±1.5	9.0±2.9*	7.3±1.0	7.5±1.4	7.3±0.4	9.2±3.1	8.4±2.7	8.9±2.2
LW/TL (mg/mm)	8.4±0.7	12.5±4.5	8.4±1.0†	9.3±1.6	9.3±0.9*	11.2±2.6*‡	10.7±0.9*‡	11.1±3.2*‡
RA/HW (%)	2.3±0.4	3.1±1.1	2.8±0.6	3.5±1.3*	2.8±0.4	3.7±0.6*‡	3.1±1.1	2.8±0.4
LA/HW (%)	2.2±0.4	9.9±2.12*	2.8±0.6†	4.3±2.8	6.2±2.2*‡	11.7±9.5*‡	10.6±8.5*‡	10.3±7.8*‡

One-way ANOVA with Tukey HSD multiple comparison test. Data are presented as mean ± SD.

* p<0.05 vs. WT-untreated
† p<0.05 vs. KO-untreated
‡ p<0.05 vs. KO+SORBS2

Supplementary Table 5. Echocardiographic parameters of 6-month-old WT and KO mice treated with AAV as indicated

Parameter	WT untreated (n=8)	KO untreated (n=10)	KO+ SORBS2 (n=9)	KO+ ΔNT (n=7)	KO+ ΔSoHo (n=7)	KO+ ΔSH3-1 (n=7)	KO+ ΔSH3-2 (n=7)	KO+ ΔSH3-3 (n=7)
HR (bpm)	434.3±34.1	398.0±40.0	389.1±12.7	391.3±10.8	397.7±44.3	413.6±32.6	393.7±43.2	403.9±47.0
LVEF (%)	54.4±4.4	51.4±7.0	49.8±5.5	49.8±2.7	52.0±7.8	50.8±5.5	50.1±9.4	51.0±9.5
LVFS (%)	27.9±2.9	26.0±4.2	25.0±3.4	24.3±3.0	26.5±4.8	25.7±3.4	25.5±5.7	26.2±5.8
LVID, s (mm)	2.9±0.2	2.9±0.4	3.1±0.3	3.4±0.3	3.0±0.3	3.1±0.3	3.3±0.4	3.3±0.5
LVID, d (mm)	4.0±0.3	3.9±0.5	4.2±0.3	4.4±0.4	4.1±0.2	4.1±0.4	4.4±0.4	4.5±0.5
IVS, d (mm)	0.8±0.1	0.7±0.1	0.7±0.1	0.7±0.1	0.7±0.1	0.8±0.3	0.8±0.2	0.8±0.2
LVPW, d (mm)	0.7±0.1	0.8±0.3	0.7±0.1	0.6±0.1	0.7±0.2	0.6±0.1	0.6±0.1	0.7±0.2
LV mass (mg)	88.7±14.4	89.7±30.5	79.1±8.3	88.7±15.6	81.3±13.6	82.1±17.5	95.0±25.5	103.5±16.1
LA (mm)	1.8±0.2	2.3±0.5	1.9±0.1	2.3±0.5	2.0±0.2	2.3±0.4	2.4±0.5*	2.3±0.4
Peak mitral E velocity (mm/sec)	549.2±67.3	953.9±181.6*	661.8±102.2†	741.6±281.1	796.0±134.3	863.3±165.2*	1050.4±273.6*†	875.5±155.2*
Peak mitral A velocity (mm/sec)	281.1±51.9	347.4±76.3	308.9±92.3	312.3±207.2	456.1±54.9	336.3±70.3	489.8±281.1	426.7±146.3
Mitral E/A	2.0±0.3	2.8±0.7	2.2±0.4	2.7±0.9	1.8±0.5	2.7±1.0	2.7±1.3	2.5±1.5
Peak mitral E' velocity (mm/sec)	-26.1±5.1	-20.3±3.0	-24.2±6.1	-19.1±2.7*	-16.4±1.6*‡	-17.8±5.4*	-21.0±3.6	-16.8±1.8*‡
Mitral E/E'	21.7±4.5	47.9±12.0*	28.9±8.7	38.8±13.2	48.7±8.5*‡	51.1±14.0*‡	50.3±11.3*‡	52.7±11.7*‡

One-way ANOVA with Tukey HSD multiple comparison test. Data are presented as mean ± SD.

* p<0.05 vs. WT-untreated

† p<0.05 vs. KO-untreated

‡ p<0.05 vs. KO+SORBS2

Supplementary Table 6. Top proteins identified through proximity proteomics

Gene Symbol	Rank	Average intensity		(BioID-SORBS2+ 1)/(Ctrl+1) ratio
		BioID-SORBS2 group	Ctrl group	
Sorbs2	1	2.40E+08	0	2.40E+08
Crk	2	1.60E+08	0	1.60E+08
Prob1	3	3.70E+07	0	3.70E+07
Gpd2	4	2.40E+07	0	2.40E+07
Enah	5	1.30E+07	0	1.30E+07
Ptger4	6	1.30E+07	0	1.30E+07
Crebrf	7	1.10E+07	0	1.10E+07
Sorbs2	8	8.60E+06	0	8.60E+06
Sorbs2	9	8.20E+06	0	8.20E+06
Tnfaip3	10	5.80E+06	0	5.80E+06
Sorbs1	11	5.50E+06	0	5.50E+06
Cdh2	12	5.40E+06	0	5.40E+06
Irf2bpl	13	4.40E+06	0	4.40E+06
Ablim1	14	4.30E+06	0	4.30E+06
Cip2a	15	4.10E+06	0	4.10E+06
Lpp	16	4.00E+06	0	4.00E+06
Lamb1	17	3.80E+06	0	3.80E+06
Dcaf12	18	3.80E+06	0	3.80E+06
Nebl	19	3.50E+06	0	3.50E+06
Myb	20	3.50E+06	0	3.50E+06
Ctnna1	21	3.40E+06	0	3.40E+06
Cycs	22	3.30E+06	0	3.30E+06

Zfhx3	23	3.20E+06	0	3.20E+06
**Gene Symbol**	**Rank**	**Average intensity**		**(BioID-SORBS2+ 1)/(Ctrl+1) ratio**
		**BioID-SORBS2 group**	**Ctrl group**	
Hmcn2	24	2.90E+06	0	2.90E+06
Pdlim4	25	2.60E+06	0	2.60E+06
Cpq	26	2.60E+06	0	2.60E+06
Mcoln3	27	2.50E+06	0	2.50E+06
Tln1	28	2.40E+06	0	2.40E+06
Fzd3	29	2.20E+06	0	2.20E+06
Syne1	30	2.00E+06	0	2.00E+06
Cct5	31	2.00E+06	0	2.00E+06
Ctnnb1	32	1.90E+06	0	1.90E+06
Rab11fip1	33	1.90E+06	0	1.90E+06
Lmo7	34	1.90E+06	0	1.90E+06
Sfn	35	1.80E+06	0	1.80E+06
Ctnna3	36	1.80E+06	0	1.80E+06
Arhgef28	37	1.60E+06	0	1.60E+06
Cav1	38	1.60E+06	0	1.60E+06
Synpo2	39	1.50E+06	0	1.50E+06
Sorbs2	40	1.50E+06	0	1.50E+06
Hspa12b	41	1.50E+06	0	1.50E+06
Tln2	42	1.50E+06	0	1.50E+06
Hbb-b2	43	1.50E+06	0	1.50E+06
Snta1	44	1.50E+06	0	1.50E+06
Rps26	45	1.30E+06	0	1.30E+06
Nppa	46	1.30E+06	0	1.30E+06
Vapb	47	1.30E+06	0	1.30E+06

Frg1	48	1.30E+06	0	1.30E+06
**Gene Symbol**	**Rank**	**Average intensity**		**(BioID-SORBS2+ 1)/(Ctrl+1) ratio**
		**BioID-SORBS2 group**	**Ctrl group**	
Spta1	49	1.20E+06	0	1.20E+06
Rexo1	50	1.20E+06	0	1.20E+06
Mast3	51	1.20E+06	0	1.20E+06
Dock8	52	1.20E+06	0	1.20E+06
Hspb6	53	1.10E+06	0	1.10E+06
Ywhaq	54	1.10E+06	0	1.10E+06
Mlip	55	1.00E+06	0	1.00E+06
Ablim1	56	1.00E+06	0	1.00E+06
Afdn	57	1.00E+06	0	1.00E+06
Prdx2	58	9.50E+05	0	9.50E+05
Dnaja3	59	9.30E+05	0	9.30E+05
Src	60	9.20E+05	0	9.20E+05
Arf5	61	9.00E+05	0	9.00E+05
Palld	62	8.60E+05	0	8.60E+05
Coro6	63	8.60E+05	0	8.60E+05
Mapre2	64	8.50E+05	0	8.50E+05
Sart1	65	8.40E+05	0	8.40E+05
Csrp1	66	8.20E+05	0	8.20E+05
Lama5	67	8.20E+05	0	8.20E+05
Cand2	68	8.10E+05	0	8.10E+05
Kank3	69	8.10E+05	0	8.10E+05
Ryr3	70	8.00E+05	0	8.00E+05
Rps8	71	7.80E+05	0	7.80E+05
Ccdc141	72	7.70E+05	0	7.70E+05

Gene Symbol	Rank	Average intensity		(BioID-SORBS2+ 1)/(Ctrl+1) ratio
		BioID-SORBS2 group	Ctrl group	
Ppib	73	7.70E+05	0	7.70E+05
Sorbs1	74	7.60E+05	0	7.60E+05
Nebl	75	7.60E+05	0	7.60E+05
Eif4h	76	7.60E+05	0	7.60E+05
Flna	77	7.30E+05	0	7.30E+05
Scaf11	78	7.20E+05	0	7.20E+05
Rai14	79	7.10E+05	0	7.10E+05
Fermt2	80	6.90E+05	0	6.90E+05
Clasp1	81	6.90E+05	0	6.90E+05
Immt	82	6.80E+05	0	6.80E+05
Dcn	83	6.70E+05	0	6.70E+05
Gm8765	84	6.70E+05	0	6.70E+05
Cmya5	85	6.60E+05	0	6.60E+05
Rplp2	86	6.60E+05	0	6.60E+05
Rpl18a	87	6.40E+05	0	6.40E+05
Nid2	88	6.40E+05	0	6.40E+05
Psmc2	89	6.30E+05	0	6.30E+05
Skt	90	6.30E+05	0	6.30E+05
Ppm1k	91	6.20E+05	0	6.20E+05
Actn1	92	6.20E+05	0	6.20E+05
Nipsnap2	93	6.20E+05	0	6.20E+05
Hnrnpa2b1	94	6.10E+05	0	6.10E+05
Pcbp2	95	6.10E+05	0	6.10E+05
Arih2	96	6.00E+05	0	6.00E+05
Sptb	97	5.90E+05	0	5.90E+05

Gene Symbol	Rank	Average intensity		(BioID-SORBS2+ 1)/(Ctrl+1) ratio
		BioID-SORBS2 group	Ctrl group	
Lrpprc	98	5.90E+05	0	5.90E+05
Papln	99	5.70E+05	0	5.70E+05
Pkp2	100	5.60E+05	0	5.60E+05
Tjp1	101	5.50E+05	0	5.50E+05
Mtfr1l	102	5.40E+05	0	5.40E+05
Aldoa	103	5.30E+05	0	5.30E+05
Sorbs1	104	5.10E+05	0	5.10E+05
Erc1	105	5.00E+05	0	5.00E+05
Ogdhl	106	4.70E+05	0	4.70E+05
Clu	107	4.50E+05	0	4.50E+05
Cacna1d	108	4.50E+05	0	4.50E+05
Sorbs1	109	4.30E+05	0	4.30E+05
Pdlim5	110	4.10E+05	0	4.10E+05
Mrps15	111	4.00E+05	0	4.00E+05
Dnaja2	112	4.00E+05	0	4.00E+05
Hnrnpk	113	3.80E+05	0	3.80E+05
Dync1h1	114	3.80E+05	0	3.80E+05
Upf1	115	3.70E+05	0	3.70E+05
Cct8	116	3.40E+05	0	3.40E+05
Picalm	117	3.40E+05	0	3.40E+05
Pdlim1	118	3.30E+05	0	3.30E+05
Ttn	119	3.30E+05	0	3.30E+05
Mlip	120	3.10E+05	0	3.10E+05
Sorbs1	121	3.10E+05	0	3.10E+05
Rdh14	122	2.80E+05	0	2.80E+05

Gene Symbol	Rank	Average intensity		(BioID-SORBS2+ 1)/(Ctrl+1) ratio
		BioID-SORBS2 group	Ctrl group	
Txlnb	123	2.70E+05	0	2.70E+05
Sec24c	124	2.60E+05	0	2.60E+05
Abce1	125	2.50E+05	0	2.50E+05
Homer1	126	2.30E+05	0	2.30E+05
Hspa1b	127	2.30E+05	0	2.30E+05
Vdac3	128	2.30E+05	0	2.30E+05
Mrrf	129	1.80E+05	0	1.80E+05
Psmd2	130	1.70E+05	0	1.70E+05
Erf	131	1.70E+05	0	1.70E+05
Myo18b	132	1.10E+05	0	1.10E+05
Rhoa	133	1.00E+05	0	1.00E+05
Dvl2	134	4.20E+04	0	4.20E+04
Speg	1	3.40E+07	5.40E+05	62.62
Txn	2	3.20E+07	1.20E+06	27.03
Ahnak	3	3.70E+07	1.40E+06	26.29
Tln2	4	1.10E+07	5.80E+05	18.51
Sorbs1	5	9.10E+06	6.30E+05	14.44
Col6a2	6	5.20E+07	4.20E+06	12.48
Aifm1	7	3.70E+07	3.00E+06	12.40
Ryr2	8	6.20E+06	5.90E+05	10.56
Vcl	9	9.00E+06	9.90E+05	9.06
Ttn	10	3.20E+07	3.70E+06	8.71
Ldb3	11	1.10E+08	1.40E+07	8.20
Crat	12	1.60E+06	2.60E+05	6.27
Plec	13	4.10E+06	7.30E+05	5.55

Plin4	14	1.80E+07	3.40E+06	5.18
**Gene Symbol**	**Rank**	**Average intensity**		**(BioID-SORBS2+ 1)/(Ctrl+1) ratio**
		**BioID-SORBS2 group**	**Ctrl group**	
Pcx	15	5.70E+06	1.20E+06	4.78
Hspa5	16	2.60E+06	5.50E+05	4.77
Flnc	17	2.80E+06	6.30E+05	4.53
Nebl	18	3.10E+06	7.10E+05	4.37
Dsp	19	1.90E+06	4.60E+05	4.07
Dtna	20	5.90E+05	1.50E+05	4.06
Mdh2	21	1.10E+06	2.90E+05	3.91
Hbb-b1	22	5.50E+06	1.50E+06	3.71
Lama4	23	7.30E+05	2.10E+05	3.46
Xirp1	24	6.90E+05	2.00E+05	3.43
Cox6b1	25	4.60E+06	1.40E+06	3.29
Limch1	26	4.60E+06	1.40E+06	3.28
Rpl17	27	9.70E+05	3.10E+05	3.13
Slc25a20	28	2.90E+06	9.40E+05	3.11

Supplementary Table 7. Oligonucleotides used in this study

	genotyping primers		
**Genotype**	**primer 1**	**primer 2**	**common primer**
Sorbs2 $^{e8/e8}$	GCTACCATTACCAGTTGGTCTGGTGTC	AGAAGTTGTAGAAGTACGAGGGTTTCC	AACAACAAAAACCCCAAACCAAACCG

	qPCR primer	
**Gene**	**primer 1**	**primer 2**
**mouse *Gapdh***	GGATAAACCTGCCAACTATGATGAC	GTAGCCCAGGATGCCCTTTAGT
**mouse *Sorbs2***	CTATCAGAGGCCATTCTCCC	GAACATTGTCTTGTACCAGTCC
**mouse *Nppa***	TACAGTGCGGTGTCCAACACAG	TGCTTCCTCAGTCTGCTCACTC
**mouse *Nppb***	GCTGCTGGAGCTGATAAGAGAA	GTTCTTTTGTAGGGCCTTGGTC
**mouse *Col1a1***	GAGAGAGCATGACCGATGGATT	GCTACGCTGTTCTTGCAGTGAT
**mouse *Col3a***	CAGCAGTCCAACGTAGATGAATTG	CATGGTTCTGGCTTCCAGACA

	primer for titin RT-PCR	
**primer**	**target region**	**sequence**
**E50F**	Exon 50, Forward	GAGATCCAAGGAGCCCCCAA
**E51R**	Exon 51, Reverse	TCTGCCAGATGATCTCAATCAC
**E219R**	Exon 219, Reverse	GCACCACCTCTTCCTTTGGC

Supplementary Table 8. Antibodies used in this study

Antigen	Antibody Manufacturer	Cat No	IF	WB	WES	IP	PLA
SORBS2	Proteintech	24643-1-AP	1:100	1:1000			
SORBS2	Sigma	SAB4200183	1:200	1:1000			
HA tag	Cell Signaling Techology	3724	1:100	1:500	1:800	1 µg /100 µg protein	1:100
IgG mouse	Santa Cruz Biotechnology	sc-2025	-	-	-	1 µg per 100 µg	
IgG Rabbit	Rockland Immunochemicals	43708	-	-	-	1 µg per 100 µg	
GAPDH	Cell Signaling Techology	2118S	-	1:2000	1:800		
α-tubulin	Thermo fisher	501726981	1:100	1:1000			
detyrosinated tubulin	Abcam	ab48389	1:100	1:1000			
detyrosinated tubulin	Abcam	ab254154	1:200	1:1000			
TTL	thermo fisher	13618-1-AP		1:500			
RYR2	Sigma	R128	1:100	1:500			1:100
CACNA1C	Alomone labs	ACC-003	1:100	1:200			
JPH2	Thermofisher	40-5300	1:100	1:500		-	-
SERCA	Santa Cruz	sc-376235	-	1:1000	1:500		
PLN	Cell Signaling Technology	14562	-	1:1000	1:500		
Phospho-PLN (S16)	Abcam	ab92697	-	1:1000	1:500		
Desmin	Sigma	D1033	1:200	1:1000			
connexin 43	Abcam	ab11370	1:200	1:500			
N-cadherin	Origene	AB0071-200	1:25	1:500			
N-cadherin	Life Technologies	333900	1-3ug/ml	1:500			
vinculin	Cell Signaling Techology	13901		1:1000	1:500		
ACTN2	Sigma-Aldrich	A7811	1:100	1:500	1:500		
GFP goat	Rockland Antibodies and Assays	600-101-215	1:500				

Antigen	Antibody Manufacturer	Cat No	IF	WB	WES	IP	PLA
Phalloidin	Life Technologies	A12379	1:1000				
Streptavidin-HRP	Invitrogen	SA10001		1:3000			
Donkey-anti-Mouse HRP	Rockland Immunochemicals	610-703-124	-	1:3000			
Donkey-anti-Rabbit HRP	Rockland Immunochemicals	610-703-127	-	1:3000			
Donkey-anti-Mouse 488	Invitrogen	A32766	1:400	-			
Donkey-anti-Rabbit 488	Invitrogen	A32790	1:400	-			
Donkey-anti-Mouse 555	Invitrogen	A31570	1:400	-			
Donkey-anti-Rabbit 555	Invitrogen	A31572	1:400	-			
Donkey-anti-Mouse 647	Invitrogen	A31571	1:400	-			
Donkey-anti-Rabbit 647	Invitrogen	A31573	1:400	-			

IF, immunofluorescent staining. WB, western blotting. WES, capillary western. IP, immunoprecipitation. PLA, proximity ligation assay.

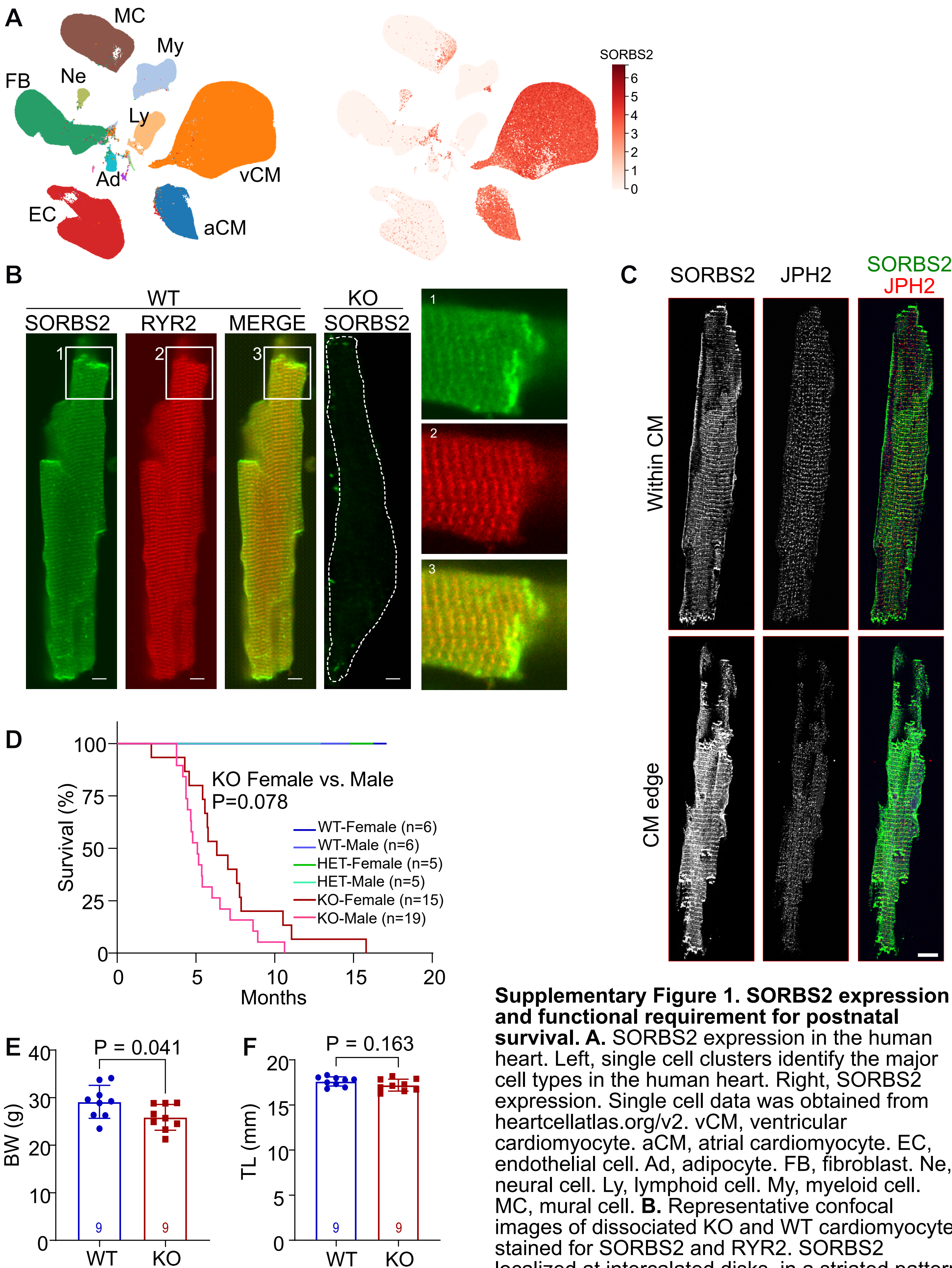

Supplementary Figure 1. SORBS2 expression and functional requirement for postnatal survival. A. SORBS2 expression in the human heart. Left, single cell clusters identify the major cell types in the human heart. Right, SORBS2 expression. Single cell data was obtained from heartcellatlas.org/v2. vCM, ventricular cardiomyocyte. aCM, atrial cardiomyocyte. EC, endothelial cell. Ad, adipocyte. FB, fibroblast. Ne, neural cell. Ly, lymphoid cell. My, myeloid cell. MC, mural cell. **B.** Representative confocal images of dissociated KO and WT cardiomyocytes stained for SORBS2 and RYR2. SORBS2 localized at intercalated disks, in a striated pattern

Supplementary Figure 1. continued.

striated pattern coincident with RYR2 (cardiac dyads), and at the cell margin. Bar = 10 µm. **C.** Confocal z-stack imaging followed by post-acquisition deconvolution of a WT cardiomyocyte immunostained for SORBS2 and JPH2 (T-tubules). Top row, image plane through middle of the cardiomyocyte. Bottom row, image plane tangenial to the surface of the cardiomyocyte. SORBS2 was present at the edge of cardiomyocytes and near T-tubules that penetrate deep within the cell. Bar = 10 µm. The z-stack is provided as Supplementary Data 1. **D.** Survival of male vs female *Sorbs2* KO, Het, and WT mice. **E-F**. Comparison of *Sorbs2* KO and WT body weight (BW) and tibia length (TL). *t*-test. Graphs show mean ± SD.

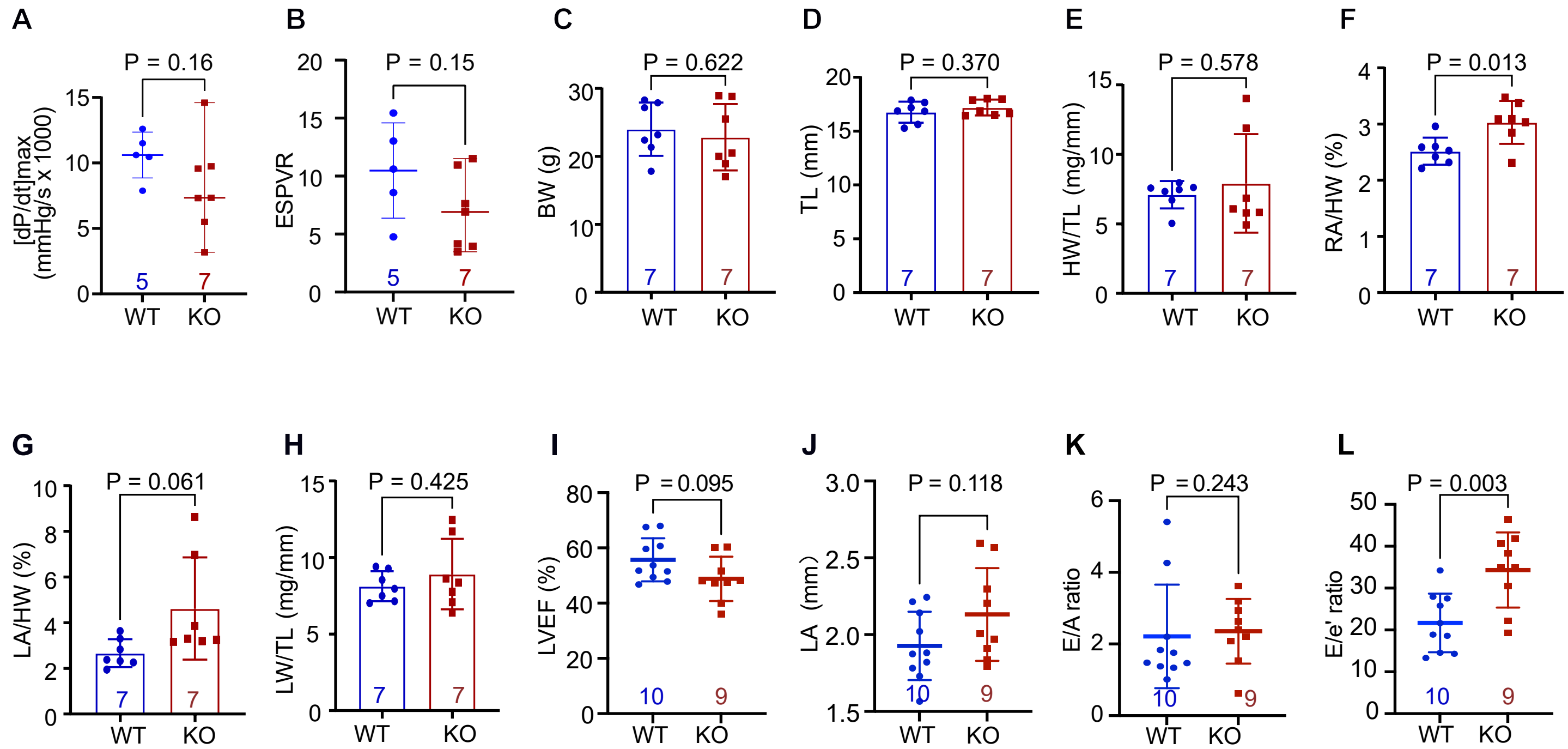

Supplementary Figure 2. Functional analysis of *Sorbs2* KO and WT hearts. A-B. Hemodynamic assessment of systolic function at 6 months of age. **A.** Maximum rate of change of pressure over time. Mann-Whitney *U test.* Graphs show the median and interquartile range (IQR). **B.** End systolic pressure volume relationship (ESPVR). Mann-Whitney *U test.* Graphs show the median and IQR.**C-H.** KO vs WT heart at 3 months of age. Body weight (BW), tibia length (TL), heart weight to tibia length (HW/TL), right atrium to heart weight (RA/HW), left atrium to heart weight (LA/HW), and lung weight to tibia length (LW/TL). *t*-test. Graphs show mean ± SD. **I-L.** Echocardiographic changes at 3 months of age in WT and KO mice. **I.** Left ventricular ejection fraction (LVEF). Mann-Whitney *U test.* Graphs show the median and IQR. **J.** left atrial diameter (LA). Mann-Whitney *U test.* Graphs show the median and IQR. **K-L.** Echocardiographic measures of diastolic function at 3 months of age: early to late mitral inflow velocity (E/A) ratio, and early mitral inflow to early mitral annular velocity (E/e') ratio. Mann-Whitney *U test.* Graphs show the median and IQR.

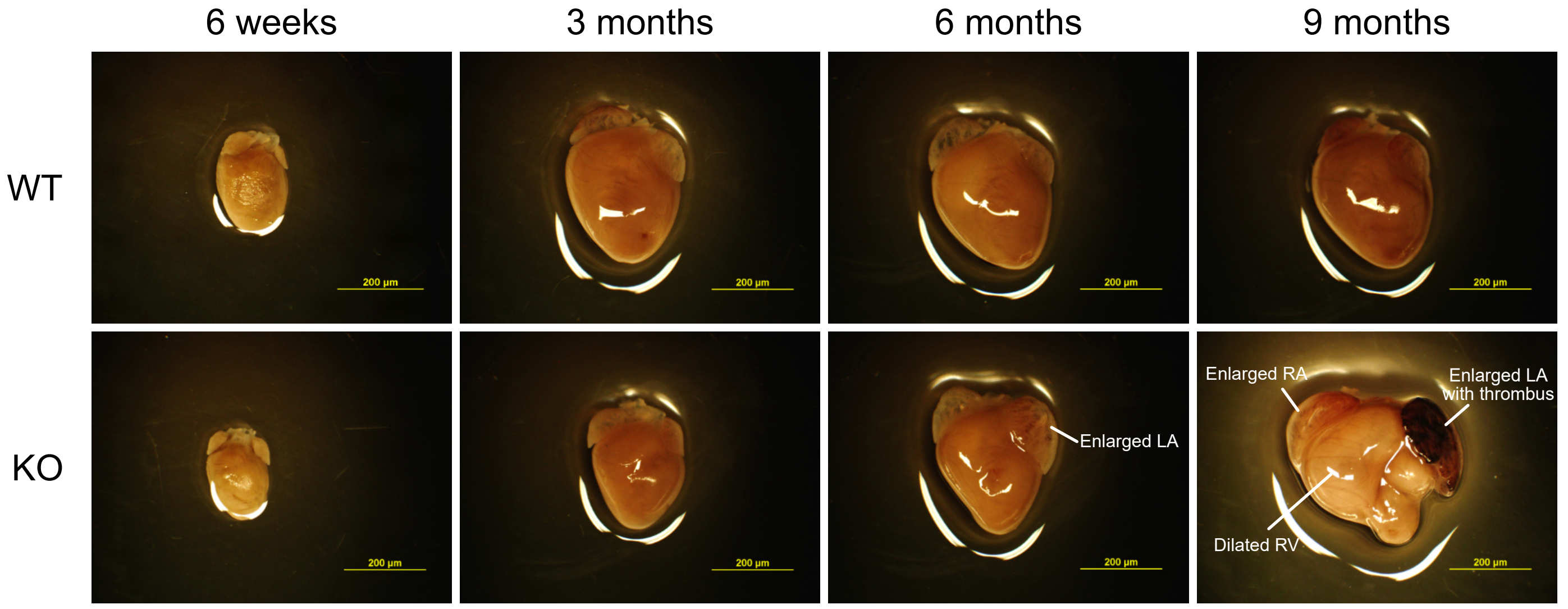

Supplementary Figure 3. Progressive cardiac remodeling in *Sorbs2* KO mice. Representative gross heart images from WT and *Sorbs2* KO mice at 6 weeks, 3 months, 6 months, and 9 months of age. WT hearts maintain normal size and morphology across all time points. In contrast, *Sorbs2* KO hearts display age-dependent cardiac remodeling, characterized by progressive atrial enlargement, with left atrial (LA) dilation evident by 6 months, followed by marked biatrial enlargement and right ventricular dilation by 9 months. Left atrial thrombus formation is frequently observed at the late stage, consistent with advanced atrial remodeling and progression to heart failure. Scale bar = 200 μm.

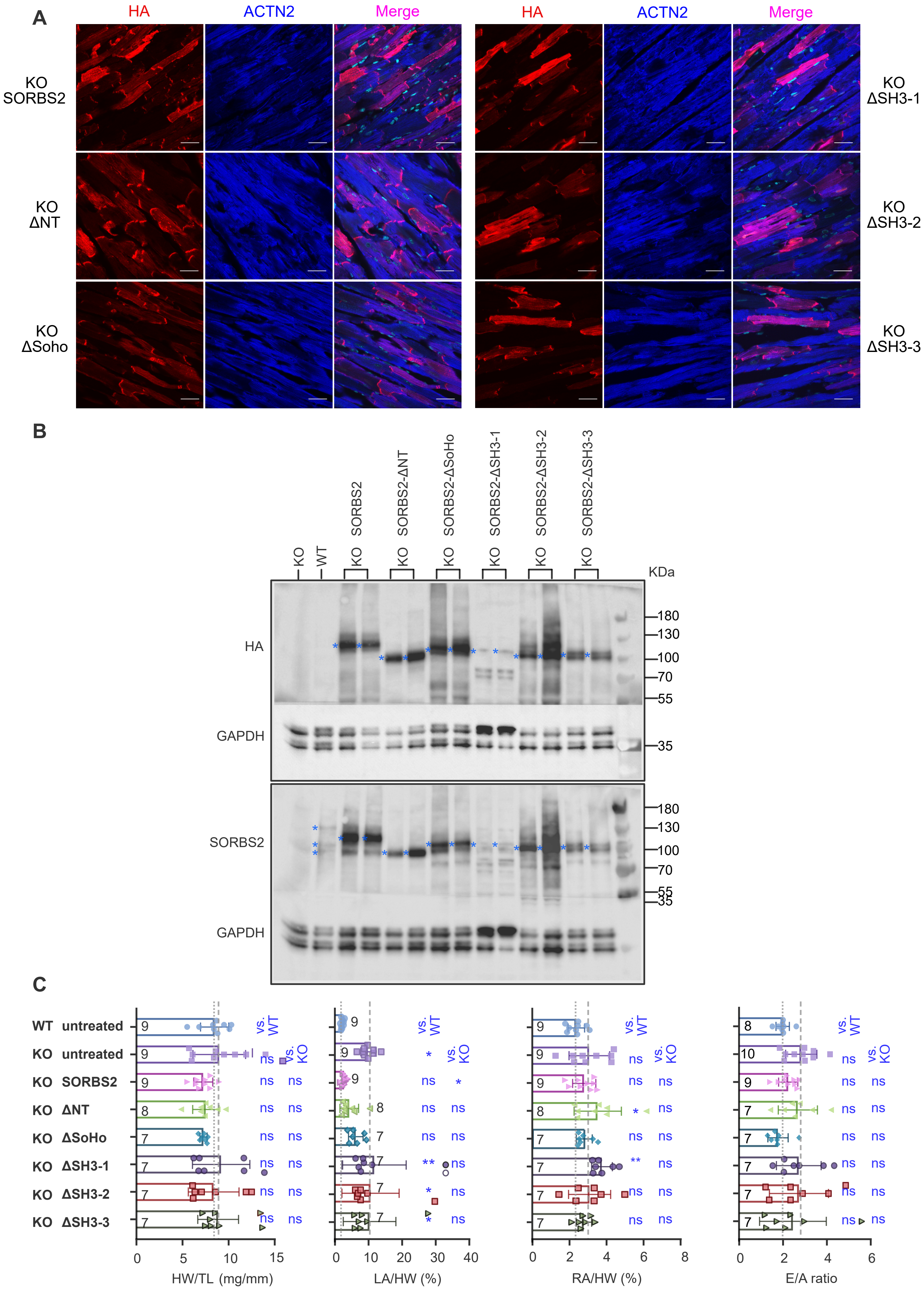
A
HA
ACTN2
Merge
KO SORBS2
KO ΔNT
KO ΔSoho
KO ΔSH3-1
KO ΔSH3-2
KO ΔSH3-3
B
KO
WT
KO SORBS2
KO SORBS2-ΔNT
KO SORBS2-ΔSoHo
KO SORBS2-ΔSH3-1
KO SORBS2-ΔSH3-2
KO SORBS2-ΔSH3-3
KDa
HA
GAPDH
SORBS2
180
130
100
70
55
35
C
WT untreated
KO untreated
KO SORBS2
KO ΔNT
KO ΔSoHo
KO ΔSH3-1
KO ΔSH3-2
KO ΔSH3-3
vs. WT
vs. KO
HW/TL (mg/mm)
LA/HW (%)
RA/HW (%)
E/A ratio

Supplementary Figure 4. Effect of AAV9-SORBS2 on *Sorbs2* KO heart function. A. Subcellular localiation of SORBS2 and truncated SORBS2 in cardiomyocytes. Representative immunofluorescence images of heart sections from KO mice transduced with AAV expressing HA-tagged SORBS2 or HA- tagged truncated SORBS2 mutants (ΔNT, ΔSoHo, ΔSH3-1, ΔSH3-2, and ΔSH3-3). Sections were stained for HA (red) to detect exogenous SORBS2 constructs and ACTN2 (blue) to label cardiomyocyte Z-discs. Bar = 20 μm. **B.** Western blot showing expression of HA-tagged SORBS2 in the heart. WT or KO neonates were treated with the indicated AAV. Heart lysates were collected from adult mice and analyzed by western blotting for HA, SORBS2, or GAPDH. Blue asterisks indicated SORBS2 bands. **C.** Cardiac parameters measured at 6 months of age. HT/TL, heart weight to tibia length ratio; LA/HW, left atria to heart weight ratio; RA/HW, right atria to heart weight ratio; E/A, early to late diastolic filling ratio. One way ANOVA with Dunnett's post hoc test vs WT or KO reference group. ns, $P \geq 0.05$. *, $P<0.05$. **, $P<0.01$.Graphs show mean ± SD.

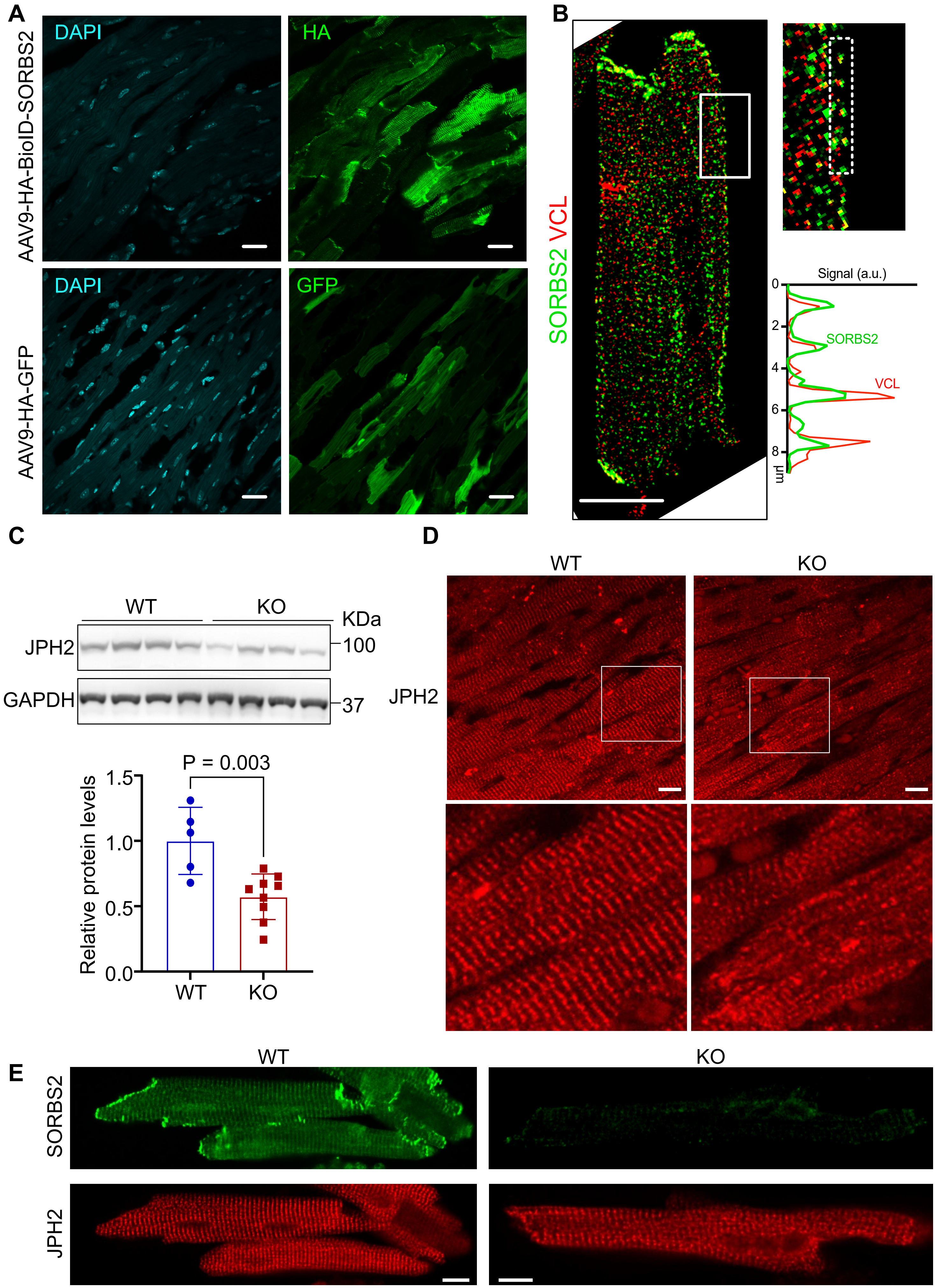
A
AAV9-HA-BioID-SORBS2
AAV9-HA-GFP
DAPI
HA
DAPI
GFP
B
SORBS2 VCL
Signal (a.u.)
SORBS2
VCL
µm
0
2
4
6
8
C
WT
KO
KDa
JPH2
GAPDH
100
37
P = 0.003
Relative protein levels
1.5
1.0
0.5
0.0
WT
KO
D
WT
KO
JPH2
E
WT
KO
SORBS2
JPH2

Supplementary Figure 5. Localization of BirA*-SORBS2, SORBS2, and JPH2. A. Wild type mice transduced with AAV-BioID-HA-SORBS2 or AAV-HA-GFP. Heart sections were immunostained and imaged by confocal microscopy. Bar = 20 μm. **B.** Confocal imaging of WT cardiomyocyte immunostained for SORBS2 and vinculin (VCL). Boxed area is enlarged, and signal within the dotted box is plotted below. Bar, 10 μm. **C.** Immunoblots of WT and *Sorbs2* KO heart lysates at 6 months of age. *t*-test. Graphs show mean ± SD. **D.** Heart sections from WT or KO stained for dyadic protein JPH2. Boxed regions are enlarged below. Bar = 10 μm. **E.** WT or KO dissociated cardiomyocytes stained for SORBS2 and JPH2. Bar = 10 μm.

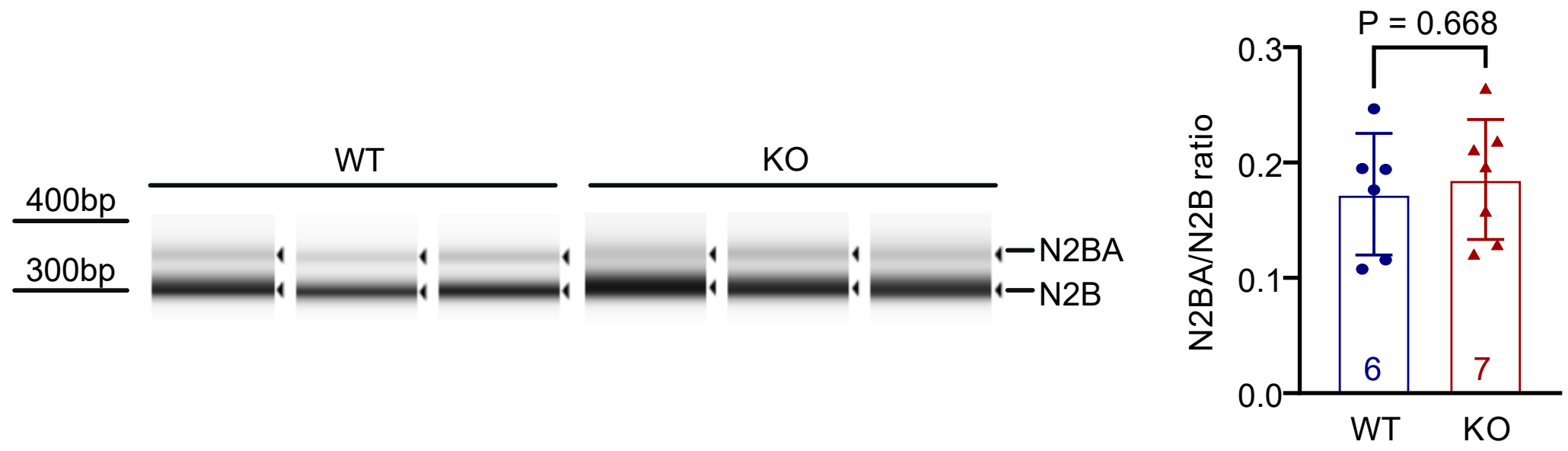

Supplementary Figure 6. RT-PCR analysis of titin isoform expression in *Sorbs2* KO and WT. RT-PCR products for the comliant (N2BA) and stiff (N2B) assays were separated by agarose gel electrophoresis (left). Quantification of band intensity did not show a significant change in N2BA to N2B ratio. Graph shows mean ± SD. *t*-test.

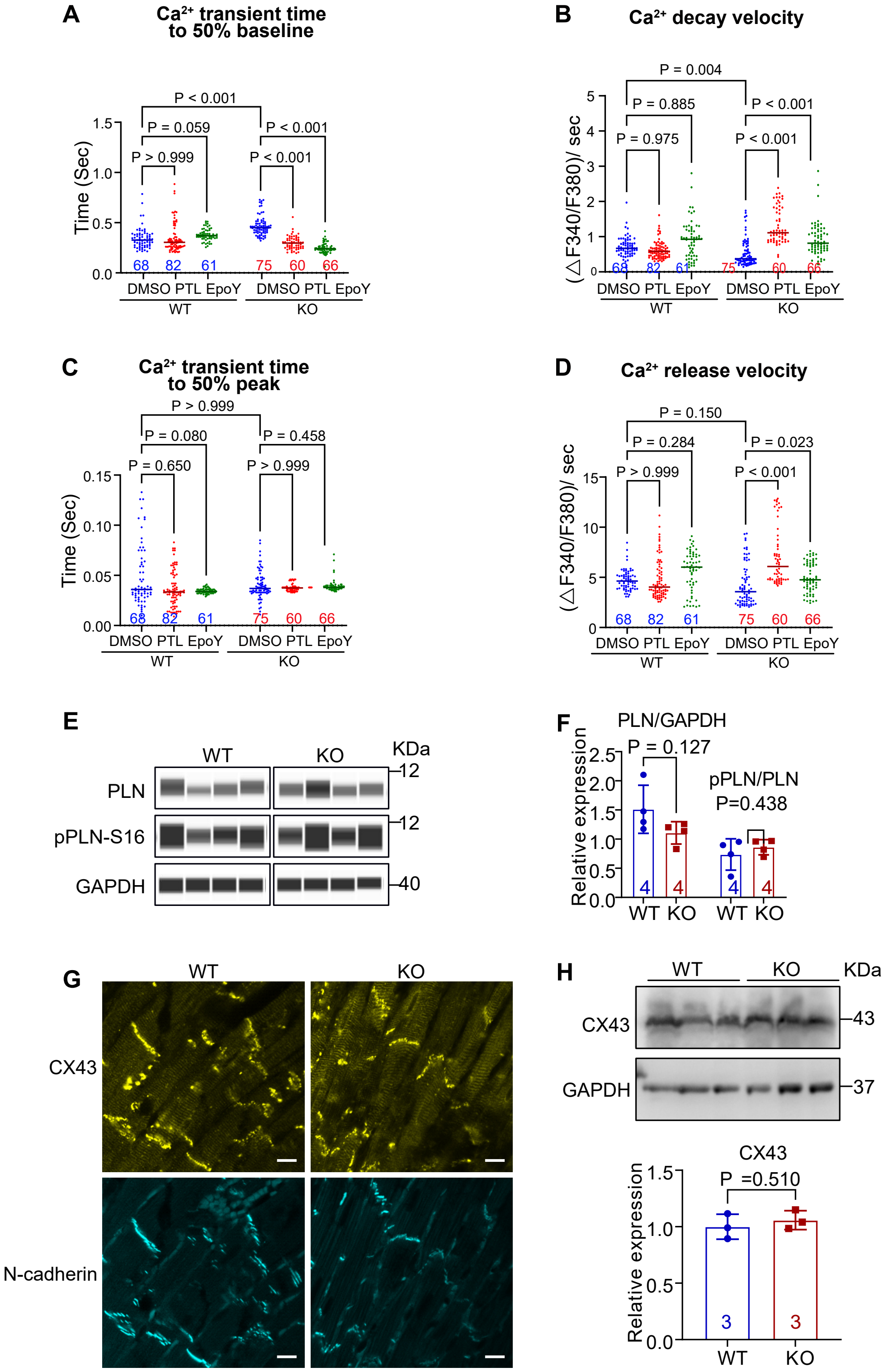

Supplementary Figure 7. Characterization of *Sorbs2* KO cardiomyocytes. A-D. Dissociated cardiomyocytes from 6-month-old WT and KO mice were cultured for 2 hours with PTL or EpoY, and loaded with Fura-2. Whole cell Ca^{2+} dynamics were analyzed using the ratio of the signal at 340 and 380 nm. Kruskal-Wallis with Dunn's multiple comparison test. Line indicates median value. **E-F.** Heart lysates were immunoblotted to measure the relative levels of PLN and PLN phosphorylated on serine 16 (pPLN-S16), normalized to GAPDH. *t*-test. Graph shows mean ± SD. **G.** KO and WT heart sections were stained for CX43 and N-cadherin. We did not observe significant changes in signal intensity or distribution between genotypes. Bar = 10 μm. **H.** Cx43 western blotting. No significant change in CX43 protein levels were observed. *t*-test. Graphs show mean ± SD.

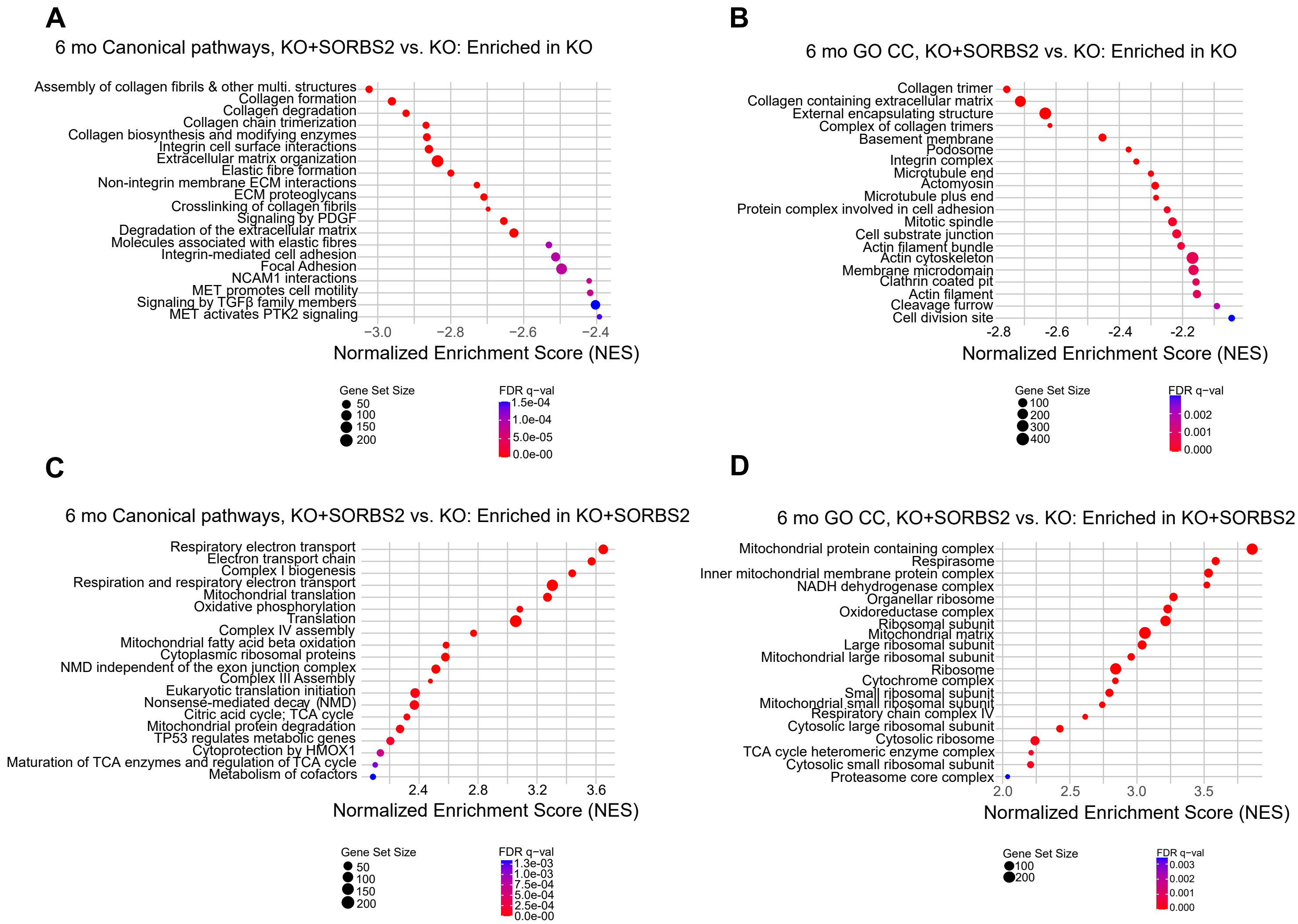

Supplementary Figure 8. Comparisons of gene expression between KO and KO+SORBS2 hearts. 6-month-old hearts from KO or KO treated with AAV-SORBS2 were analyzed by RNA-seq. Differentially expressed genes were analyzed for gene ontology (GO) term canonical pathway or cellular component (CC) enrichment.
