# Supplementary material for: SORBS2 regulates diastolic function through cytoskeletal networks and calcium handling
Source: Commun Biol. 2026 Jun 17;9:1291. doi: 10.1038/s42003-026-10503-6 (PMC13639047; doi:10.1038/s42003-026-10503-6)
Supplement: Supplementary file 2 [file 42003_2026_10503_MOESM2_ESM.pdf]

Description of Additional Supplementary Files

File name: Supplementary Data 1

Description: Zip file of a 16-bit TIF z-stack of an isolated adult cardiomyocyte stained for SORBS2, JPH2, and nuclei. Open in ImageJ.

File name: Supplementary Data 2

Description: SORBS2 BioID proximity proteomics

File name: Supplementary Data 3

Description: Union of DEGs between Sorbs2 KO and control in 6w and 6m hearts.

File name: Supplementary Data 4

Description: Uncropped gels.

File name: Supplementary Data 5

Description: Numerical source data.
