# Supplementary material for: SORBS2 regulates diastolic function through cytoskeletal networks and calcium handling
Source: Commun Biol. 2026 Jun 17;9:1291. doi: 10.1038/s42003-026-10503-6 (PMC13639047; doi:10.1038/s42003-026-10503-6)

Fig. 1C

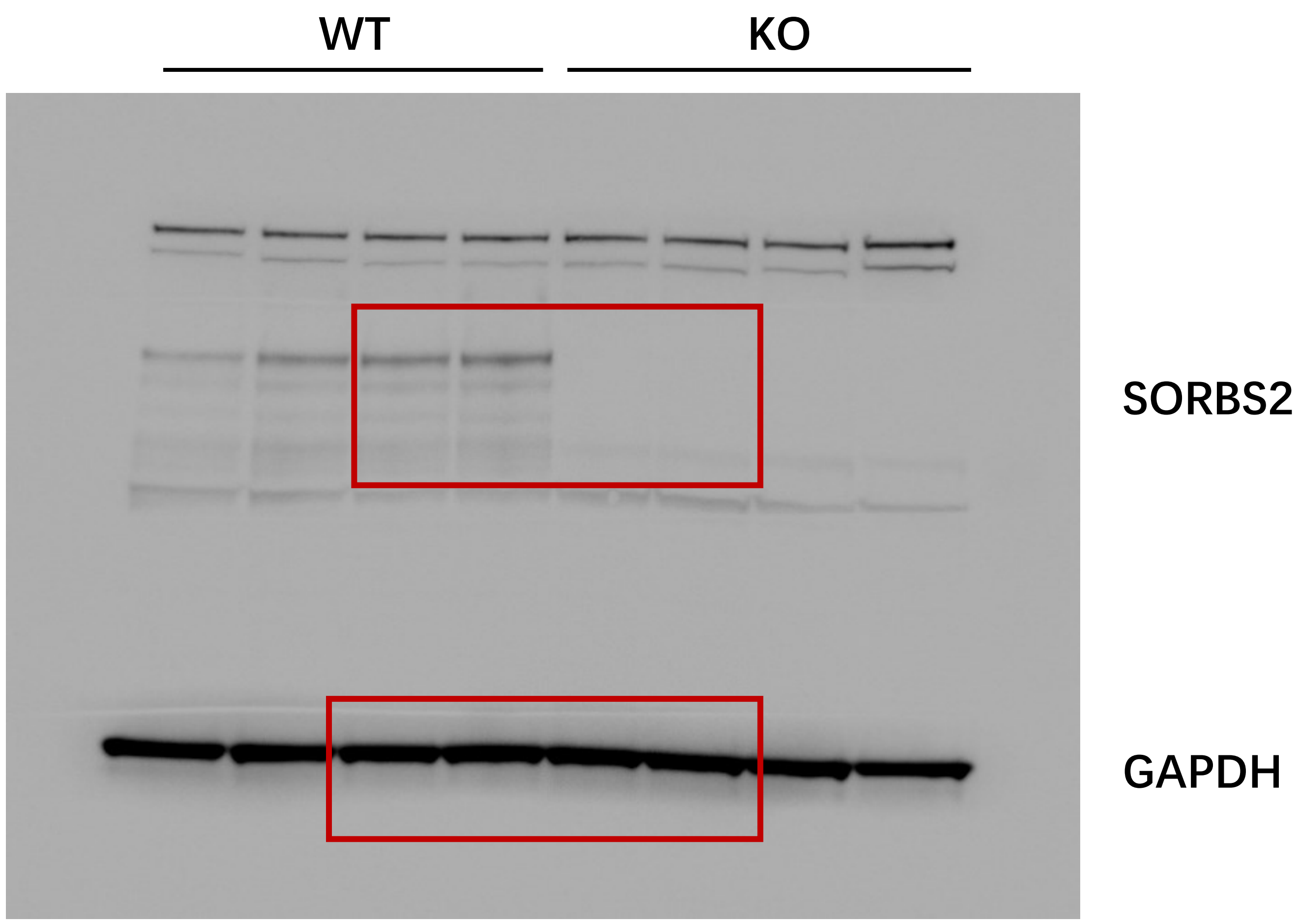

Fig. 4B

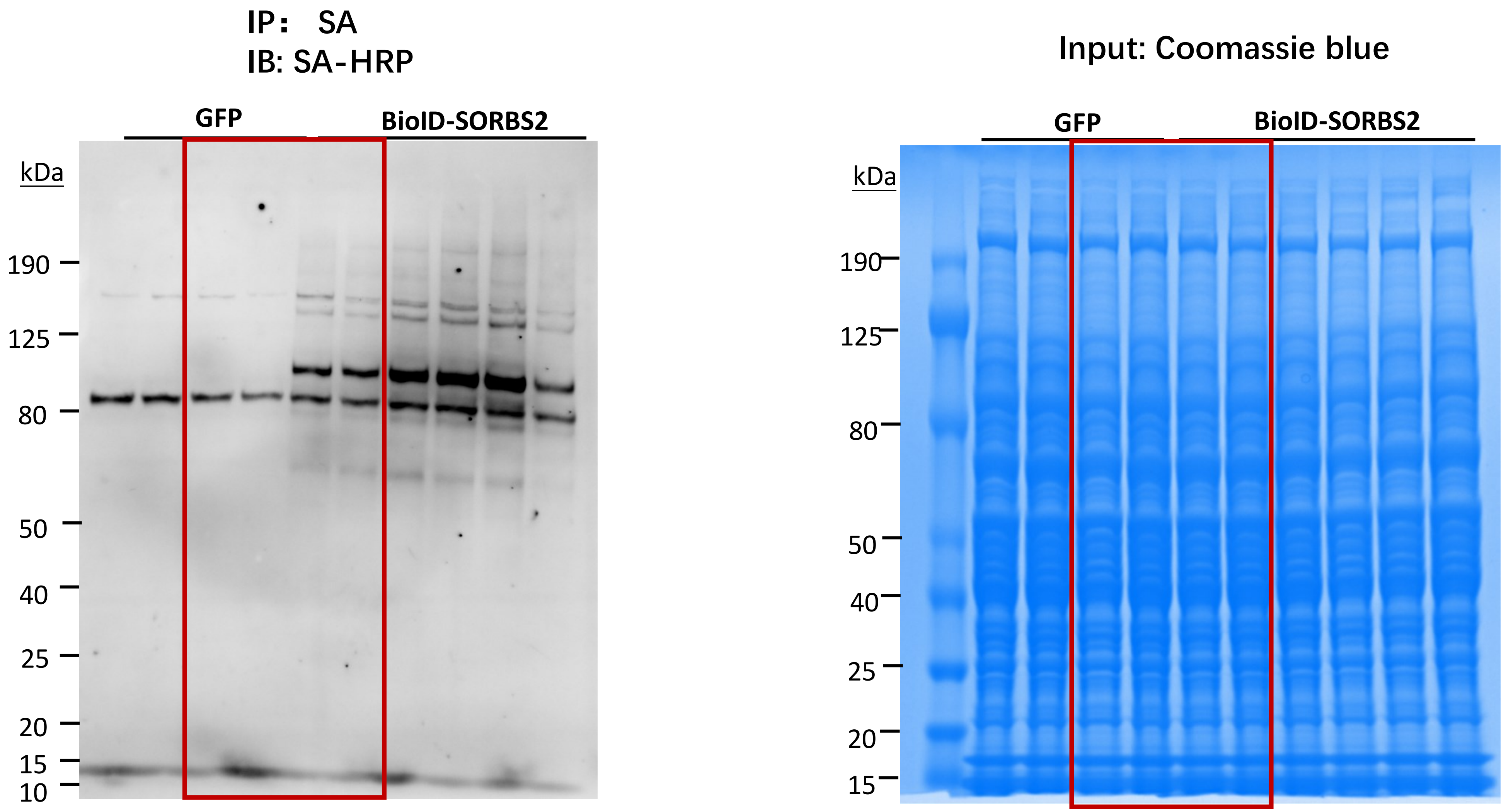
IP: SA
IB: SA-HRP
GFP
BioID-SORBS2
kDa
190
125
80
50
40
25
20
15
10
Input: Coomassie blue
GFP
BioID-SORBS2
kDa
190
125
80
50
40
25
20
15

Fig. 4D-1

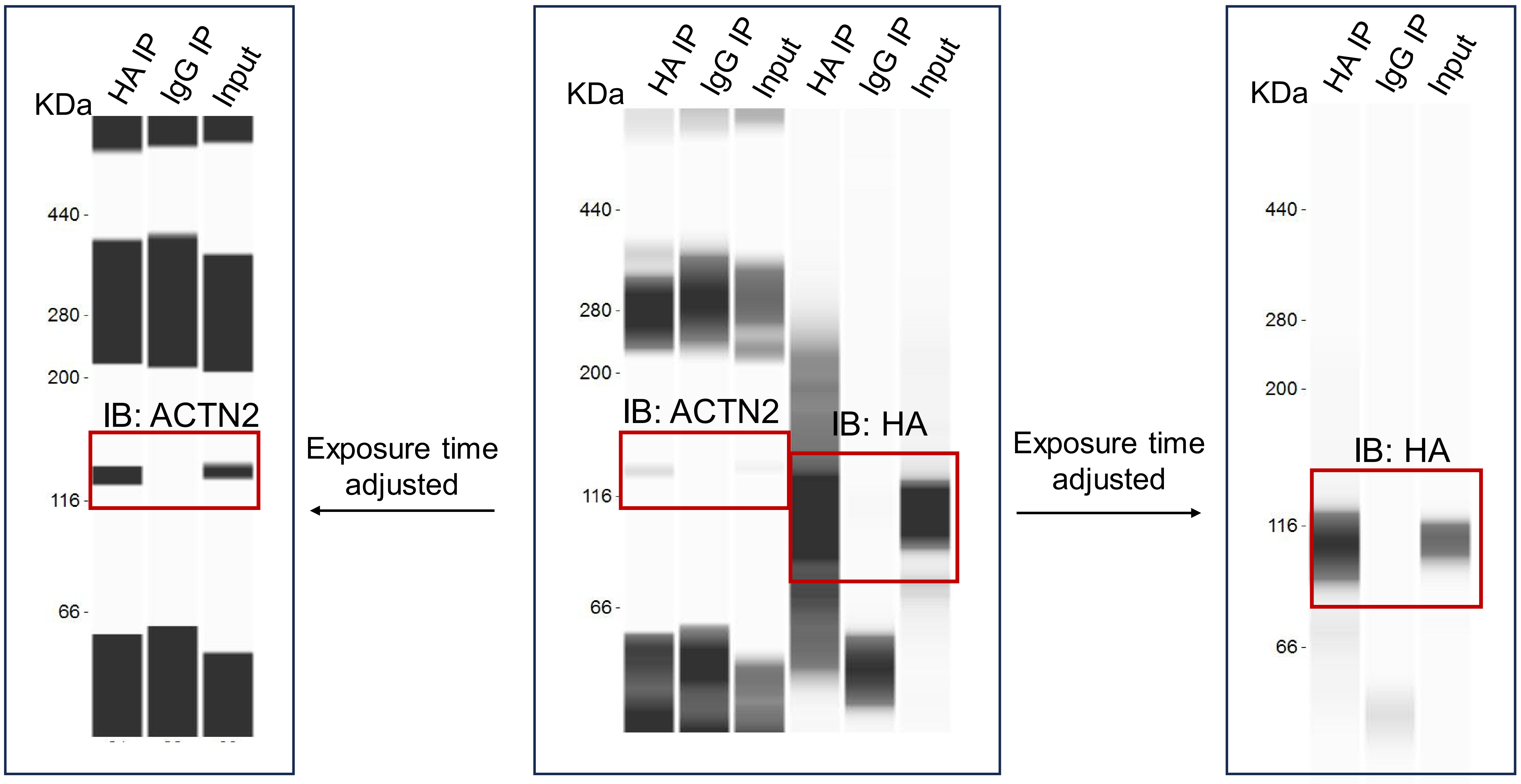
KDa
HA IP
IgG IP
Input
440
280
200
116
66
IB: ACTN2
Exposure time adjusted
KDa
HA IP
IgG IP
Input
HA IP
IgG IP
Input
440
280
200
116
66
IB: ACTN2
IB: HA
Exposure time adjusted
KDa
HA IP
IgG IP
Input
440
280
200
116
66
IB: HA

Fig. 4D-2

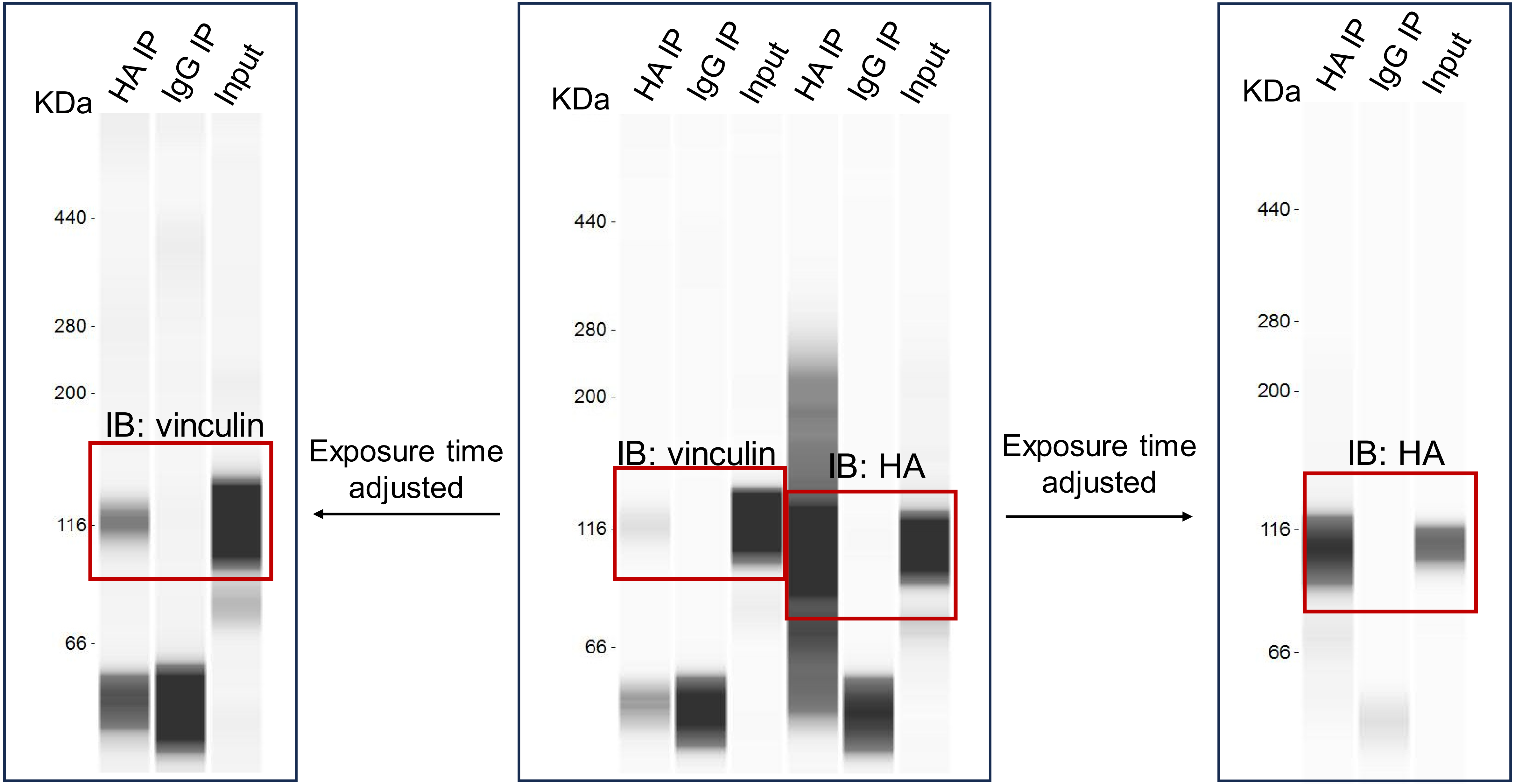
KDa
HA IP
IgG IP
Input
440 -
280 -
200 -
116 -
66 -
IB: vinculin
Exposure time adjusted
KDa
HA IP
IgG IP
Input
HA IP
IgG IP
Input
440 -
280 -
200 -
116 -
66 -
IB: vinculin
IB: HA
Exposure time adjusted
KDa
HA IP
IgG IP
Input
440 -
280 -
200 -
116 -
66 -
IB: HA

Fig. 5F-1

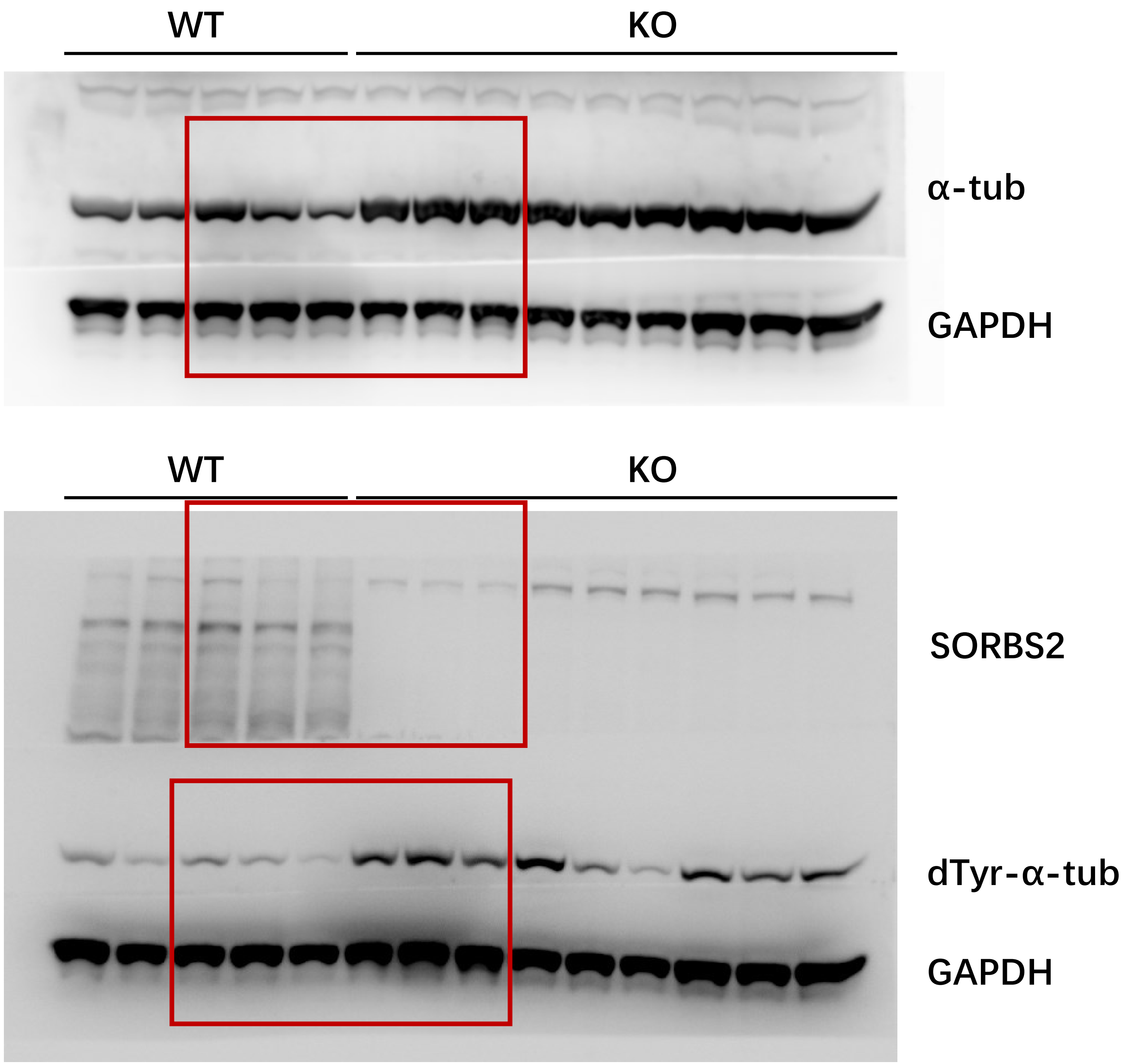

Fig. 5F-2

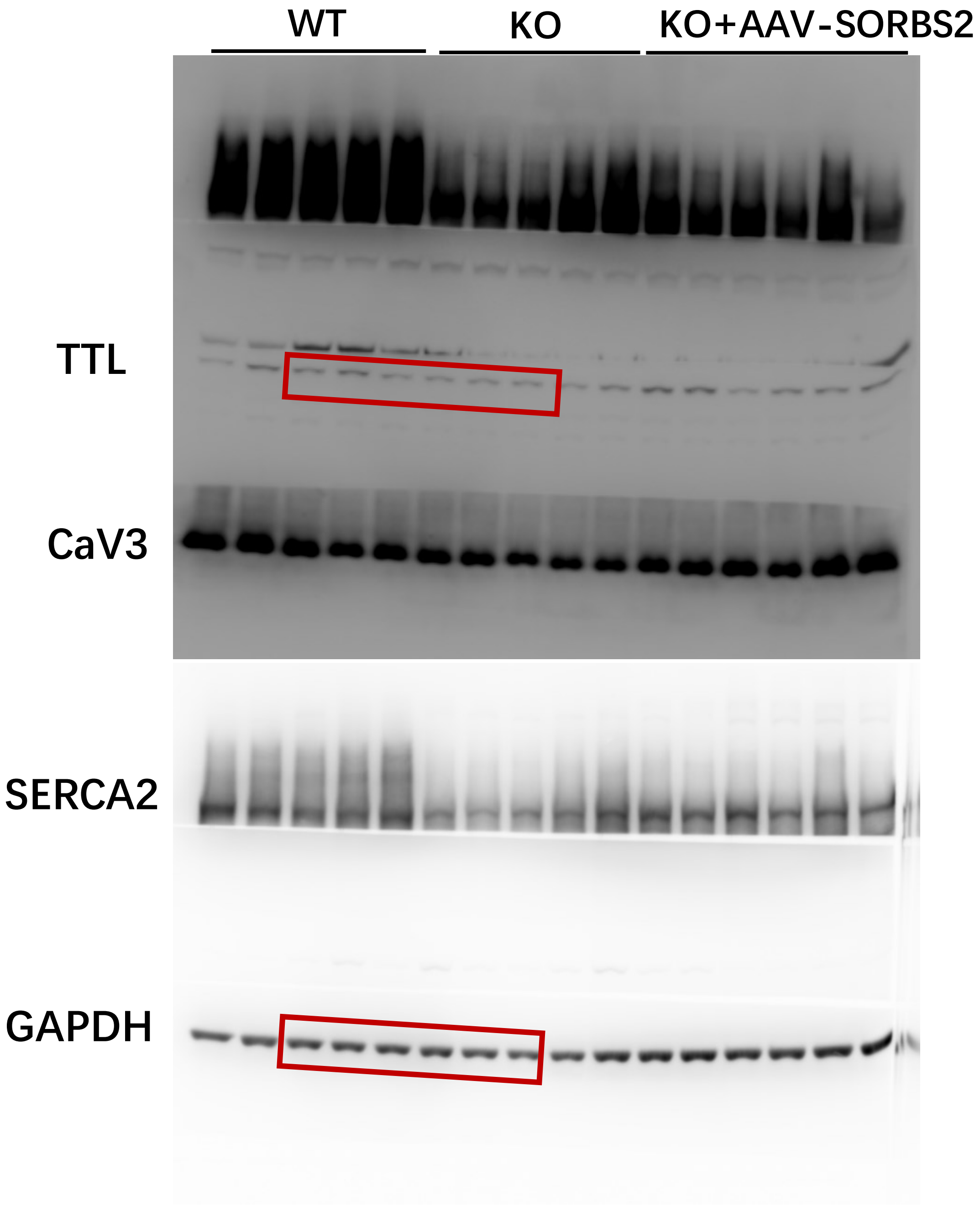

Fig. 6I

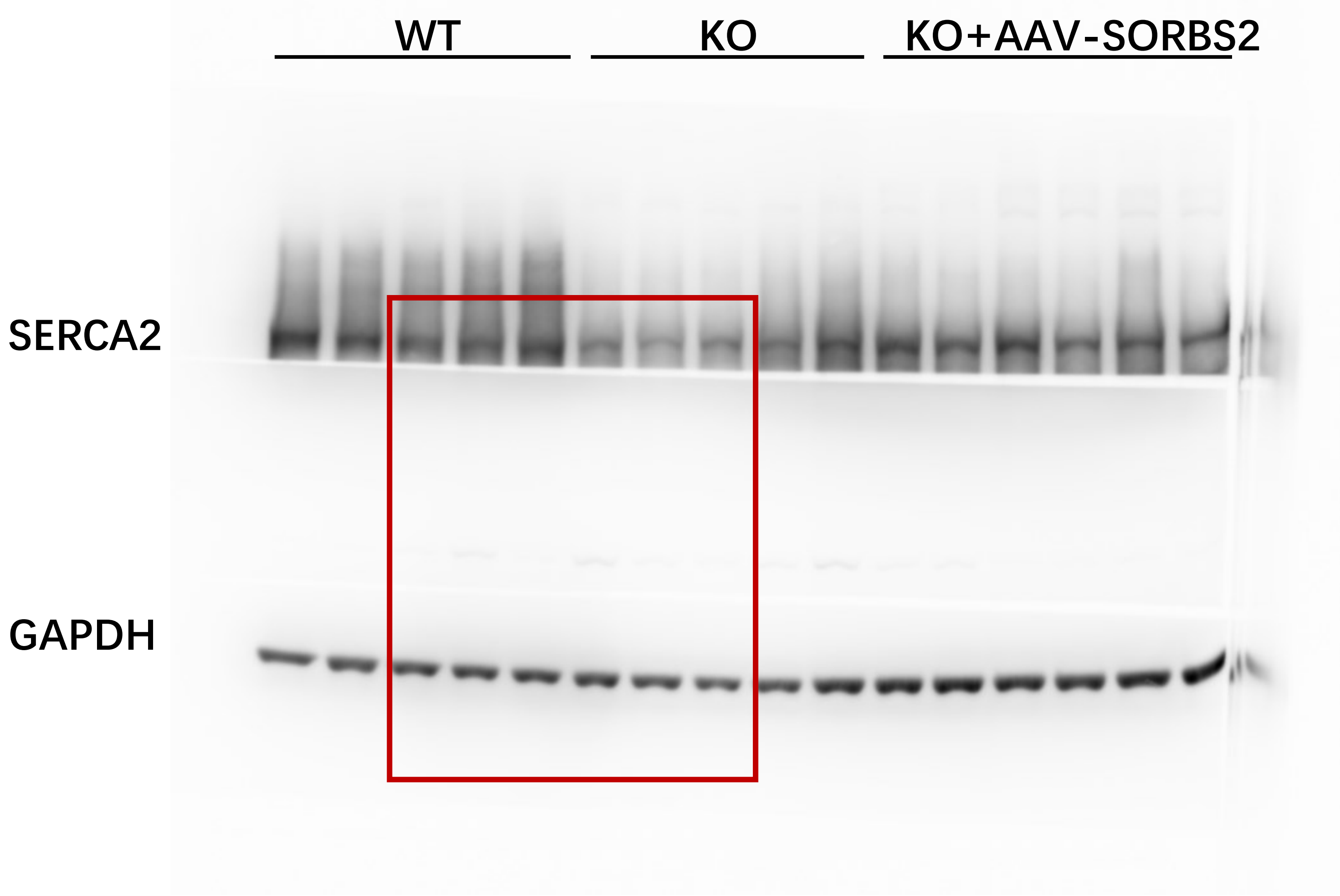

Supplementary Figure 4B-1

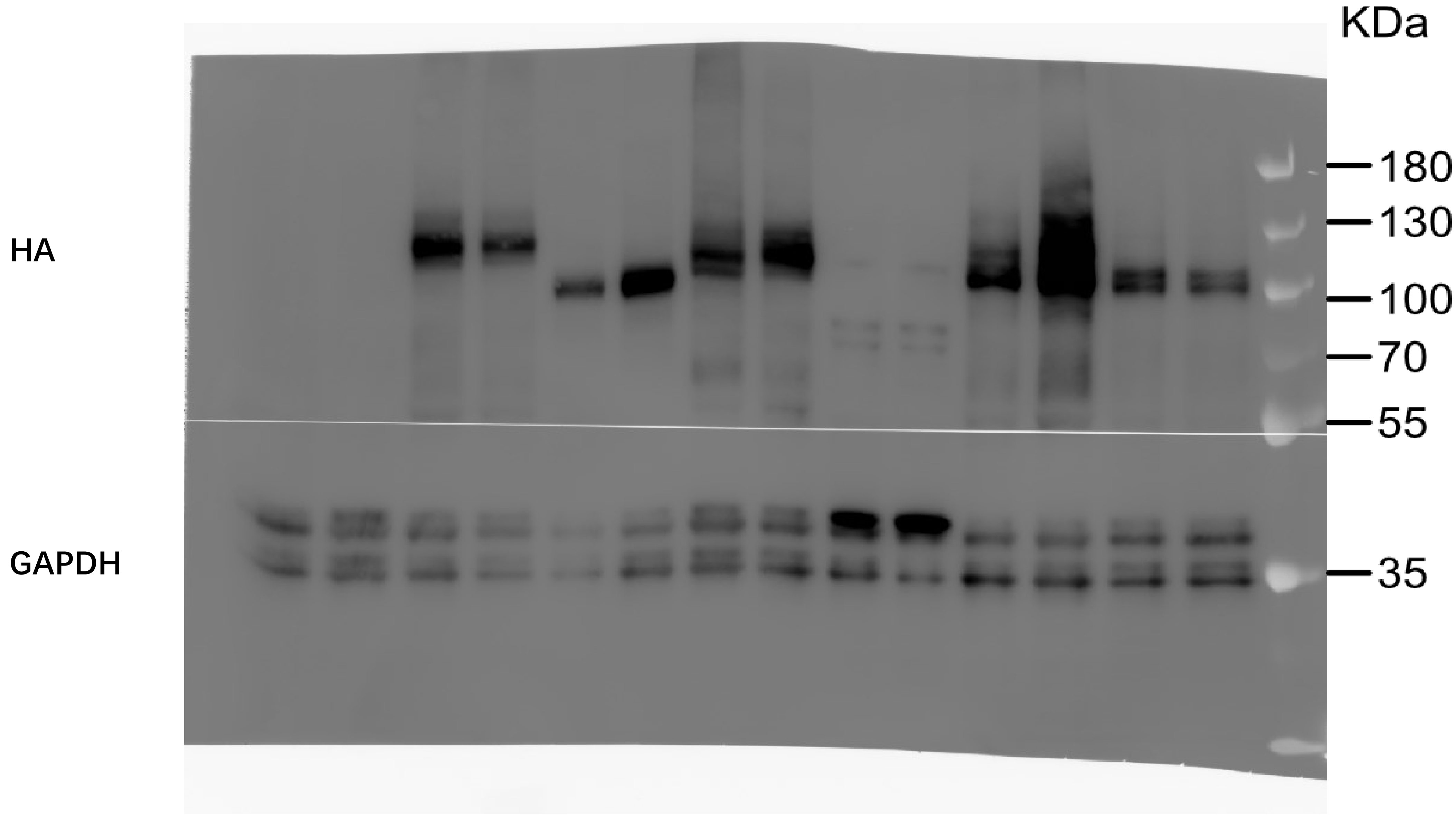

Supplementary Figure 4B-2

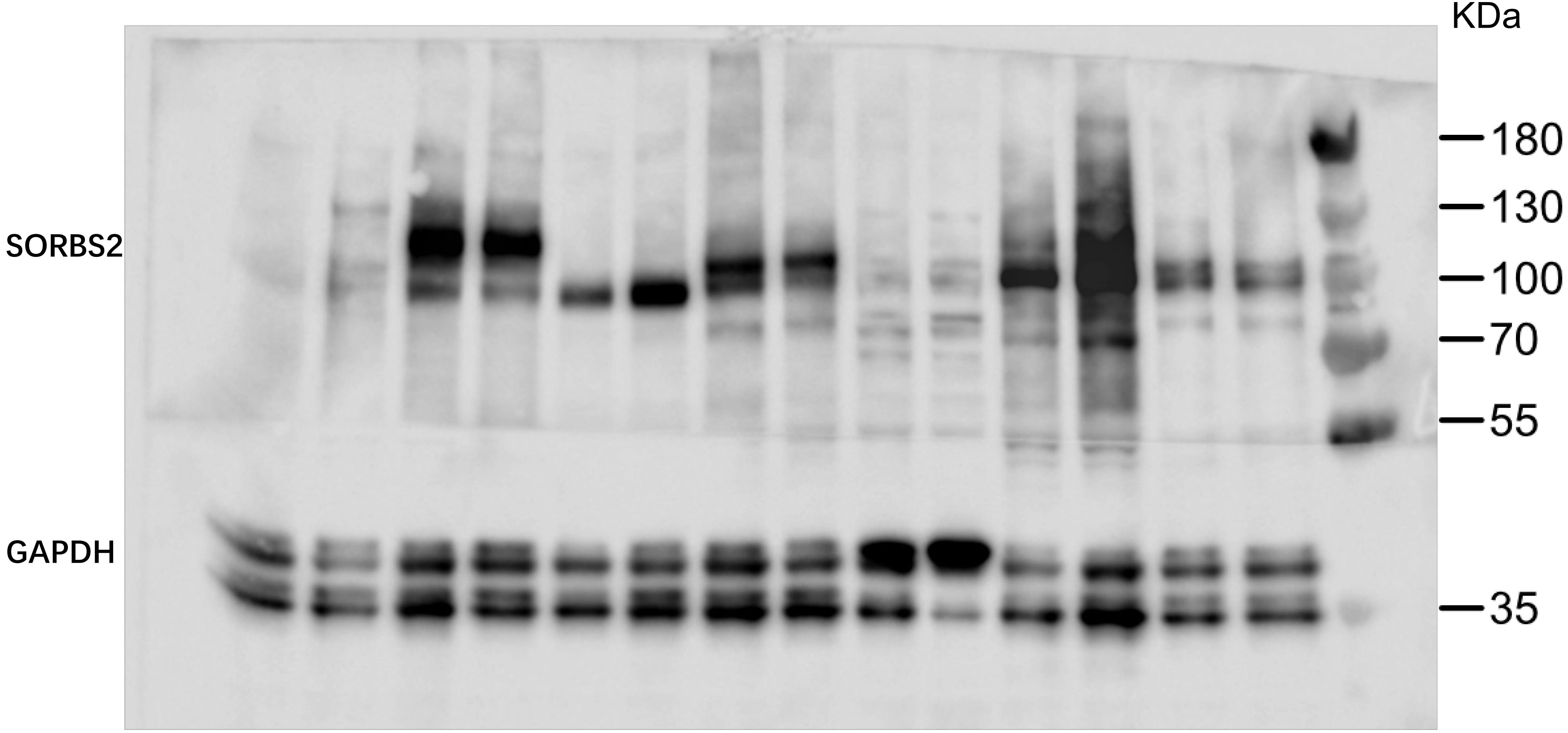

Supplementary Figure 5B

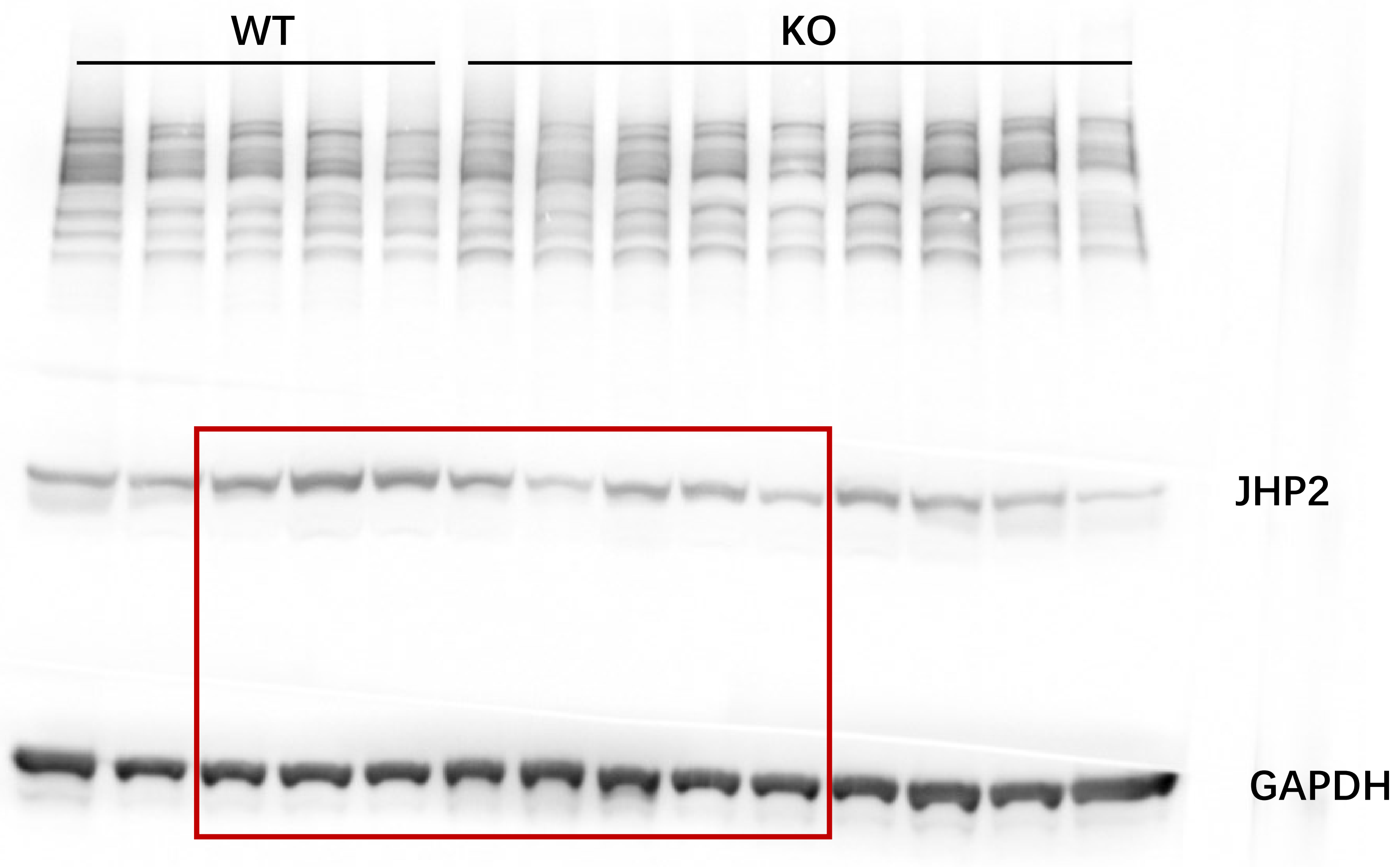

Supplementary Figure 6

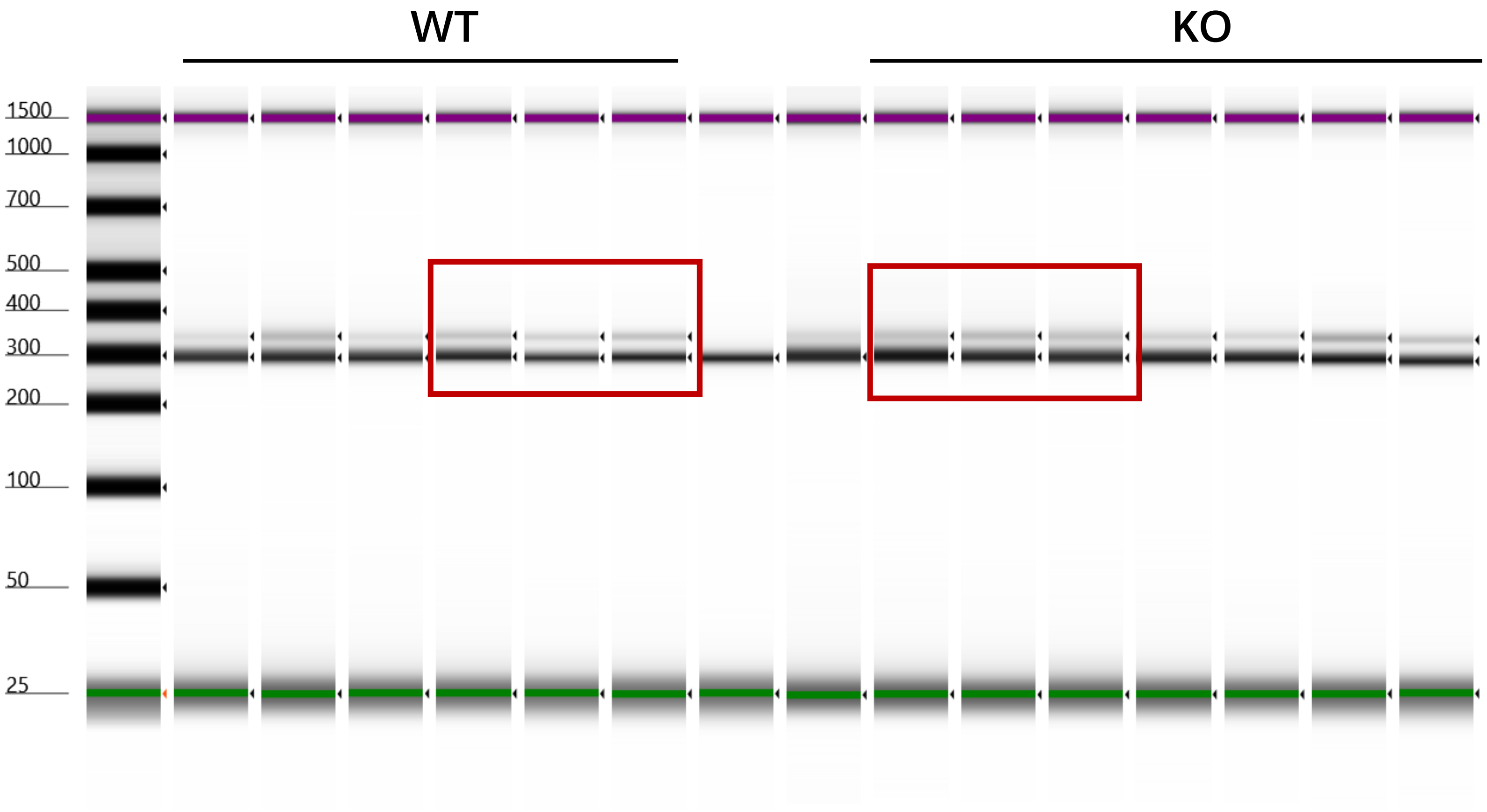

Supplementary Figure 7E

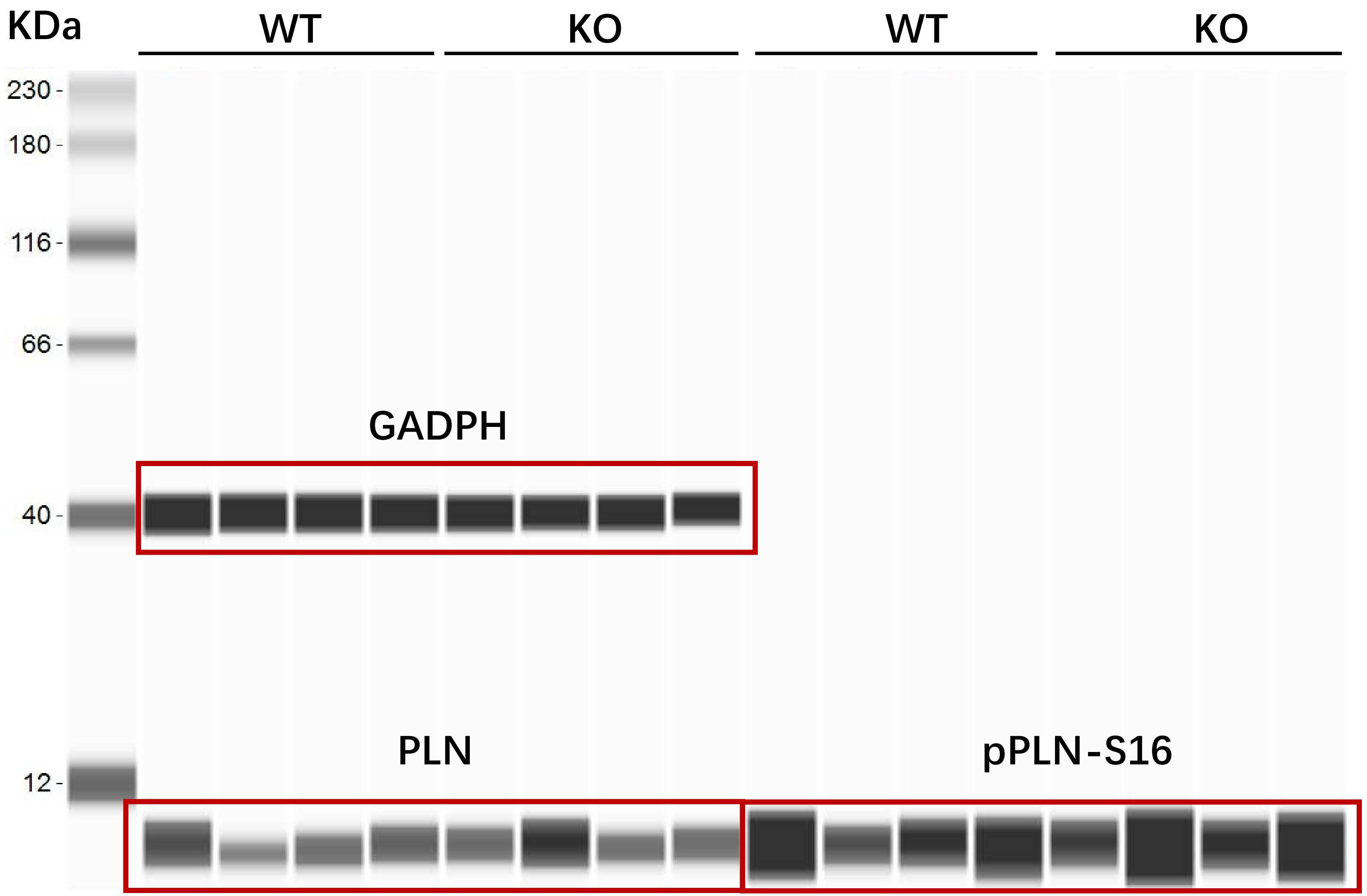

Supplementary Figure 7H

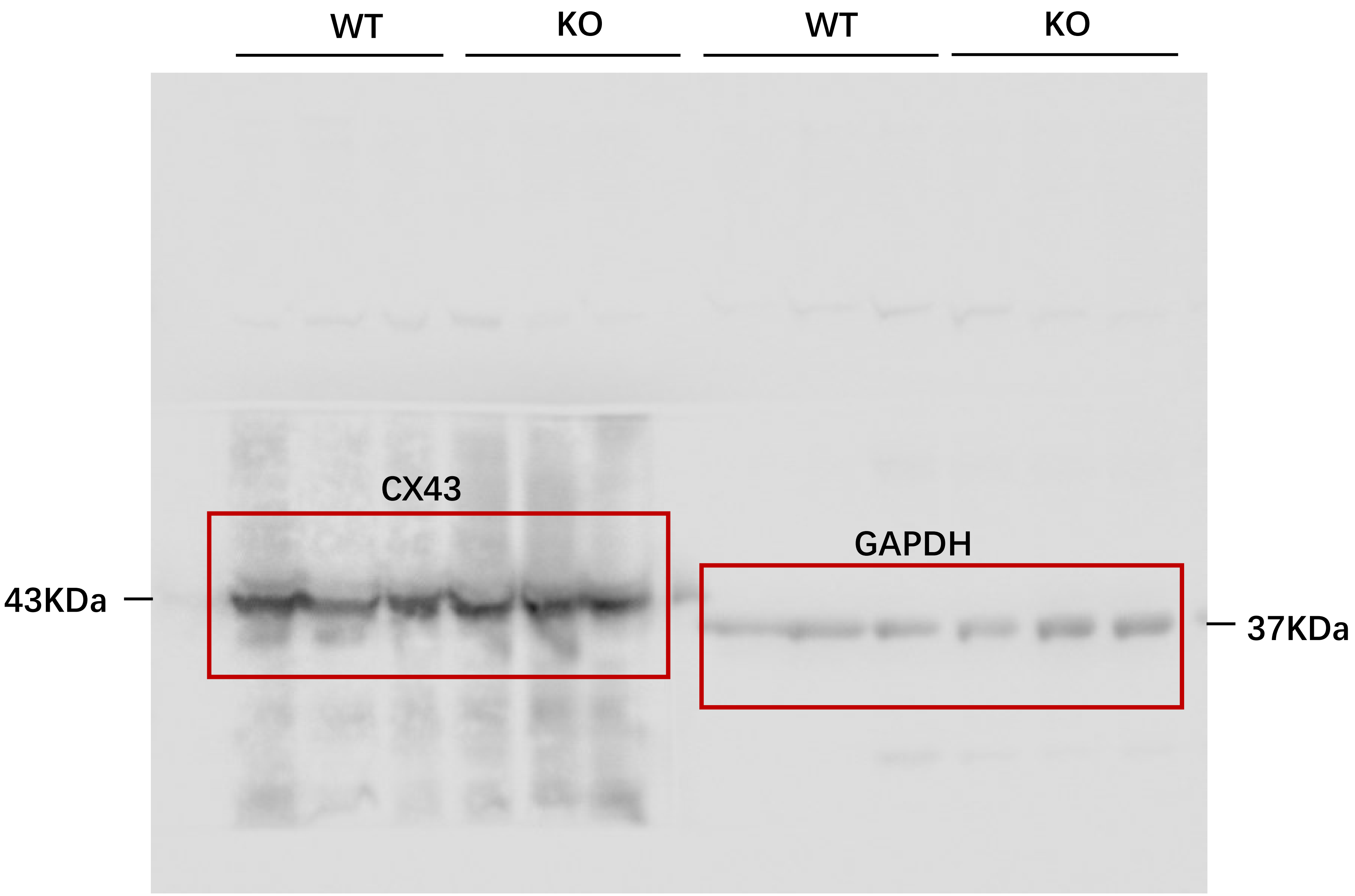

Supplement: Supplementary file 3 [file 42003_2026_10503_MOESM6_ESM.pdf]
