# Supplementary material for: SORBS2 regulates diastolic function through cytoskeletal networks and calcium handling
Source: Commun Biol. 2026 Jun 17;9:1291. doi: 10.1038/s42003-026-10503-6 (PMC13639047; doi:10.1038/s42003-026-10503-6)
Supplement: Supplementary file 4 [file 42003_2026_10503_MOESM8_ESM.pdf]

nature portfolio

Corresponding author(s): William T. Pu

Last updated by author(s): Mar 29, 2026

Reporting Summary

Nature Portfolio wishes to improve the reproducibility of the work that we publish. This form provides structure for consistency and transparency in reporting. For further information on Nature Portfolio policies, see our Editorial Policies and the Editorial Policy Checklist.

Statistics

For all statistical analyses, confirm that the following items are present in the figure legend, table legend, main text, or Methods section.

n/a	Confirmed	
☐	☒	The exact sample size (*n*) for each experimental group/condition, given as a discrete number and unit of measurement
☐	☒	A statement on whether measurements were taken from distinct samples or whether the same sample was measured repeatedly
☐	☒	The statistical test(s) used AND whether they are one- or two-sided<br>*Only common tests should be described solely by name; describe more complex techniques in the Methods section.*
☒	☐	A description of all covariates tested
☐	☒	A description of any assumptions or corrections, such as tests of normality and adjustment for multiple comparisons
☐	☒	A full description of the statistical parameters including central tendency (e.g. means) or other basic estimates (e.g. regression coefficient) AND variation (e.g. standard deviation) or associated estimates of uncertainty (e.g. confidence intervals)
☐	☒	For null hypothesis testing, the test statistic (e.g. *F*, *t*, *r*) with confidence intervals, effect sizes, degrees of freedom and *P* value noted<br>*Give P values as exact values whenever suitable.*
☒	☐	For Bayesian analysis, information on the choice of priors and Markov chain Monte Carlo settings
☒	☐	For hierarchical and complex designs, identification of the appropriate level for tests and full reporting of outcomes
☒	☐	Estimates of effect sizes (e.g. Cohen's *d*, Pearson's *r*), indicating how they were calculated

Our web collection on statistics for biologists contains articles on many of the points above.

Software and code

Policy information about availability of computer code

Data collection	No custom code was used for data collection. Sequencing data were generated using standard instrument control software.
Data analysis	No custom code central to the conclusions of this study was used for data analysis. RNA-seq data were processed and analyzed using publicly available software and packages, including Trim Galore, STAR (v2.7.10), Cufflinks (v2.1.1), the nf-core/differential abundance pipeline (v1.5.0) with DESeq2, and the prcomp function in R. Statistical analyses were performed using GraphPad Prism 9. Additional image and signal quantification was performed using Fiji/ImageJ, Compass for Simple Western, IonWizard, and PV Lab Chart software.

For manuscripts utilizing custom algorithms or software that are central to the research but not yet described in published literature, software must be made available to editors and reviewers. We strongly encourage code deposition in a community repository (e.g. GitHub). See the Nature Portfolio guidelines for submitting code & software for further information.

Data

Policy information about availability of data

All manuscripts must include a data availability statement. This statement should provide the following information, where applicable:

- Accession codes, unique identifiers, or web links for publicly available datasets
- A description of any restrictions on data availability
- For clinical datasets or third party data, please ensure that the statement adheres to our policy

The sequencing data used in this study are available in the GEO database under accession code GSE306787. Reviewer token: "opobwkaqlbetzyv". All data and reagents used in this study can be obtained from the corresponding authors upon reasonable request.

Research involving human participants, their data, or biological material

Policy information about studies with human participants or human data. See also policy information about sex, gender (identity/presentation), and sexual orientation and race, ethnicity and racism.

Reporting on sex and gender	This study did not directly recruit human participants. The re-analysis of publicly available human cardiac single-nucleus RNA sequencing data (Human Heart Cell Atlas) and published human transcriptomic datasets was performed solely to characterize SORBS2 expression patterns across cardiac cell types and disease states. Sex-disaggregated data were not extracted or analyzed from these public datasets, as the analysis focused on gene expression levels rather than sex- or gender-based differences.
Reporting on race, ethnicity, or other socially relevant groupings	Not applicable.
Population characteristics	Not applicable.
Recruitment	Not applicable.
Ethics oversight	Not applicable.

Note that full information on the approval of the study protocol must also be provided in the manuscript.

Field-specific reporting

Please select the one below that is the best fit for your research. If you are not sure, read the appropriate sections before making your selection.

☒ Life sciences ☐ Behavioural & social sciences ☐ Ecological, evolutionary & environmental sciences

For a reference copy of the document with all sections, see nature.com/documents/nr-reporting-summary-flat.pdf

Life sciences study design

All studies must disclose on these points even when the disclosure is negative.

Sample size	Sample sizes were not predetermined by formal power calculations. Group sizes were determined based on prior experience with similar mouse models and are consistent with those commonly used in the field. For in vivo experiments (echocardiography, cardiac catheterization, tissue harvest), groups typically contained n = 5-15 mice per genotype. For in vitro experiments (cardiomyocyte mechanics, calcium imaging, immunofluorescence), at least 3 independent biological replicates (individual mice) were used, with multiple cells measured per replicate. Exact group sizes are reported in the figure legends and Supplementary Tables 1-5.
Data exclusions	No data were excluded from the analyses.
Replication	All experiments were performed with at least three independent biological replicates (individual animals or independent cell preparations), as stated in the Statistical Analysis section. The cardiac phenotype (diastolic dysfunction with preserved systolic function) was confirmed by multiple orthogonal approaches: echocardiography, invasive pressure-volume catheterization, post-mortem gross morphology, lung weight measurement, and histology. Experiments were reproduced across independent mouse cohorts analyzed at multiple time points (6 weeks, 3 months, and 6 months of age). AAV-mediated rescue experiments independently confirmed the cardiomyocyte-intrinsic mechanism. Key molecular findings (increased microtubule detyrosination, decreased SERCA2, reduced JPH2) were confirmed by both immunofluorescence and immunoblotting.
Randomization	Experimental animals were randomly assigned to each experimental/control group. For AAV treatment experiments, littermates from the same litter were randomly assigned to receive AAV-SORBS2, AAV-SORBS2 deletion mutants, or no treatment. Genotyping was performed prior to experimental group assignment. Mice of both sexes were included and distributed across groups.
Blinding	Investigators were blinded to the genotypes of individual animals during experiments and outcome assessments, as stated in the Statistical Analysis section. For echocardiography and cardiac catheterization, measurements were acquired and analyzed by individuals blinded to

genotype. For histological scoring and image quantification, image analysis was performed blinded to genotype. Blinding was maintained until all data were collected.

Reporting for specific materials, systems and methods

We require information from authors about some types of materials, experimental systems and methods used in many studies. Here, indicate whether each material, system or method listed is relevant to your study. If you are not sure if a list item applies to your research, read the appropriate section before selecting a response.

Materials & experimental systems

n/a	Involved in the study
☐	☒ Antibodies
☒	☐ Eukaryotic cell lines
☒	☐ Palaeontology and archaeology
☐	☒ Animals and other organisms
☒	☐ Clinical data
☒	☐ Dual use research of concern
☒	☐ Plants

Methods

n/a	Involved in the study
☒	☐ ChIP-seq
☒	☐ Flow cytometry
☒	☐ MRI-based neuroimaging

Antibodies

Antibodies used

All antibodies used in this study are listed in Supplementary Table 9 of the manuscript. Key antibodies include:
• Anti-SORBS2 (for immunoblotting and immunofluorescence)
• Anti-JPH2 / Junctophilin-2 (for immunoblotting and immunofluorescence; Abcam)
• Anti-Cav1.2 / CACNA1C (for proximity ligation assay)
• Anti-α-tubulin (for immunoblotting and immunofluorescence)
• Anti-detyrosinated α-tubulin (for immunoblotting and immunofluorescence)
• Anti-TTL (tubulin tyrosine ligase; for immunoblotting)
• Anti-SERCA2 (for immunoblotting)
• Anti-phospholamban (PLN) total and anti-phospho-PLN (Ser16) (for immunoblotting)
• Anti-VCL (vinculin; for co-immunoprecipitation)
• Anti-ACTN2 (α-actinin 2; for co-immunoprecipitation and immunofluorescence)
• Anti-N-cadherin and anti-Cx43 (for immunofluorescence of intercalated discs)
• Anti-HA (for immunoprecipitation of HA-SORBS2)
• Alexa Fluor-conjugated secondary antibodies (Invitrogen, 1:200)

Validation

All antibodies were validated by the manufacturers. Immunoblot validation was confirmed by the expected molecular weights. Specificity of the anti-SORBS2 antibody was confirmed using Sorbs2 KO tissue as a negative control (Fig. 1C). Other antibodies were validated using positive controls (e.g., taxol treatment as positive control for detyrosinated α-tubulin) and/or by comparison with established localization patterns from the literature.

Animals and other research organisms

Policy information about studies involving animals; ARRIVE guidelines recommended for reporting animal research, and Sex and Gender in Research

Laboratory animals

Species: Mus musculus (house mouse). Strain: C57BL/6N background. Genotypes: wild type (WT), heterozygous (Sorbs2^Δex8/+^, Het), and homozygous knockout (Sorbs2^Δex8/Δex8^, KO). Sex: both male and female mice were used; survival analysis showed comparable effects in both sexes (Supplementary Fig. 1C). Ages: neonatal (postnatal day 1–2) for cardiomyocyte isolation and AAV injection; 6 weeks, 3 months, 6 months, and 9 months for phenotypic characterization.

Wild animals

This study did not involve wild animals. All animals used were laboratory mice (Mus musculus) maintained under controlled conditions in a specific pathogen-free facility.

Reporting on sex

Survival analysis demonstrated comparable effects in both sexes (Supplementary Fig. 1C). No sex-based differential analysis was performed, as the primary outcome was cardiac phenotype driven by genotype.

Field-collected samples

This study did not involve samples collected from the field. All samples were obtained from laboratory-bred mice housed under controlled conditions (12h/12h light/dark cycle, 22 ± 2°C, 45%–65% humidity) in a specific pathogen-free facility at Boston Children's Hospital.

Ethics oversight

All animal experiments were approved by the Institutional Animal Care and Use Committee (IACUC) of Boston Children's Hospital (BCH), Boston, MA, USA. All procedures were conducted in accordance with the guidelines of the BCH IACUC-approved protocol.

Note that full information on the approval of the study protocol must also be provided in the manuscript.

Plants

Seed stocks	Not applicable.
Novel plant genotypes	Not applicable.
Authentication	Not applicable.
