# Supplementary material for: SORBS2 regulates diastolic function through cytoskeletal networks and calcium handling
Source: Commun Biol. 2026 Jun 17;9:1291. doi: 10.1038/s42003-026-10503-6 (PMC13639047; doi:10.1038/s42003-026-10503-6)
Supplement: Supplementary file 5 [file 42003_2026_10503_MOESM9_ESM.pdf]

nature portfolio

Peer Review File

Sorbs2 Regulates Diastolic Function Through Cytoskeletal Networks and Calcium Handling

Corresponding Author: Dr William Pu

This manuscript has been previously reviewed at another journal. This document only contains information relating to versions considered at Communications Biology.

This file contains all reviewer reports in order by version, followed by all author rebuttals in order by version.

Version 0:

Reviewer comments:

Reviewer #1

(Remarks to the Author)
I thank the authors for the comprehensive response to my comments. I think this journal is a good fit for this study.

Open Access This Peer Review File is licensed under a Creative Commons Attribution 4.0 International License, which permits use, sharing, adaptation, distribution and reproduction in any medium or format, as long as you give appropriate credit to the original author(s) and the source, provide a link to the Creative Commons license, and indicate if changes were made.
In cases where reviewers are anonymous, credit should be given to 'Anonymous Referee' and the source.
The images or other third party material in this Peer Review File are included in the article's Creative Commons license, unless indicated otherwise in a credit line to the material. If material is not included in the article's Creative Commons license and your intended use is not permitted by statutory regulation or exceeds the permitted use, you will need to obtain permission directly from the copyright holder.

To view a copy of this license, visit https://creativecommons.org/licenses/by/4.0/

Reviewers' comments:

Reviewer #1 (Remarks to the Author):

This manuscript by Wu et al explores the role of Sorbs2 in cardiomyocytes and cardiac function. The use of Sorbs2 KO mice along with comprehensive studies using AAV9 induced expression of Sorbs2 WT and mutant proteins, revealed a number of pathological mechanisms leading to diastolic dysfunction. The individual studies on calcium handling, stiffness, fibrosis, and microtubules are well performed, but leaves direct mechanism a little vague. The RNAseq data is helpful as it reinforces these mechanisms. Overall this is a well conducted study that has the potential to be impactful in the field. The overall weakness is a lack of a clear mechanism (it can be multi-factorial, but the study still isn't clear on what is direct and indirect). I provide some comments below to help in this regard.

- The deletion mutants are an interesting approach and provided useful data. However, I think they could provide more mechanistic data, since the different SORBS2 forms have differential rescue. For example, where do the different SORBS2 mutants localize within the cardiomyocyte? Mislocalization in one region or another might give further mechanistic insight into which of SORBS2 direct mechanisms are responsible for the different global or cellular responses.

We thank the reviewer for this insightful suggestion. We agree that defining the subcellular localization of each SORBS2 truncation mutant is essential for interpreting whether impaired rescue reflects loss of intrinsic function or mislocalization from critical cardiomyocyte microdomains.

To address this, we revised the manuscript to include images on the subcellular localization of full-length SORBS2 and each truncation mutant in cardiomyocytes. As shown in revised **Figure S4A**, HA-tagged constructs were co-stained with ACTN2 to mark Z-discs. Both full-length SORBS2 and all truncation mutants displayed a preserved striated pattern and normal alignment with ACTN2. These results indicate that the differential rescue observed among mutants is unlikely due to gross mislocalization with ACTN2, but rather reflects loss of domain-specific functions of SORBS2.

- How do the protein levels and localization change for the direct interactors of SORBS2 as determined by the BirA experiments? This could provide useful information on the direct effect of a loss of SORBS2, and further mechanistic insight.

We identified proteins near SORBS2 by BioID proximity proteomics. In the original manuscript, we focused on how one of these proteins, the dyadic scaffolding protein junctophilin-2 (JPH2), was affected by SORBS2 KO. Immunofluorescence analysis of heart sections and isolated cardiomyocytes revealed disruption of the normal striated dyadic organization of JPH2 (original Fig. S5B-C, revised Fig. S5C–D). In the revised manuscript, we further quantified PH2 levels by western blotting and showed that Sorbs2 KO significantly reduced JPH2 protein. Consistent with these molecular changes, in situ confocal imaging demonstrated impaired T-tubule regularity in

KO hearts (Fig. 4L), indicative of altered dyadic integrity rather than a primary defect in junctional SR structure. Together, these data indicate that loss of SORBS2 compromises the stability and spatial organization of dyadic protein complexes, providing mechanistic insight into how SORBS2 deficiency disrupts calcium microdomain organization.

- While I agree with the authors that the simplest explanation is the most likely, SORBS2 is also expressed in atrial cardiomyocytes. Thus, while the atrial remodeling is likely due to the ventricular phenotype, some data showing the direct impact of SORBS2 in atrial cardiomyocytes would help to support this conclusion.

We agree that SORBS2 is expressed in atrial cardiomyocytes and that a direct atrial effect cannot be excluded. However, echocardiography demonstrates that ventricular diastolic dysfunction precedes overt atrial remodeling in SORBS2 KO mice. At 3 months, KO mice already show impaired diastolic indices (increased mitral E velocity and E/E′), while left atrial size remains unchanged. By 6 months, diastolic dysfunction is more pronounced (markedly elevated E/E′) and is accompanied by significant left atrial enlargement (**Tables S1–2**). Consistently, gross morphology reveals preserved atrial structure at early stages, with left atrial enlargement emerging at ≥6 months and progressing to biatrial enlargement, right ventricular dilation, and frequent left atrial thrombus formation at late stages (**Fig. S3**). Together, these temporal and functional data support secondary atrial remodeling driven by ventricular pathology, rather than a primary atrial defect. Moreover, ventricular-targeted AAV rescue was sufficient to ameliorate atrial remodeling, further supporting this conclusion. Nonetheless, we acknowledge that a cardiomyocyte-autonomous role of SORBS2 in atrial myocytes remains an interesting question and will be explored in future studies using atrial-specific models. We stated this in the limitations section of the revised discussion, "In addition, while ventricular diastolic dysfunction precedes atrial enlargement in this model, a direct atrial cardiomyocyte role of SORBS2 cannot be fully excluded."

- The SORBS2 rescue experiment (Figure 3C) is nice, but the rescued mice still die before 20 months of age, indicating that something still eventually goes wrong in these animals. What do they die from at this time point? Is there any functional data as they age?

We thank the reviewer for raising this important point. We would like to clarify that our approach represents an exogenous add-back of SORBS2 using AAV, rather than genetic correction of the Sorbs2 locus. Despite early delivery of AAV at postnatal day 3 prior to disease onset, rescued mice still do not achieve a normal lifespan, indicating that AAV-mediated add-back is not equivalent to complete genetic restoration. Several non-mutually exclusive factors may contribute to late mortality. First, rAAV-mediated transgene expression, while long-lasting in cardiomyocytes, may partially decline with aging due to epigenetic silencing, cellular stress, or progressive disease burden. Second, SORBS2 is globally deleted in this model, and cardiomyocyte-restricted add-back does not correct potential defects in non-cardiomyocyte

compartments or extracardiac tissues, which may contribute to late-stage pathology. Finally, the level of SORBS2 add-back may not precisely mimic endogenous SORBS2 levels.

Our rescue strategy was designed to allow us to investigate SORBS2 function. Consistent with this goal, rescued mice exhibit sustained improvements in diastolic function and atrial remodeling at intermediate ages, allowing us to use this assay to evaluate the timing, cell type, and domains of SORBS2 essential for its role in these aspects of the cardiac phenotype. While the late death of AAV-SORBS2 treated animals leaves room for additional roles for SORBS2 (e.g. essential developmental or extra-cardiomyocyte roles), our results clearly show the essential role of SORBS2 and its annotated functional domains within cardiomyocytes to maintain cardiomyocyte diastolic function. Evaluating the p0recise cause of death at late stages is outside of the scope of this study and would likely require conditional gene inactivation or re-expression in different tissues and time points. In the revised manuscript, we indicated this limitation of the AAV rescue experiment: "The late death of AAV-Tnnt2-SORBS2-treated KO mice might be due to insufficient AAV-directed SORBS2 expression, or to SORBS2 requirement in development or in other cell types."

- The abstract and introduction center on HFpEF as the background and rationale for the study. However, this is mostly a model of diastolic dysfunction – and the title and discussion better capture that. I think the authors should refocus the abstract and introduction to indicate that while HFpEF is a reasonable extension of this work, it is not the main finding. However, if the authors wished to do additional characterizations of the mouse models to support a HFpEF phenotype, then that is acceptable as well.

We thank the reviewer for this thoughtful and constructive comment. We agree that the primary phenotype observed in the Sorbs2 knockout model is diastolic dysfunction with preserved systolic function, rather than a full recapitulation of the heterogeneous HFpEF syndrome. Accordingly, we have revised the abstract and introduction to more clearly position diastolic dysfunction as the central focus and main finding of this study, while framing HFpEF as a clinically relevant context and potential extension rather than the primary conclusion.

Specifically, we now emphasize that our work identifies SORBS2 as a critical cardiomyocyte-intrinsic regulator of myocardial stiffness, calcium handling, and diastolic function. References to HFpEF are retained to highlight the translational relevance of diastolic dysfunction, but no longer imply that the *Sorbs2* knockout model fully captures the systemic complexity of HFpEF. We believe this reframing more accurately reflects the scope of the data presented and avoids overstating the disease attribution of the model.

Reviewer #2 (Remarks to the Author):

In their article, Wu and colleagues have provided an interesting and mechanistically robust investigation of the role of SORBS2 in cardiomyocytes. They show that this protein is involved in both focal adhesion/costamere and dyadic structure/function, and that it's loss drives HFpEF-like

changes in both the extracellular matrix and cardiomyocyte. The AAV work employed to examine SORBS2-dependent rescue of cardiac function was particular well performed, and showed the importance of the various domain proteins in this task. The article is very well written.

It's very interesting that the authors observed an association between SORBS2 with both focal adhesions and dyads. The implication is made that the SORBS2-containing dyads are internal, and in association with t-tubules. However, dyads are also present at the surface of cardiomyocytes, and it could certainly be the case that the biotinylation experiments have identified these dyadic proteins which are nearby surface costameres. Based on the imaging of SORBS2 by confocal microscopy (Suppl Fig. 1B), it is not clear whether surface or internal proteins have been imaged, or both. Please perform careful confocal Z-stack imaging of SORBS2 to address this point. If SORBS2 is not, in fact, present in internal dyads, it could still follow that loss of surface SORBS2 could disrupt the structure and calcium homeostasis of internal dyads via effects on the cytoskeleton.

We thank the reviewer for this important and technically insightful comment. To directly address whether SORBS2 localizes to internal dyads versus surface-associated dyads/costameres, we performed high-resolution confocal Z-stack imaging using a spinning disk confocal with post-processing deconvolution. This showed that SORBS2 is present at the cell membrane, the intercalated disks, and T-tubules including those deep within the cell. These data are now shown in revised Figure S1C, and the complete Z-stack is provided as Supplementary Data 1.

Related to this point, please correct this sentence: "SORBS2 immunostaining 82 showed that the protein is localized at intercalated discs and in a striated pattern that is consistent with the position of costameres, sarcomere Z-discs, and dyads". With a properly adjusted pinhole, surface costameres and internal dyads are not simultaneously imaged.

We thank the reviewer for pointing this out. We have revised the sentence to avoid implying simultaneous visualization of surface costameres and internal dyads within a single optical plane. The sentence has been rephrased as follows:

"SORBS2 immunostaining revealed enrichment at intercalated discs and a striated pattern consistent with localization to costameres and sarcomere Z-discs."

We also referenced the new confocal Z-stack images in revised Figure S1C and Supplementary Data 1. Furthermore, in the revision we provided additional information on costamere and SORBS2 co-localization with additional imaging of SORBS2 and vinculin (VCL) in Fig. S5B.

The authors are to be commended for performing a careful characterization of in vivo cardiac function, and provide a convincing argument that the SORBS2 KO mice have a HFpEF-like phenotype. Indeed, the mechanisms of diastolic dysfunction provided (fibrosis, diastolic Ca dysregulation, microtubule alterations) are also quite similar to what has been reported in HFpEF. Based on these observations, one might guess that SORBS2 loss is an important mechanism of

HFpEF. However, the authors start the article by showing that SORBS2 is UPREGULATED in human HCM and mouse HFpEF. What could explain this marked mismatch? My own suspicion is that this is because both HCM and mouse HFpEF are frequently associated with growth of t-tubules and dyads. If SORBS2 is present in dyads and necessary for their maintenance, its upregulation would therefore not be surprising. Please address this point in the discussion. If possible, please also include an analysis of SORBS2 expression in human HFpEF for comparison. While discussing your data, it is important to mention that while SORBS2 KO mice look like they have HFpEF this does not mean that there is necessarily loss of the protein in the normal course of this disease.

We thank the reviewer for this insightful comment. We agree that the HFpEF-like phenotype observed in SORBS2 KO mice does not imply that SORBS2 is downregulated during the natural course of HFpEF. Instead, we propose that the upregulation of SORBS2 observed in human HCM and in mouse HFpEF reflects a compensatory response to pathological remodeling of cardiomyocyte microdomains. As suggested by the reviewer, both HCM and HFpEF are frequently associated with expansion and/or remodeling of T-tubules and dyads. Given our data indicating that SORBS2 plays an important role in maintaining the structural integrity and function of these domains, its upregulation in disease settings is consistent with an adaptive attempt to stabilize calcium-handling and cytoskeletal architecture under increased mechanical and metabolic stress. We included this in the revised discussion, “Disease-associated upregulation of SORBS2 in human cardiomyopathy likely reflects a compensatory response to cytoskeletal and microdomain stress; defining when and why this adaptive response fails may reveal new strategies to preserve diastolic homeostasis across diverse cardiac diseases.”

In this context, the SORBS2 KO model reveals the requirement for SORBS2 in preserving diastolic calcium homeostasis, cytoskeletal organization, and myocardial compliance, rather than suggesting that HFpEF necessarily arises from loss of SORBS2 expression. Indeed, SORBS2 upregulation could reflect a compensatory mechanism that counteracts other changes that favor diastolic dysfunction. We have revised the Discussion to explicitly clarify this distinction and to emphasize that the contribution of SORBS2 to human heart disease pathogenesis remains uncertain: “While our study clearly demonstrates the essential role of SORBS2 in maintaining cardiomyocyte diastolic function in mice and SORBS2 upregulation in human heart disease, further studies are required to elucidate SORBS2’s contribution to the pathophysiology of human heart disease, such as heart failure with preserved ejection fraction.”

It’s interesting that the authors observed fibrosis following SORBS2 KO. I assume that they believe that this results from the induction of stress responses in cardiomyocytes that then activate fibroblasts? Please speculate on these mechanisms.

We agree with the reviewer that the fibrosis observed following SORBS2 deletion is most likely secondary to cardiomyocyte-intrinsic stress responses rather than a primary fibroblast-autonomous effect.

Loss of SORBS2 caused diastolic calcium dysregulation, increased cytoskeletal stiffness, and impaired mechanotransduction in cardiomyocytes, all of which are well-established triggers of pro-fibrotic signaling. These alterations are expected to promote the release of paracrine factors from stressed cardiomyocytes, thereby activating cardiac fibroblasts and driving extracellular matrix remodeling. We added this cardiomyocyte-to-fibroblast crosstalk to the discussion as a plausible mechanism linking SORBS2 deficiency to myocardial fibrosis, consistent with existing models of HFpEF pathophysiology.

Reviewer #3 (Remarks to the Author):

These authors set out to elucidate the function of SORBS2 in cardiomyocyte biology. Global knockout of SORBS2, including during development, provoked a phenotype of early mortality. Virus-driven restoration of SORBS2 in cardiomyocytes rescued multiple aspects of the pathological phenotype and significantly increased longevity of mutant mice.

In an effort to glean mechanistic insights into mechanisms whereby SORBS2 functions to maintain myocardial structural and functional homeostasis, the authors conducted 2 different proximity assays followed by immunological pulldown experiments. They uncovered multiple proteins involved in numerous structural and functional aspects of cardiomyocyte function including those involved in focal adhesion structure and function, the actin cytoskeleton, sarcomere and intercalated disk assembly, microtubules, intermediate filaments, and events culminating in fibrosis. In the end, the authors conclude that SORBS2 governs a very wide range of molecular and cellular processes which include, at least, increased fibrosis, both passive and active myocardial stiffness, increased microtubule stability and detyrosination. Active mechanisms governing cardiomyocyte relaxation, such as intracellular Ca2+ homeostasis, are also perturbed in SORBS2 knockouts.

We thank the reviewer for the careful and balanced summary of our work. We agree that SORBS2 influences multiple aspects of cardiomyocyte structure and function, and we appreciate the opportunity to clarify both the mechanistic focus and the conceptual scope of our conclusions.

Our intent is not to suggest that SORBS2 independently governs each of these processes as isolated pathways. Rather, we propose that SORBS2 functions as a **structural and mechanistic integrator** that links cytoskeletal organization, calcium handling, and myocardial mechanical properties. The proximity-based assays and biochemical analyses were therefore designed to define the network of SORBS2-associated proteins that collectively support cardiomyocyte structural and functional homeostasis, rather than to enumerate independent downstream effects. We have revised the text to make this integrative role more explicit and to avoid overinterpretation of breadth as mechanistic multiplicity.

Additional studies based on pharmacological manipulation of tubulin tyrosination pointed to this post-translational modification as playing a significant functional role in SORBS2 knockouts. In sum, the authors conclude that downregulation of microtubule detyrosination in SORBS2 knockouts plays a role. Turning this around, it is inferred that SORBS2 promotes a beneficial DEtyrosination of microtubules (triple negative in the prior sentence). This is never stated explicitly to my reading of the manuscript.

We agree with the reviewer that the role of SORBS2 in regulating microtubules could be stated more clearly. We observed that Sorbs2 ablation increased detyrosinated tubulin, suggesting that *Sorbs2* antagonizes tubulin detyrosination. We clarified this in the revised discussion, "Together, our results suggest that SORBS2 antagonizes microtubule detyrosination, likely indirectly through impaired mechanotransduction signaling at focal adhesions."

The manuscript is very well written, and the figures are clear and easy to follow. The science reported is sound. However, novelty is a significant concern. Multiple other groups have studied the role(s) of SORBS2 in myocardial homeostasis and remodeling, uncovering numerous elements and molecules reported here. Other groups have pointed to a role of this molecule in the control of cytoskeletal organization, calcium handling, matrix remodeling, and cardiomyocyte metabolism. Thus, novelty of the present report is substantially diminished.

We respectfully disagree with the reviewer's assessment that this study lacks novelty:

1. Conceptually, this study links adhesome signaling to the regulation of diastolic function. While the adhesome is known to regulate the cytoskeleton, its role in regulating diastolic function has not been well appreciated.
2. This study emphasizes the importance of Sorbs2 in regulating diastolic function – diastolic dysfunction is the predominant phenotype of the Sorbs2 KO. This is an impactful finding, given the importance of diastolic dysfunction in human heart failure, our limited understanding of its molecular mechanisms, and the scarcity of genetic models. SORBS2 provides a tractable entry point to dissect the mechanisms by which the adhesome regulates diastolic function. While multiple groups have previously studied this gene, they have reported contradictory results and assigned the mouse model different labels. The prior studies did not highlight the severe diastolic dysfunction observed in these mice, which is its predominant feature through ~6 months of age.

We have revised the Discussion to more clearly articulate the advances made in this study and its distinction from prior studies.

It is a huge and in my view inappropriate leap to infer that the multiple – numerous – molecular and cellular changes reported here can be couched as "hallmark features of HFpEF".

We agree with the reviewer that it would be best to refrain from describing this model as having "hallmark features" of HFpEF. Accordingly, we revised the manuscript to avoid this terminology and instead emphasize that the SORBS2 KO phenotype recapitulates key mechanistic components of diastolic dysfunction, which are frequently observed in HFpEF but are not specific to this clinical syndrome. Our intent is not to claim disease specificity, but rather to use the SORBS2-deficient model as a mechanistic tool to interrogate pathways governing myocardial relaxation, stiffness, and structural integrity, which are central determinants of diastolic dysfunction across multiple forms of cardiac disease.

I do not see at all in Figure 1A evidence to support the authors' contention of "upregulation of SORBS2 in…"human HCM and murine HFpEF models". The figure points to upregulation in a wide range of myocardial pathological conditions including HFpEF (HFD+LNAME model) plus HCM, pressure overload from TAC, pressure overload from AngII + PE. It seems this molecule is upregulated in numerous quite diverse pathological contexts. Why single out HFpEF?

We appreciate the reviewer's careful reading of Figure 1A and agree that SORBS2 upregulation is observed across multiple forms of cardiac stress rather than being specific to HFpEF.

We have revised the text to clarify that SORBS2 induction represents a general stress-responsive feature of pathological myocardial remodeling, rather than an HFpEF-specific signature. HFpEF is highlighted as one clinically relevant context in which diastolic dysfunction predominates, but not as a unique disease entity defined by SORBS2 regulation.

As another concern, this molecule appears to be involved in many, many processes, significantly diminishing in my mind the interest and ultimate clinical implications of these findings. It seems its function is foundational, central, to numerous cellular processes. I do not see how targeting this molecule could be envisioned as a potential therapeutic avenue.

We agree that SORBS2 itself is unlikely to represent an immediate therapeutic target, given its foundational role in cardiomyocyte structure and function.

Rather than proposing SORBS2 as a druggable entity, our study defines it as a mechanistic integrator that coordinates cytoskeletal organization, calcium handling, and myocardial mechanical properties. SORBS2 represents a tractable entry point through which we can dissect mechanisms by which the adhesome regulates cardiomyocyte relaxation, including through regulation of microtubule detyrosination, T-tubule and dyad structure, and calcium handling.

We have revised the Discussion to clarify the basic and translational significance of the study:

Conceptual and translational implications

Collectively, our findings define a previously unrecognized role for SORBS2 as a cardiomyocyte-intrinsic determinant of myocardial diastolic function and reveal the adhesome as a critical regulator of cardiomyocyte relaxation. While prior studies have implicated SORBS2 in diverse aspects of cardiomyocyte structure and stress responses, our work provides integrative evidence that SORBS2 governs myocardial relaxation by coupling focal adhesion integrity to cytoskeletal dynamics and calcium microdomain organization through a adhesome–cytoskeleton–calcium signaling axis.

From a translational perspective, although SORBS2 itself is unlikely to represent a direct therapeutic target due to its foundational structural role, its loss exposes downstream, more selectively targetable mechanisms—particularly microtubule post-translational modifications and cytoskeletal–calcium coupling—that directly regulate myocardial stiffness and relaxation. Disease-associated upregulation of SORBS2 in human cardiomyopathy likely reflects a compensatory response to cytoskeletal and microdomain stress; defining when and why this adaptive response fails may reveal new strategies to preserve diastolic homeostasis across diverse cardiac diseases.

The fact that the authors studied a global, total body knockout of SORBS is another shortcoming.

We agree that the use of a global *Sorbs2* knockout represents a limitation of the current study. However, this limitation was largely mitigated through cardiomyocyte-directed AAV-mediated re-expression of SORBS2. This substantially rescued cardiac structure, function, and survival, supporting a cardiomyocyte-intrinsic requirement for SORBS2 in maintaining myocardial homeostasis.

We now explicitly acknowledge this limitation in the Discussion and note that future studies using cell type–specific genetic models will be important to further dissect non-cardiomyocyte contributions.
